# Highly regioselective and diastereoselective synthesis of novel pyrazinoindolones *via* a base-mediated Ugi-*N*-alkylation sequence†

**DOI:** 10.1039/d3ra02065g

**Published:** 2023-06-06

**Authors:** Maryam Tajik, Morteza Shiri, Faiq H. S. Hussain, Yazdanbakhsh Lotfi Nosood, Behnaz Baeiszadeh, Zahra Amini, Rahman Bikas, Anna Pyra

**Affiliations:** a Department of Organic Chemistry, Faculty of Chemistry, Alzahra University Vanak Tehran 1993893973 Iran mshiri@alzahra.ac.ir; b Medical Analysis Department, Applied Science Faculty, Tishk International University Erbil Kurdistan Region Iraq; c Department of Chemistry, Faculty of Science, Imam Khomeini International University Qazvin 34148-96818 Iran; d Faculty of Chemistry, University of Wrocław 14 Joliot-Curie 50-383 Wroclaw Poland

## Abstract

An efficient base-mediated/metal-free approach has been developed for the synthesis of 1-oxo-1,2,3,4-tetrahydropyrazino[1,2-*a*]indole-3-carboxamide derivatives *via* intramolecular indole N–H alkylation of novel bis-amide Ugi-adducts. In this protocol the Ugi reaction of (*E*)-cinnamaldehyde derivatives, 2-chloroaniline, indole-2-carboxylic acid and different isocyanides was designed for the preparation of bis-amides. The main highlight of this study is the practical and highly regioselective preparation of new polycyclic functionalized pyrazino derivatives. This system is facilitated by Na_2_CO_3_ mediation in DMSO and 100 °C conditions.

## Abstract 2

An efficient base-mediated/metal-free approach has been developed for the synthesis of 1-oxo-1,2,3,4-tetrahydropyrazino[1,2-*a*]indole-3-carboxamide derivatives *via* intramolecular indole N–H alkylation of novel bis-amide Ugi-adducts.

## Graphical abstract

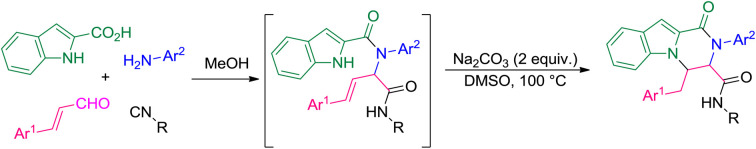

## Introduction

Nitrogen-containing heterocycles are really important structural motifs with large applications in medicinal,^1,2^ pharmaceutical,^3^ and synthetic organic chemistry.^4^ Among them the indole ring is present in many important natural products^5,6^ and is a basic skeleton of a number of drugs.^2,7^ Additionally, fused indole rings have the affinity to bind to receptors.^8,9^ Hence, the preparation of fused and derived indoles has attracted much attention from organic chemists for many years.^10–13^

Indole and pyrrole fused-piperazine and piperazinone derivatives are significant heterocycle compounds used as pharmacophores due to their potential medicinal activities.^14,15^ Investigations on these tricyclic nucleuses started in 1994, when they were studied from two points of view: synthetic and potency in medical science.^16–18^ In this class, pyrazino[1,2-*a*]indolones are very important tricyclic backbones, such as indole joined into a piperazinone ring. These analogues are under great consideration due to their therapeutic uses.^19^

**Fig. 1:**
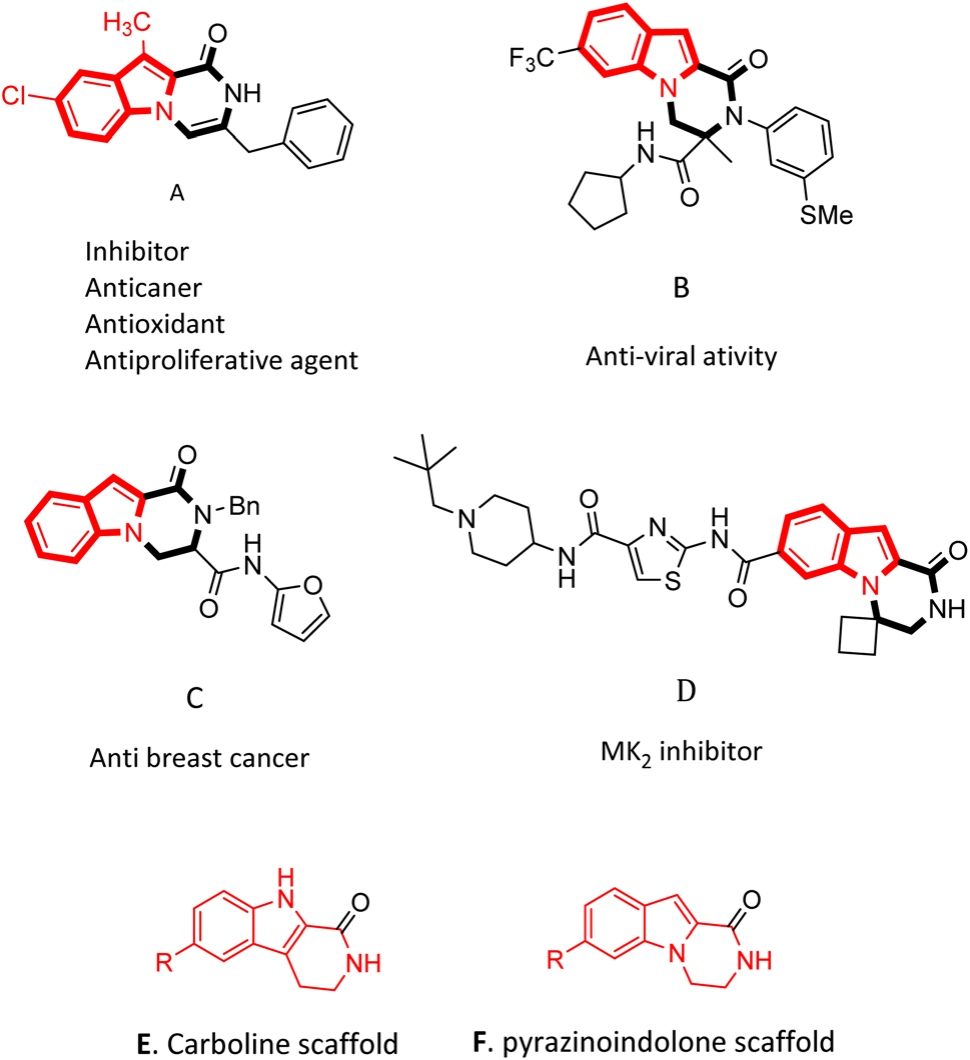
A library of biologically active pyrazino[1,2-*a*]indol-1-one derivatives.

Pyrazino[1,2-*a*]indol-1-one derivatives are impressive pharmacophores in the treatment of various diseases.^19^ Several of them have potent inhibitory^19^ and anti-cancer activities (Fig. 1A, C and D),^10,19,20^ and some of them have anti-infection,^21^ anti-allergenic^22^ and anti-viral properties (Fig. 1B).^23^ Compounds containing pyrazinoindolone frames have been reported as potent mitogen-activated protein kinase-2 (MK_2_) inhibitors.^24,25^

Despite the importance of indole-fused structures, a few effective procedures have been reported for their synthesis. A series of substituted-3,4-dihydropyrazinoindol-1(2*H*)-one was synthesized by Toche *et al.*^26^ from the cyclization reaction of ethyl 1-(2-bromoethyl)-1*H*-indole-2-carboxylate and primary amines. In another work, Youssif *et al.*^10^ prepared open-chain indole-2-carboxamides and then performed consequential oxidation and cyclodehydration using PTSA which resulted in the production of benzyl pyrazino[1,2-*a*]indol-1(2*H*)-ones. In 2017, Kim *et al.*^20^ reported on the preparation of biologically active 1-oxo-1,2,3,4-tetrahydropyrazino[1,2-*a*]indole-3-carboxamide analogs in three steps.

Because of the application of pyrazinoindolones in drug discovery, designing a novel and efficient pathway for their synthesis is tempting enough for synthetic chemists. Currently, Ugi four-component reaction has provided good conditions to synthesize diverse N-heterocyclic structures.^27–29^ This name reaction in combination with other reactions is converted to a remarkable tool to prepare new fused scaffolds.^30^ Since the beginning of the 21st century, various examples have been reported using this combinatory protocol – as post-Ugi process. These strategies include Ugi/Heck,^31,32^ Ugi/Diels–Alder,^33^ Ugi/Buchwald–Hartwig,^34^ Ugi/Knoevenagel condensation,^35,36^*etc.*

There are a few examples for the synthesis of pyrazinoindolones using Ugi reaction. A modified Ugi-type reaction was developed by Ilyn group^37^ to obtain novel indole-fused 1-oxo-1,2,3,4-tetrahydropyrazines based on a cyclization key step. An Ugi-3CC strategy was presented in 2012, for the synthesis of *N*-alkyl-3-oxo-2-aryl-1,2,3,4-tetrahydropyrazino[1,2-*a*]indole-1-carboxamide derivatives. It was the first use of 2-(2-formyl-1*H*-indol-1-yl)acetic acid in the aforementioned reaction.^38^

## Results and discussion

In continuation of our efforts to indole chemistry and multi-component reactions,^39–41^ we focused on the preparation of tetrahydro-pyrazino[1,2-*a*]indol-1-ones using the post-transformation of Ugi adducts, under metal-free conditions.

Despite the positive effect of the presence of transition metals on the yield of many organic reactions^42,43^ these reactions suffer from some limitations such as the high costs of metals and their removal from the reaction medium.^29^ Considering these disadvantages and the low efficiency of the procedure in the absence of transition metals, we have reported an efficient base-mediated strategy for the preparation of new 1-oxo-1,2,3,4-tetrahydropyrazino[1,2-*a*]indole-3-carboxamides.

In the first step, the Ugi condensation of aromatic aldehydes like cinnamaldehyde derivatives, anilines, 1*H*-indole-2-carboxylic acid and diverse isocyanides led to the formation of bis-amides in methanol (Table 1).

**Table 1:** Synthesis of Ugi-adducts^a^^,^^b^^,^^c^

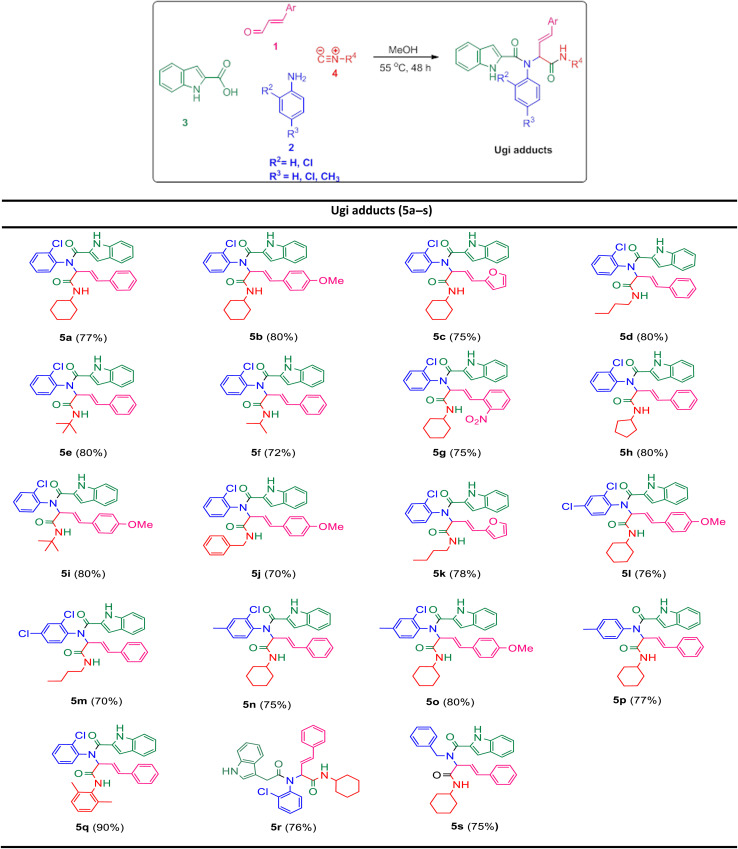

a Reaction conditions: enals (1 mmol), anilines (1 mmol), 1*H*-indole-2-carboxylic acid (1 mmol) and isocyanides (1.1 mmol) in 5 mL methanol at reflux for 48 h.

b Yields are reported for isolated products.

c For 5r we used indole-3-acetic acid instead of indole-2-carboxylic acid.

**Scheme 1:**
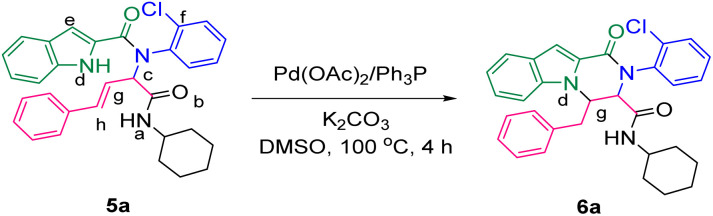
Oxo-tetrahydro-pyrazino[1,2-*a*]indole-3-carboxamides synthesis. The nucleophilic sites are marked as (a–e), while the leaving groups are marked as (f–h).

The prepared bis-amide products are unique due to the presence of several potent nucleophilic sites (Scheme 1a–e) and the leaving groups (Scheme 1f–h) that are located on Ugi products, so they are highly predisposed to intramolecular condensation. Because of the presence of these active sites, we tried to do a post sequential reaction of these Ugi adducts. Initially, the reaction was studied with 5a (derived from cinnamaldehyde, 2-chloroaniline, 1*H*-indole-2-carboxylic acid and cyclohexyl isocyanide) as the model in the presence of Pd(OAc)_2_/Ph_3_P, K_2_CO_3_ under CH_3_CN reflux conditions, but we did not obtain any products (Table 2, entry 1). Changing the solvent to DMSO, provided 6a with 80% yield (Table 2, entry 2). After the identification of the product, it was determined that only indole N–H alkylated one was formed among the possible products (based on their nucleophilic and electrophilic positions) (Scheme 1).

**Table 2:** Scope of optimization conditions for the synthesis of 6a^a^

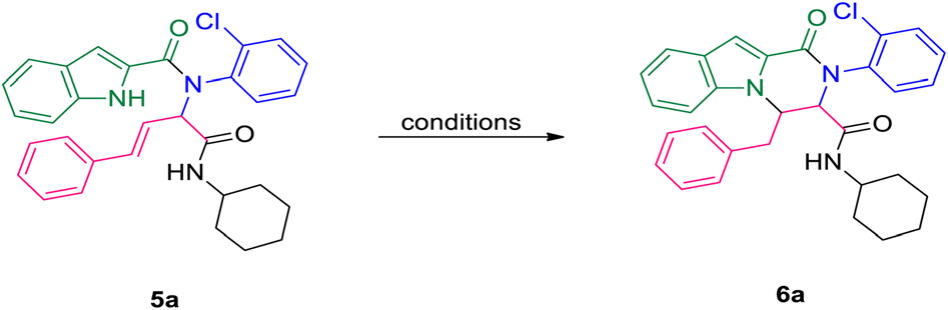						
Entry	Cat/ligand	Solvent	Base	Temp (°C)	Time (h)	Yield^b^ (%)
1	Pd(OAc)_2_/Ph_3_P	CH_3_CN	K_2_CO_3_	82	4	—
2	Pd(OAc)_2_/Ph_3_P	DMSO	K_2_CO_3_	120	4	80
3	Pd(OAc)_2_/Ph_3_P	DMSO	Na_2_CO_3_	120	4	80
4	PdCl_2_/Ph_3_P	DMSO	Na_2_CO_3_	120	4	75
5	CuI/l-proline	DMSO	Na_2_CO_3_	120	4	76
6	—	DMSO	Na_2_CO_3_	120	4	78
**7**	**—**	**DMSO**	**Na**_**2**_ **CO**_**3**_	**100**	**4**	**78**
8	—	DMSO	Na_2_CO_3_	50	4	50
9	—	DMSO	Na_2_CO_3_	Rt	4	25
10	—	1,4-Dioxane	Na_2_CO_3_	100	4	—
11	—	PhCH_3_	Na_2_CO_3_	100	4	—
12	—	DMF	Na_2_CO_3_	100	5	63
13	—	DMSO	K_2_CO_3_	100	4	75
14	—	DMSO	Cs_2_CO_3_	100	4	70
15	—	DMSO	KO*t*-Bu	100	4	70
16	—	DMSO	KOH	100	4	76
17	—	DMSO	Et_3_N	100	5	46
18	—	DMSO	DABCO	100	5	46
19	—	DMSO	—	100	4	32

a Conditions: 5a (0.5 mmol), metal (10 mol%), ligand (20 mol%), base (2 equiv.), solvent (4 mL).

b Isolated yields.

The *N*-alkylation and arylation of indole are a noteworthy tool for carbon–nitrogen bond formation, because of the vast application of N-heterocycles in natural products and bioactive drugs.^44–46^ To date, there have been several attempts at designing mild strategies towards *N*-alkylated indoles, under metal-catalyzed or catalyst-free conditions.^46–48^

To continuation the optimization process, different metals/ligands like PdCl_2_/Ph_3_P and CuI/l-proline were studied, but no preference was observed compared to Pd(OAc)_2_/Ph_3_P (Table 2, entries 4 and 5).

Surprisingly, performing the reaction in the absence of metal/ligand did not show a significant decrease in the product yield (Table 2, entry 6, 78% yield for 6a). In the next step, different temperatures of 50 °C, to 120 °C were investigated. According to the results, there was no remarkable yield difference between the reactions at 100 and 120 °C, but decreasing the temperature to 50 °C or to RT lowered the yields of the reactions (Table 2, entries 6–9). Then, the influence of diverse solvents was examined and the presence of DMSO showed the best effectiveness (Table 2, entries 7 and 10–12). Some alkaline metal bases were examined in order to comprehend the effect of the base nature. The metal bases mentioned in Table 2 (Na_2_CO_3_, K_2_CO_3_, Cs_2_CO_3_, KOH, KO*t*-Bu) showed very good positive results (entries 13–16), but a negative effect on yield was observed in the presence of triethylamine and diazabicyclo [2,2,2] octane (DABCO) as the organic bases, negative effect on the yield was observed (Table 2, entries 17 and 18). Additionally, performing the reaction in the absence of base led to the desired product 6a with the yield of 32% (Table 2, entry 19).

The scope of this Ugi-*N*-alkylation sequence process was explored under optimized reaction conditions (2 equiv. of Na_2_CO_3_ as base, DMSO as solvent, 100 °C temperature, 4 h) (Table 2, entry 7). The results are summarized in Table 3.

**Table 3:** Synthesis of tricyclic products 6*via* a post-Ugi cyclization^a^

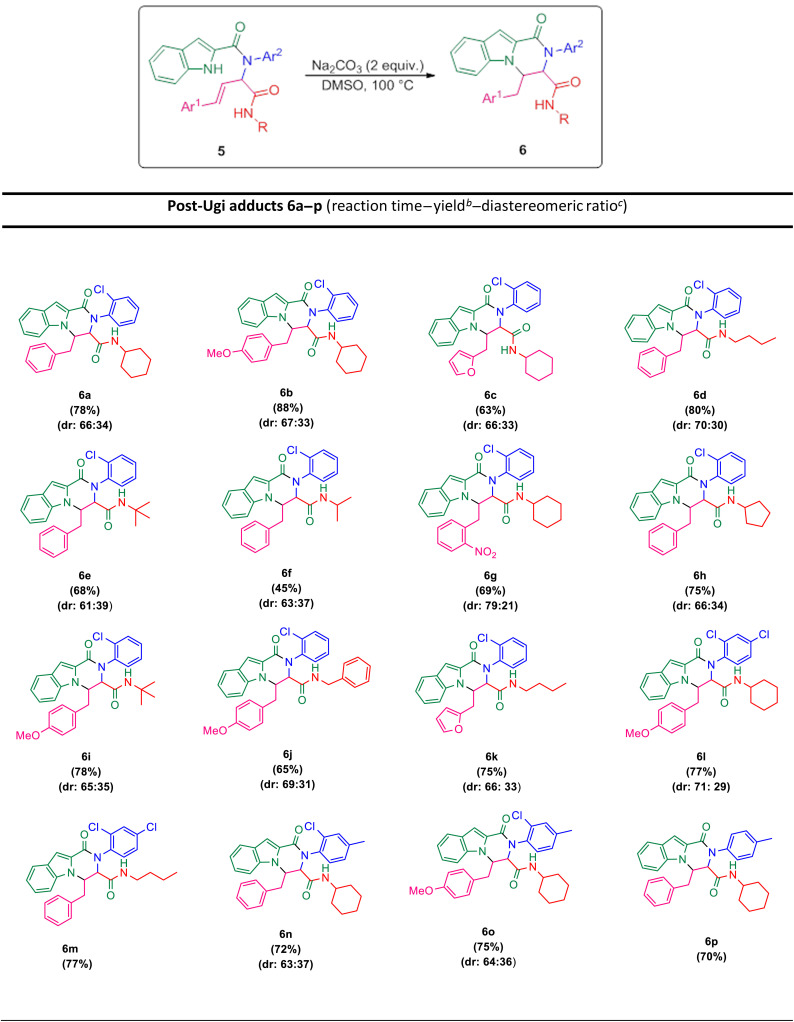

a Reaction conditions: 5 (0.5 mmol), Na_2_CO_3_ (1 mmol) in 2.5 mL DMSO at 100 °C.

b Isolated yields.

c Diastereomeric ratio was reported from the ^1^H NMR spectra.

Ugi products (Table 3) containing electron-withdrawing and electron-donating substituents, reacted under optimum conditions to produce the desired product with moderate to excellent yield. Generally, the best results were obtained using electron-donating groups on cinnamaldehyde part (6b), and less bulky groups on isocyanide part (6d). Ugi products with bulky isocyanide like isopropyl and *t*-butyl showed less reaction efficiency (6e, 6f). Product 6j was also separated with excellent yield, but because of its low solubility in DMSO it could not be identified.

In order to investigate the possibility of the reaction in the absence of chlorine substitution on the aniline ring, we applied *p*-toluidine as amine instead of 2-chloroaniline in the Ugi reaction and product 5p was obtained. This substrate, was treated with Na_2_CO_3_ under optimized reaction conditions and generated post-Ugi product 6p with good efficiency.

Due to the presence of two chiral centers, two diastereomeric isomers were formed in the corresponding products, except for the compounds 6m and 6p, in which only one diastereomer was obtained. It is worth mentioning that indole N–H alkylation was successfully performed without protection of the second N–H function group. This issue is evident from the elimination of the indole N–H peak in the ^1^H NMR spectrum.

**Fig. 2:**
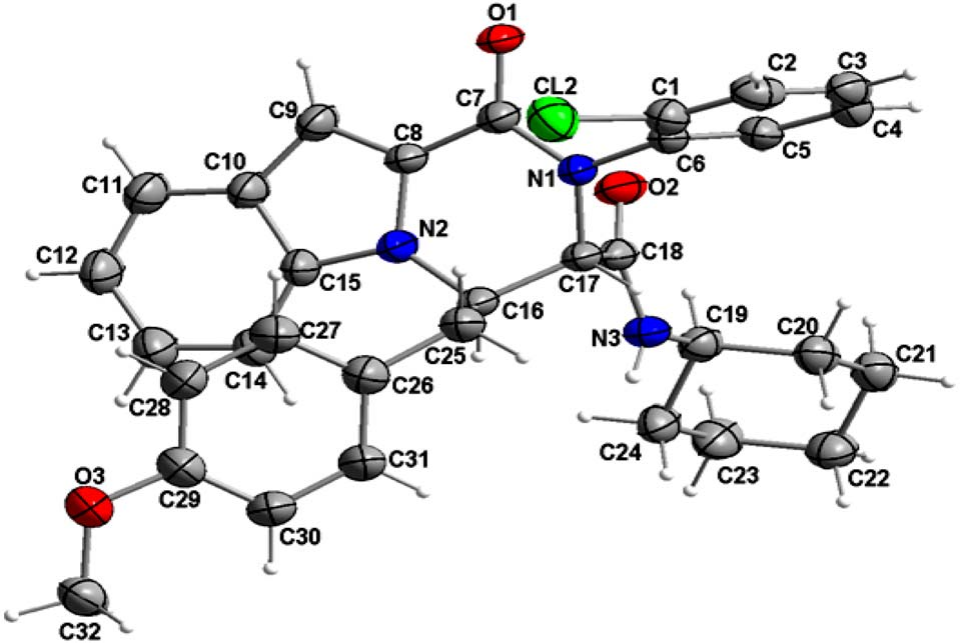
Crystal structure of the 6b molecule showing the atom-numbering scheme and displacement ellipsoids drawn at the 50% probability level. The partially disordered molecule A was removed for clarity.^51^

**Fig. 3:**
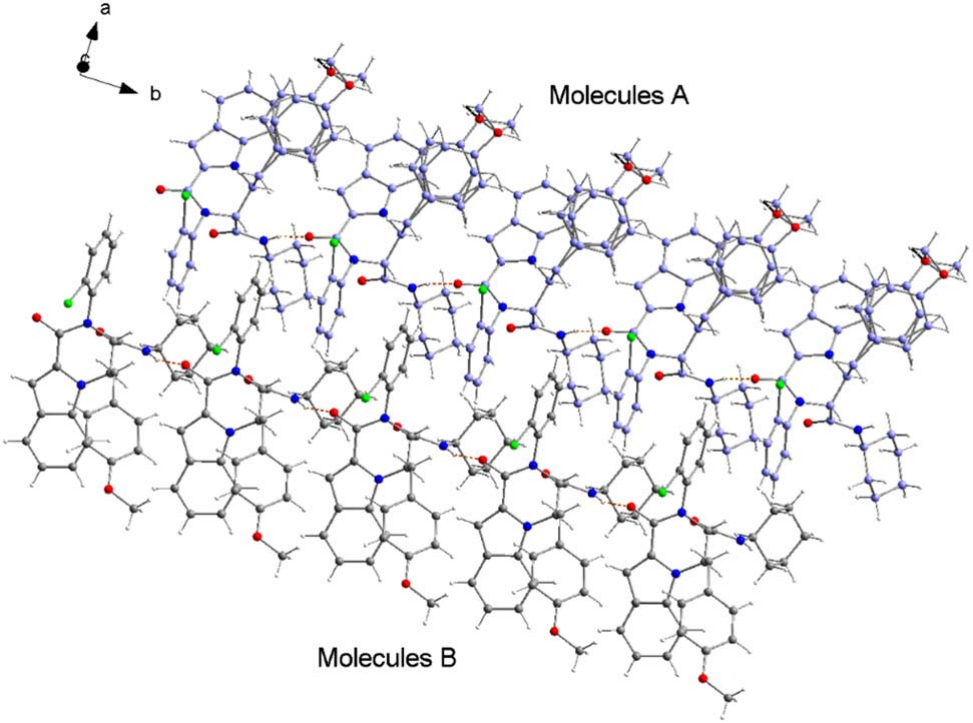
A part of crystal packing of 6b showing the chain along *b*-axis formed by N–H⋯O hydrogen bonds (depicted as orange dashed lines). The carbon atoms are shown in gray for molecules B and in lilac for the partially disordered molecules A.

The structure of compound 6b was determined by X-ray crystallographic analysis to get a better view about the composition structure of these products. The X-ray diffraction data analysis revealed that 6b got crystallized in monoclinic space group *P*2/*n*. The asymmetric unit is composed of two 6b molecules (A and B) but, one of them (A) is partially disordered and thus only one molecule (B) is presented in Fig. 2. In the crystal structure, the 1*H*-indole-2-carboxylic, 2-chloroaniline and cinnamaldehyde derivative (4-methoxybenzene) rings are flat. The 2-chloroaniline and the 4-methoxybenzene rings are rotated from the plane of 1*H*-indole-2-carboxylic ring by 82.36(2) and 35.43(2)°, respectively. The planes of 2-chloroaniline and the 4-methoxybenzene are set at an angle of 64.41(2)° to each other. The Cremer and Pople ring-puckering parameters^49^*Q*, *θ* and *ϕ* (0.58 Å, 1.47°, 358.65° and 0.58 Å, 177.87°, 127.99° for molecule A and B, respectively) calculated using program PLATON^50^ for six-membered *N*-cyclohexylamide ring indicate its chair conformation. In the crystal structure, the molecules of 6b are linked into chains running along the *b*-axis by N3–H3⋯O1^i^ hydrogen bond [H⋯A = 2.01 Å, D⋯A = 2.867(5) Å, D–H⋯A = 164°, (i) *x*, *y* + 1, *z*] involving the atom H3 of the *N*-cyclohexylamide NH group as a donor and the carbonyl O1 atom of 1*H*-indole-2-carboxylic group as an acceptor (Fig. 3).

The analysis using PLATON^50^ showed aromatic π–π stacking interactions were observed in the 6b crystal between the centroids of 2-chloroaniline [C1A–C6A (Cg1A), C1B–C6B (Cg1B)] rings [Cg1A⋯Cg1B = 3.6965(17) Å, Cg1A⋯Cg1B^i^ = 3.7408(17) Å, Cg1A^ii^⋯Cg1B = 3.7408(17) Å, (i) *x*, *y* + 1, *z*, (ii) *x*, *y* − 1, *z*]. A comparatively short distance was also found between the centroids of the 1*H*-indole-2-carboxylic [C10B–C15B (Cg2)] rings from the adjacent molecules [Cg2⋯Cg2^iii^ = 3.5383(16) Å, (iii) *x*, *y* + 1, *z*]. The crystal structure of 6b compound is also stabilized by a few weak C–H⋯π interactions between the C14 atoms of the six-membered 1*H*-indole-2-carboxylic rings and the centroids of the 4-methoxybenzene [C26–C31 (Cg3)] rings: C14A–H14A⋯Cg3A [H⋯A = 2.93 Å, D⋯A = 3.4549(16) Å, D–H⋯A = 116°], C14B–H14B⋯Cg3B [H⋯A = 2.92 Å, D⋯A = 2.9991(15) Å, D–H⋯A = 105°]. As mentioned earlier, molecule A is partially disordered in the 6b crystal structure and this disorder includes the 4-methoxybenzene ring (C26C–C31C, Cg3C). Thus, the following C–H⋯π interaction was also observed: C14A–H14A⋯Cg3C [H⋯A = 2.86 Å, D⋯A = 3.2470(15) Å, D–H⋯A = 106°]. Additionally, the atoms of the ordered (molecule B) methoxy group of 4-methoxybenzene part are also involved in a weak C–H⋯π contact with the centroid of the five-membered 1*H*-indole-2-carboxylic [N2A–C15A (Cg4A)] ring: C32B–H32E⋯Cg4A^iv^ [H⋯A = 2.94 Å, D⋯A = 3.7385(17) Å, D–H⋯A = 139°, (iv) *x* − 1/2, −*y* + 1, *z* − 1/2].

**Scheme 2:**
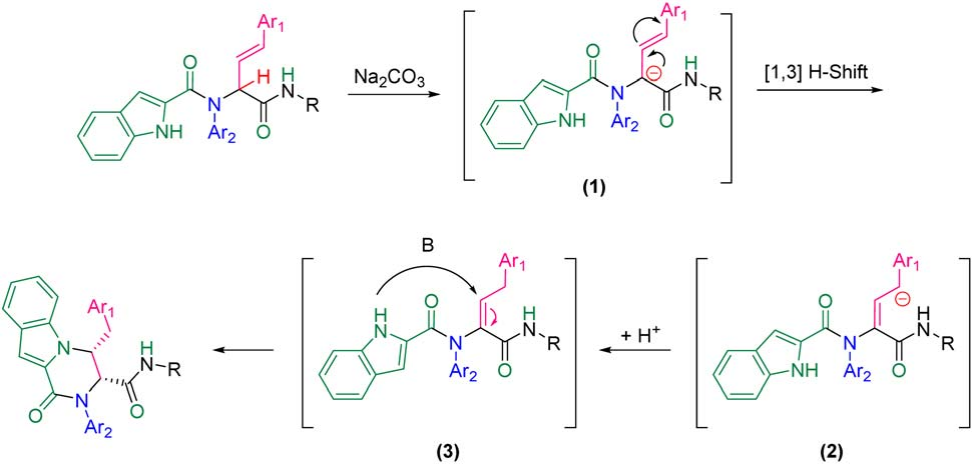
A plausible mechanism for the cyclization step of Ugi adducts.

A plausible mechanism for this diastereoselective cyclization is presented in Scheme 2 based on the experimental results. First, carbanionic intermediate (1), is formed by the addition of base to Ugi substrate. The destabilization of carbanion (1) followed by neutralization led to intermediate (3). This intermediate can undergo intramolecular nucleophilic attack by the nitrogen atom of the indole moiety to generate the final product *via* a 6-*endo-trig* cyclization.

## Conclusion

In summary, we have reported a new sequential Ugi reaction/NH-alkylation for the synthesis of novel tricyclic oxo-tetrahydropyrazino[1,2-*a*]indole-3-carboxamide derivatives. Various enals, anilines and isocyanides were subjected to this protocol and the products were obtained with high selectivity. Simple procedure, high yield of all products, chemo- and diastereoselectivity and metal-free condition are the main advantages of this method that can make it a practical synthetic route for the preparation of pyrazinoindolone derivatives.

## Conflicts of interest

There are no conflicts to declare.

## Supplementary Material

RA-013-D3RA02065G-s001

RA-013-D3RA02065G-s002
