# Supplementary material for: Highly regioselective and diastereoselective synthesis of novel pyrazinoindolones via a base-mediated Ugi-N-alkylation sequence
Source: RSC Adv. 2023 Jun 6;13(25):16963–9. doi: 10.1039/d3ra02065g (PMC10243185; doi:10.1039/d3ra02065g)
Supplement: RA-013-D3RA02065G-s001 [file RA-013-D3RA02065G-s001.pdf]

Electronic Supplementary Material (ESI) for RSC Advances.
This journal is © The Royal Society of Chemistry 2023

Supporting Information

Highly Regio- and Diastereoselective Synthesis of Novel Pyrazinoindolones via a Base-Mediated Ugi-*N*-Alkylation Sequence

Maryam Tajik,[a] Morteza Shiri,*,[a] Faiq H. S. Hussain,[b] Yazdanbakhsh Lotfi Nosood,[a] Behnaz Baeiszadeh,[a] Zahra Amini,[a] Rahman Bikas,[c] Anna Pyra,[d]

[a]*Department of Organic Chemistry, Faculty of Chemistry, Alzahra University, Vanak, Tehran 1993893973, Iran. E-mail:* mshiri@alzahra.ac.ir

[b]*Medical Analysis Department, Applied Science Faculty, Tishk International University, Erbil, Kurdistan Region - Iraq*

[c]*Department of Chemistry, Faculty of Science, Imam Khomeini International University, Qazvin 34148-96818, Iran*

[d]*Faculty of Chemistry, University of Wrocław, 14 Joliot-Curie, 50-383 Wroclaw, Poland*

Experimental

General

Chemicals and solvents were purchased from Fluka, Merck, and Sigma-Aldrich companies and used without further purification. Melting points were measured with an Electro-thermal 9100 apparatus and are uncorrected. IR spectra were recorded with a Shimadzu-IR 460 spectrophotometer. The ^{1}H-NMR and ^{13}C-NMR of products were recorded on a Bruker 600 and 150 MHz or 300 and 75 MHz spectrometer. High-resolution mass spectrometry (ESI-HRMS) measurements were obtained on an Agilent Q-TOF LC-MS, a Thermo Scientific Advantage and a Thermo Scientific Executive spectrometer.

General procedure for Ugi-adduct preparation (5a-s)

After half an hour, to a stirred solution of cinnamaldehyde derivatives **1** or (1 mmol) and aniline **2** (1 mmol) in 5 mL MeOH, 1-H-indole-2-carboxylic acid **3** (1 mmol) and corresponding isocyanide **4** (1.1 mmol) were added respectively. The resulting mixture was stirred at reflux for 48 hours. The reaction progress was monitored by thin-layer-chromatograghy (TLC). Then, the precipitate was filtered off, washed with mixture of ethyl acetate:*n*-hexane (3:7) and dried. Finally, desired Ugi-4CR products **5a-r** were obtained with 70-90% yield.

General procedure for NH alkylation step (6a-p post-Ugi products)

To a stirred mixture of Ugi adduct (**5a-r**, 0.5 mmol) in DMSO (2.5 mL), Na_2CO_3 (2 equiv., 1 mmol) was added. The mixture was stirred at 100 ^{o}C for 4 hours. After completion, which was monitored by TLC, the reaction mixture was cooled to room temperature, diluted with saturated brine (20 mL) and was extracted with dichloromethane (3 ×20 mL). The combined organic layers were dried over sodium sulfate and the solvent was evaporated under reduced pressure. Then, the product was purified by column chromatography on silica gel (n-hexane:ethyl acetate, 3:1) to give pure **6a-p** compounds**.**

(E)-N-(2-Chlorophenyl)-N-(1-(cyclohexylamino)-1-oxo-4-phenylbut-3-en-2-yl)-1H-indole-2-carboxamide (5a).

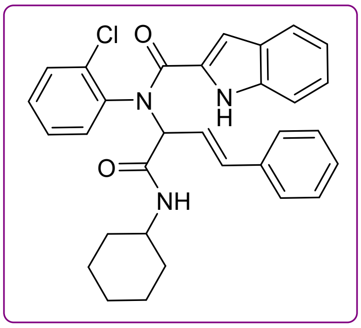

pale yellow powder (**Yield:** 77%), **mp:** 110-112 °C.

HRMS (ESI) m/z: Calcd for $C_{31}H_{30}ClN_3O_2$ $[M+H]^+$ 512.2105, Found 512.2126.

^{1}H NMR (600 MHz, CDCl$_3$) δ ppm: 9.36 (s, 1H, NH), 7.88-7.83 (m, 1H), 7.52 (dd, *J* = 7.8, 1.4 Hz, 1H), 7.48-7.44 (m, 2H), 7.44-7.40 (m, 1H), 7.37-7.32 (m, 3H), 7.26-7.16 (m, 4H), 7.00 (t, *J* = 7.6 Hz, 1H), 6.74-6.67 (m, 1H), 6.30 (d, *J* = 7.5 Hz, 1H), 6.03 (dd, *J* = 15.8, 7.6 Hz, 1H, =CH), 5.57 (d, *J* = 9.3 Hz, 1H, =CH), 5.27-5.17 (m, 1H), 3.86-3.79 (m , 1H), 2.03-1.96 (m , 1H), 1.95-1.87 (m , 1H), 1.73-1.66 (m, 2H), 1.64-1.54 (m, 2H), 1.40-1.32 (m, 2H), 1.27-1.18 (m, 2H).

^{13}C NMR (150 MHz, CDCl$_3$) δ ppm: 168.6, 162.2, 137.6, 137.1, 136.0, 135.4, 135.1, 132.6, 130.6, 130.4, 129.1, 128.7, 128.5, 128.2, 127.8, 126.7, 124.9, 122.4, 121.1, 120.3, 111.5, 106.5, 64.5, 48.7, 32.9, 25.5. 24.7.

N-(2-Chlorophenyl)-N-(1-(cyclohexylamino)-4-(4-methoxyphenyl)-1-oxobut-3-en-2-yl)-1H-indole-2-carboxamide (5b).

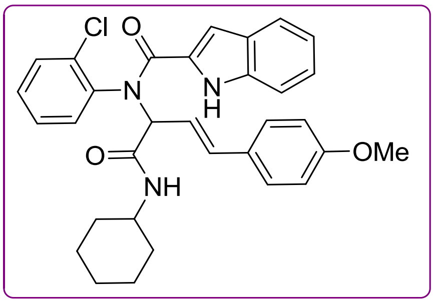

pale yellow powder (**Yield:** 80%), **mp:** 198-200 °C.

HRMS (ESI) m/z: Calcd for $C_{32}H_{32}ClN_3O_3$ $[M+H]^+$ 542.2210, Found 542.2226.

^{1}H NMR (600 MHz, CDCl$_3$) δ ppm: 9.54 (s, 1H, NH), 7.76 (d, *J* = 7.3 Hz, 1H), 7.34 (d, *J* = 7.9 Hz, 1H), 7.25-7.15 (m, 4H), 7.04 (t, *J* = 7.6 Hz, 1H), 6.93 (d, *J* = 8.2 Hz, 1H), 6.84 (t, *J* = 7.4 Hz, 1H), 6.69 (d, *J* = 8.2 Hz, 1H), 6.60 (d, *J* = 8.4 Hz, 2H), 6.52-6.47 (m, 1H), 6.31-6.24 (m, 1H), 5.71 (dd, *J* = 15.3, 9.2 Hz, 1H, =CH), 5.50 (d, *J* = 9.2 Hz, 1H, =CH), 5.13-5.07 (m, 1H), 3.71-3.64 (m, 1H), 3.61 (s, 3H, OMe), 1.79 (d, *J* = 6.6 Hz, 1H), 1.77 -1.69 (m, 1H), 1.54 -1.46 (m, 2H), 1.40 (d, *J* = 12.8, 1H), 1.21-1.12 (m, 2H), 1.04-0.93 (m, 3H).

^{13}C NMR (150 MHz, CDCl$_3$) δ ppm: 169.0, 162.3, 159.7, 137.7, 136.6, 135.2, 134.1, 132.7, 130.5, 130.4, 129.2, 128.8, 128.2, 128.0, 127.8, 124.8, 122.3, 120.3, 118.8, 113.9, 111.7, 106.6, 64.6, 55.2, 48.7, 32.9, 25.5, 24.8.

(E)-N-(2-chlorophenyl)-N-(1-(cyclohexylamino)-4-(furan-2-yl)-1-oxobut-3-en-2-yl)-1H-indole-2-carboxamide (5c).

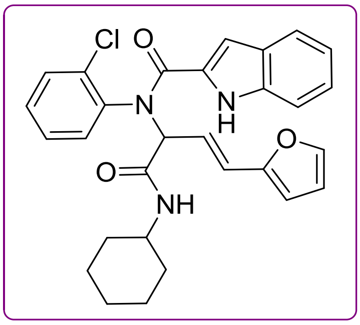

orange powder (**Yield:** 75%), **mp:** 100-102 ^oC.

HRMS (ESI) m/z: Calcd for $C_{29}H_{28}ClN_3O_3$ $[M+H]^+$ 502.1897, Found 502.1915.

^{1}H NMR (600 MHz, CDCl$_3$) δ ppm: 9.39 (s, 1H, NH), 7.83 (dd, *J* = 5.6 , 3.7 Hz, 1H), 7.52 (dd, *J* = 7.8 , 1.5 Hz, 1H), 7.49-7.42 (m, 2H), 7.40 (dd, *J* = 7.7, 1.7 Hz, 1H) , 7.35 (d, *J* = 8.6 Hz, 2H), 7.27 (d, *J* = 1.2 Hz, 1H), 7.22 (t, *J* = 8.0 Hz, 1H), 7.00 (t, *J* = 7.9 Hz, 1H), 6.54-6.45 (m, 1H), 6.31 (dd, *J* = 3.4, 1.8 Hz, 1H), 6.21 (d, *J* = 3.2 Hz, 1H), 5.99 (dd, *J* = 15.7, 9.3 Hz, 1H, =CH), 5.51 (d, *J* = 9.3 Hz, 1H, =CH), 5.25-5.15 (m, 1H), 3.87-3.77 (m , 1H), 2.00-1.85 (m, 2H), 1.73-1.65 (m, 2H), 1.60-1.55 (m, 1H), 1.38-1.30 (m, 2H), 1.24-1.11 (m, 3H).

^{13}C NMR (150 MHz, CDCl$_3$) δ ppm: 168.4, 162.3, 151.5, 142.6, 137.7, 135.5, 135.0, 132.5, 130.6, 130.5, 128.3, 127.7, 124.9, 124.5, 122.4, 121.5, 120.3, 119.3, 111.5, 111.3, 109.4, 106.6, 64.7, 48.7, 32.9, 25.5, 24.7.

(E)-N-(1-(butylamino)-1-oxo-4-phenylbut-3-en-2-yl)-N-(2-chlorophenyl)-1H-indole-2-carboxamide (5d).

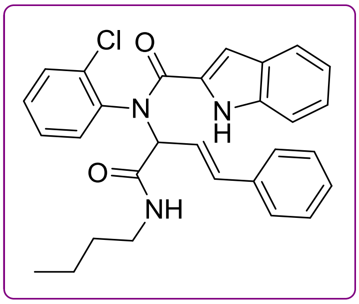

pale yellow powder (**Yield:** 80%), **mp:** 185 ^oC.

HRMS (ESI) m/z: Calcd for $C_{29}H_{28}ClN_3O_2$ $[M+H]^+$ 486.1948, Found 486.1961.

^{1}H NMR (600 MHz, CDCl$_3$) δ ppm: 9.39 (s, 1H, NH), 7.90 (d, *J* = 6.2 Hz, 1H), 7.52 (dd, *J* = 8.0, 1.3 Hz, 1H), 7.46-7.41 (m, 3H), 7.36 (d, *J* = 8.2 Hz, 2H), 7.33 (d, *J* = 7.3 Hz, 1H), 7.25-7.20 (m, 3H), 7.16 (d, *J* = 6.9 Hz, 1H), 7.01 (t, *J* = 7.7 Hz, 1H), 6.73 (d, *J* = 16.9 Hz, 1H), 6.46 (s, 1H), 6.02 (dd, *J* = 16.0, 9.5 Hz, 1H), 5.65 (d, *J* = 8.8 Hz, 1H), 5.28-5.22 (m, 1H), 3.37-3.28 (m, 2H), 1.56-1.48 (m, 2H), 1.39-1.30 (m, 2H), 0.93-0.86 (m, 3H).

^{13}C NMR (150 MHz, CDCl$_3$) δ ppm: 169.6, 162.3, 137.6, 137.2, 136.0, 135.5, 135.1, 132.7, 130.6, 130.4, 129.1, 128.5, 128.4, 128.3, 127.8, 126.7, 124.9, 122.4, 121.0, 120.3, 111.5, 106.6, 64.4, 39.6, 31.5, 20.0, 13.7.

(E)-N-(1-(tert-butylamino)-1-oxo-4-phenylbut-3-en-2-yl)-N-(2-chlorophenyl)-1H-indole-2-carboxamide (5e).

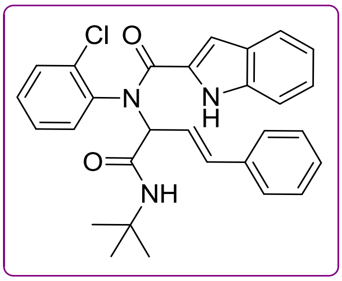

yellow powder (**Yield:** 80%), **mp:** 95-96 °C.

HRMS (ESI) m/z: Calcd for $C_{29}H_{28}ClN_3O_2$ $[M+H]^+$ 486.1948, Found 486.1968.

^{1}H NMR (600 MHz, CDCl$_3$) δ ppm: 9.43 (s, 1H, NH), 7.88-7.84 (m, 1H), 7.53-7.50 (m, 1H), 7.48-7.42 (m, 3H), 7.42-7.39 (m, 1H), 7.36 (dd, *J* = 8.2, 4.3 Hz, 2H), 7.35-7.32 (m, 1H), 7.28 (d, *J* = 7.3 Hz, 1H), 7.25-7.21 (m, 2H), 7.18 (d, J = 7.5 Hz, 1H) , 7.01 (t, *J* = 7.8 Hz, 1H), 6.69 (s, 1H), 6.03 (dd, *J* = 15.4, 9.2 Hz, 1H, =CH), 5.52 (d, *J* = 9.5 Hz, 1H, =CH), 5.26-5.22 (m, 1H), 1.38 (s, 9H).

^{13}C NMR (150 MHz, CDCl$_3$) δ ppm: 168.8, 162.2, 137.7, 137.0, 136.1, 135.5, 135.1, 132.6, 130.6, 130.4, 129.1, 128.6, 128.5, 128.2, 127.8, 126.7, 124.9, 122.4, 121.2, 120.3, 111.5, 106.5, 64.9, 51.6, 28.7.

(E)-N-(2-chlorophenyl)-N-(1-(isopropylamino)-1-oxo-4-phenylbut-3-en-2-yl)-1H-indole-2-carboxamide (5f).

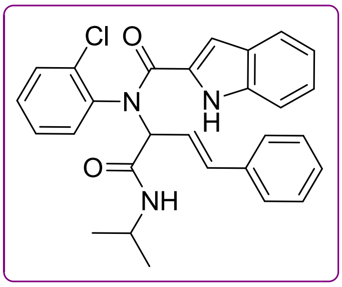

yellow powder (**Yield:** 72%), **mp:** 96-98 °C.

HRMS (ESI) m/z: Calcd for $C_{28}H_{26}ClN_3O_2$ $[M+H]^+$ 472.1792, Found 472.1816.

^{1}H NMR (600 MHz, CDCl$_3$) δ ppm: 9.37 (s, 1H, NH), 7.72 (d, *J* = 6.9 , 1H), 7.33 (d, *J* = 7.7, 1H), 7.25-7.20 (m, 2H), 7.18 (d, *J* = 8.5 Hz, 2H), 7.14 (d, *J* = 7.7 Hz, 1H), 7.11 (d, *J* = 7.3 Hz, 1H), 7.07 (d, *J* = 4.8 Hz, 1H), 7.06-7.03 (m, 1H), 7.03-7.00 (m, 1H), 6.98 (d, *J* = 7.5 Hz, 1H), 6.83 (t, *J* = 7.7 Hz, 1H), 6.53 (t, *J* = 16.0 Hz, 1H), 6.14 (d, *J* = 6.5 Hz, 1H), 5.85 (dd, *J* = 16.0, 9.2 Hz, 1H, =CH), 5.44 (d, *J* = 9.2 Hz, 1H, =CH), 5.12-5.02 (m, 1H), 3.99-3.90 (m, 1H), 1.03-0.94 (m, 6H).

^{13}C NMR (150 MHz, CDCl$_3$) δ ppm: 168.8, 162.3, 137.7, 137.1, 136.0, 135.6, 135.1, 132.7, 130.6, 130.4, 129.1, 128.5, 128.4, 128.3, 127.8, 126.7, 124.9, 122.4, 121.1, 120.3, 111.6, 106.6, 64.5, 41.9, 22.6.

(E)-N-(2-chlorophenyl)-N-(1-(cyclohexylamino)-4-(2-nitrophenyl)-1-oxobut-3-en-2-yl)-1H-indole-2-carboxamide (5g).

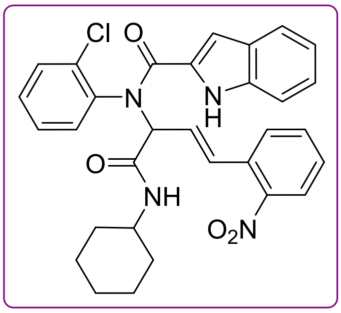

pale yellow powder (**Yield:** 75%), **mp:** 178-179 °C.

HRMS (ESI) m/z: Calcd for $C_{31}H_{29}ClN_4O_4$ $[M+H]^+$ 557.1956, Found 557.1922.

^{1}H NMR (600 MHz, $CDCl_3$) δ ppm: 9.49 (s, 1H, NH), 7.92 (d, *J* = 8.0 Hz, 1H), 7.88 (d, *J* = 7.7 Hz, 1H), 7.52-7.46 (m, 1H), 7.45 (d, *J* = 7.4 Hz, 1H), 7.43-7.37 (m, 2H), 7.37-7.27 (m, 4H), 7.16 (t, *J* = 8.0 Hz, 1H), 7.14-7.09 (m, 1H), 6.91 (t, *J* = 7.6 Hz, 1H), 6.55 (d, *J* = 7.1 Hz, 1H), 5.90 (dd, *J* = 15.8, 9.2 Hz, 1H, =CH), 5.64 (d, *J* = 9.1 Hz, 1H, =CH), 5.22-5.13 (m, 1H), 3.80-3.67 (m, 1H), 1.91-1.76 (m , 2H), 1.65-1.52 (m , 2H), 1.52-1.41 (m, 1H), 1.30-1.19 (m, 2H), 1.18-1.13 (m, 1H), 1.13-0.98 (m, 2H).

^{13}C NMR (150 MHz, $CDCl_3$) δ ppm: 168.0, 162.3, 147.4, 137.9, 135.6, 134.9, 133.6, 133.1, 132.8, 132.2, 130.8, 130.4, 129.4, 129.1, 128.9, 128.6, 127.7, 126.7, 124.9, 124.6, 122.3, 120.3, 111.7, 106.6, 64.2, 48.9, 32.7, 25.4, 24.8.

(E)-N-(2-chlorophenyl)-N-(1-(cyclopentylamino)-1-oxo-4-phenylbut-3-en-2-yl)-1H-indole-2-carboxamide (5h).

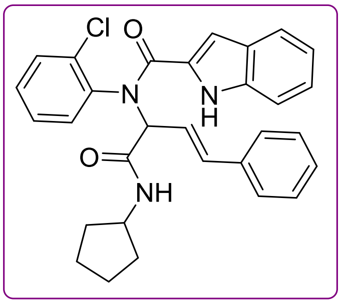

white powder (**Yield:** 80%), **mp:** 186-188 °C.

HRMS (ESI) m/z: Calcd for $C_{30}H_{28}ClN_3O_2$ $[M+H]^+$ 498.1948, Found 498.1972.

^{1}H NMR (600 MHz, $CDCl_3$) δ ppm: 9.31 (s, 1H, NH), 7.71 (d, *J* = 6.6 Hz, 1H), 7.33 (dd, *J* = 7.9 Hz, 1.5 Hz, 1H), 7.30-7.26 (m , 1H), 7.26-7.21 (m, 2H), 7.21-7.17 (m, 2H), 7.16-7.12 (t, *J* = 7.5 Hz, 1H), 7.06-7.01 (m , 3H), 6.98 (dd, *J* = 7.8 , 1.7 Hz, 1H), 6.83 (t, *J* = 7.5 Hz, 1H), 6.56-6.51 (m, 1H), 6.32 (d, *J* = 7.1 Hz, 1H), 5.86 (dd, *J* = 16.0, 9.1 Hz, 1H, =CH), 5.44 (d, *J* = 8.8 Hz, 1H, =CH), 5.10-5.05 (m, 1H), 4.11-4.02 (m, 1H), 1.86-1.75 (m, 2H), 1.50-1.42 (m, 2H), 1.42-1.34 (m, 2H), 1.32-1.17 (m, 2H).

^{13}C NMR (150 MHz, $CDCl_3$) δ ppm: 169.2, 162.3, 137.7, 137.1, 136.0, 135.5, 135.1, 132.7, 130.6, 130.4, 129.1, 128.5,

128.3, 127.8, 126.7, 124.9, 123.2, 122.4, 121.1, 120.3, 111.6, 106.6, 64.5, 51.6, 33.0, 23.8.

(E)-N-(1-(tert-butylamino)-4-(4-methoxyphenyl)-1-oxobut-3-en-2-yl)-N-(2-chlorophenyl)-1H-indole-2-carboxamide (5i).

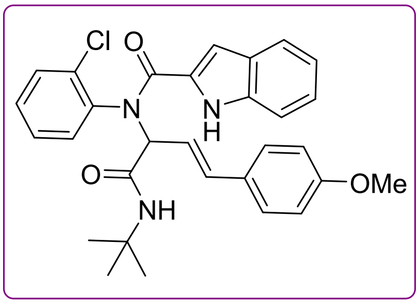

pale yellow powder (**Yield:** 80%), **mp:** 118-120 ^{o}C.

HRMS (ESI) m/z: Calcd for $C_{30}H_{30}ClN_3O_3$ $[M+H]^+$ 516.2054, Found 516.2071.

^{1}H NMR (600 MHz, CDCl$_3$) δ ppm: 9.37 (s, 1H, NH), 7.76 (dt, *J* = 6.8 , 1.9 Hz, 1H), 7.40 (dd, *J* = 7.7, 1.8 Hz, 1H), 7.36-7.34 (m, 1H), 7.34-7.33 (m, 1H), 7.33-7.29 (m, 1H), 7.29-7.27 (m, 1H), 7.25-7.22 (m, 1H), 7.11 (td, *J* = 7.7, 1.2 Hz, 1H), 7.02-6.99 (m, 1H), 6.90 (td, *J* = 7.5, 0.8 Hz, 1H), 6.76-6.66 (m, 2H), 6.56-6.51 (m, 1H), 6.26-6.19 (m, 1H), 5.77 (dd, *J* = 15.7, 9.2 Hz, 1H), 5.42 (d, *J* = 9.3 Hz, 1H), 5.17-5.11 (m, 1H), 3.66 (s, 3H, OMe), 1.28 (s, 9H, t-Bu).

^{13}C NMR (150 MHz, CDCl$_3$) δ ppm: 169.0, 162.1, 159.7, 137.7, 136.5, 135.5, 135.1, 132.6, 130.5, 130.4, 129.2, 128.9, 128.2, 128.0, 127.8, 124.8, 122.4, 120.3, 118.8, 113.9, 111.6, 106.5, 65.1, 55.2, 51.6, 28.7.

(E)-N-(1-(benzylamino)-4-(4-methoxyphenyl)-1-oxobut-3-en-2-yl)-N-(2-chlorophenyl)-1H-indole-2-carboxamide (5j).

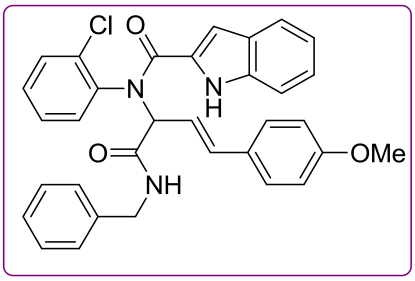

orange powder (**Yield:** 70%), **mp:** 98-100 ^{o}C.

HRMS (ESI) m/z: Calcd for $C_{33}H_{28}ClN_3O_3$ $[M+H]^+$ 550.1897, Found 550.1921

^{1}H NMR (600 MHz, CDCl$_3$) δ ppm: 9.39 (s, 1H, NH), 7.89-7.86 (m, 1H), 7.51 (dd, *J* = 7.8, 1.5 Hz, 1H), 7.45-7.43 (m, 2H), 7.42-7.39 (m, 1H), 7.37-7.32 (m, 4H), 7.31-7.29 (m, 2H), 7.25-7.21 (m, 2H), 7.10-7.07 (m, 1H), 7.03-6.99 (m, 1H), 6.86-6.83 (m, 1H), 6.77-6.75 (m, 1H), 6.73-6.66 (m, 1H), 6.68-6.64 (m, 1H), 5.86 (dd, *J* = 15.7, 9.5 Hz, 1H, =CH), 5.67 (d, *J* = 9.5 Hz, 1H, =CH), 5.27-5.23 (m, 1H), 4.60-4.46 (m, 2H), 3.77 (s, 3H).

^{13}C NMR (150 MHz, CDCl$_3$) δ ppm: 169.9, 162.3, 159.8, 139.9, 138.1, 137.0, 135.5, 135.2, 132.7, 130.6, 130.4, 129.0, 128.7, 128.3, 128.2, 128.0, 127.8, 127.6, 127.4, 124.9, 122.4, 120.3, 118.3, 113.9, 111.6, 106.6, 64.6, 55.2, 43.7.

(E)-N-(1-(butylamino)-4-(furan-2-yl)-1-oxobut-3-en-2-yl)-N-(2-chlorophenyl)-1H-indole-2-carboxamide (5k).

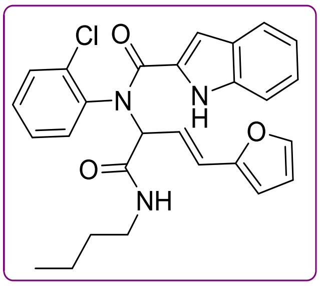

orange powder (**Yield:** 78%), **mp:** 86-88 °C.

HRMS (ESI) m/z: Calcd for $C_{27}H_{26}ClN_3O_3$ $[M+H]^+$ 476.1741, Found 476.1698.

^{1}H NMR (600 MHz, CDCl$_3$) δ ppm: 9.32 (s, 1H, NH), 7.85-7.81 (m, 1H), 7.52 (dd, *J* = 8.0, 1.5 Hz, 1H), 7.49-7.42 (m, 2H), 7.42-7.37 (m, 1H), 7.35 (d, *J* = 8.6 Hz, 2H), 7.27 (d, *J* = 1.5 Hz, 1H), 7.24-7.19 (m, 1H), 7.03-6.98 (m, 1H), 6.51-6.47 (m, 1H), 6.40-6.37 (m , 1H), 6.21 (d, *J* = 3.2 Hz, 1H), 5.98 (dd, *J* = 15.6, 9.3 Hz, 1H, =CH), 5.53 (d, *J* = 9.5 Hz, 1H, =CH), 5.25-5.17 (m, 1H), 3.38-3.25 (m, 2H), 1.58-1.47 (m, 2H), 1.40-1.29 (m, 2H), 0.97-0.85 (m, 3H).

^{13}C NMR (150 MHz, CDCl$_3$) δ ppm: 169.3, 162.2, 151.5, 142.7, 137.7, 135.4, 135.0, 132.5, 130.6, 130.5, 129.0, 128.3, 127.8, 124.9, 122.4, 121.4, 120.3, 119.2, 111.5, 111.3, 109.5, 106.6, 64.6, 39.6, 31.5, 20.0, 13.7.

(E)-N-(1-(cyclohexylamino)-4-(4-methoxyphenyl)-1-oxobut-3-en-2-yl)-N-(2,4-dichlorophenyl)-1H-indole-2-carboxamide (5l).

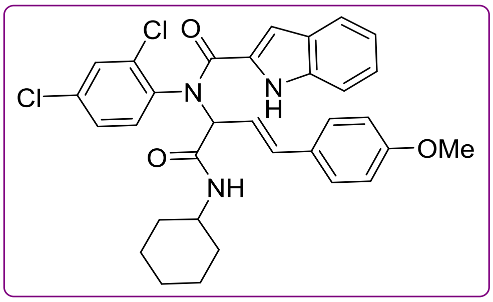

yellow powder (**Yield:** 76%), **mp:** 124-126 °C.

HRMS (ESI) m/z: Calcd for $C_{32}H_{31}Cl_2N_3O_3$ $[M+H]^+$ 576.1821, Found 576.1798.

^{1}H NMR (600 MHz, CDCl$_3$) δ ppm: 9.49 (s, 1H, NH), 7.90 (d, *J* = 8.4 Hz, 1H), 7.45 (d, *J* = 2.2 Hz, 1H), 7.44-7.41 (m, 2H), 7.38-7.35 (m, 2H), 7.24-7.21 (m, 1H), 7.14-7.11 (m, 1H), 7.05-7.01 (m, 1H), 6.89-6.85 (m, 1H), 6.82-6.78 (m, 2H), 6.66-6.63 (m, 1H), 6.21 (d, *J* = 7.7 Hz, 1H), 5.80 (dd, *J* = 16.0, 9.2 Hz, 1H, =CH), 5.63 (d, *J* = 9.2 Hz, 1H, =CH), 5.36-5.32 (m, 1H), 3.78 (s, 4H), 2.00-1.85 (m, 3H), 1.72-1.64 (m, 3H), 1.61-1.55 (m, 1H), 1.38-1.29 (m, 3H).

^{13}C NMR (150 MHz, CDCl$_3$) δ ppm: 168.8, 162.1, 159.9, 137.2, 136.1, 135.7, 135.6, 135.3, 133.6, 130.2, 128.9, 128.6, 128.5, 128.0, 127.7, 125.0, 122.4, 120.4, 118.1, 114.0, 111.6, 106.5, 64.3, 55.3, 48.8, 32.9, 25.5, 24.8.

(E)-N-(1-(butylamino)-1-oxo-4-phenylbut-3-en-2-yl)-N-(2,4-dichlorophenyl)-1H-indole-2-carboxamide (5m).

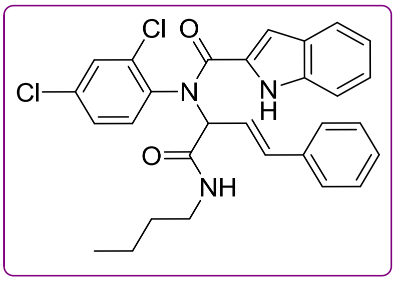

light brown powder (**Yield:** 70%), **mp:** 188-190 °C.

HRMS (ESI) m/z: Calcd for $C_{29}H_{27}Cl_2N_3O_2$ $[M+H]^+$ 520.1559, Found 520.1508.

^{1}H NMR (600 MHz, CDCl$_3$) δ ppm: 11.69 (s, 1H, NH), 8.27 (t, *J* = 5.6, Hz, 1H), 8.09 (d, *J* = 8.5 Hz, 1H), 7.75 (d, *J* = 2.4 Hz, 1H), 7.68 (dd, *J* = 8.6, 2.2 Hz, 1H), 7.55-7.31 (m, 3H), 7.30-7.26 (m, 1H), 7.26-7.20 (m, 1H), 7.19-7.13 (m, 2H), 7.13-6.96 (m, 1H), 6.96-6.91 (m, 1H), 6.75-6.64 (m, 1H), 5.78-5.71 (m, 1H), 5.70-5.65 (m, 1H), 5.33-5.17 (m, 1H) , 3.17-3.06 (m , 2H), 1.46-1.36 (m, 2H), 1.35-1.22 (m, 2H), 0.86 (t, *J* = 7.4 Hz, 3H).

^{13}C NMR (150 MHz, CDCl$_3$) δ ppm: 169.2, 160.9, 136.8, 136.1, 135.8, 135.7, 134.4, 134.2, 129.6, 128.8, 128.6, 128.5, 128.3, 126.8, 126.3, 124.0, 121.8, 121.5, 119.8, 112.2, 104.6, 63.2, 38.5, 31.2, 19.5, 13.7.

(E)-N-(2-chloro-4-methylphenyl)-N-(1-(cyclohexylamino)-1-oxo-4-phenylbut-3-en-2-yl)-1H-indole-2-carboxamide (5n).

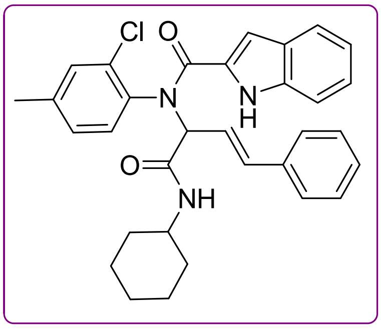

white powder (**Yield:** 77%), **mp:** 134-136 °C.

HRMS (ESI) m/z: Calcd for $C_{32}H_{32}ClN_3O_2$ $[M+H]^+$ 526.2261, Found 526.2184.

^{1}H NMR (600 MHz, CDCl$_3$) δ ppm: 9.33 (s, 1H, NH), 7.59 (d, *J* = 7.8 Hz, 1H), 7.34-7.25 (m, 3H), 7.23 (t, *J* = 7.1 Hz, 1H), 7.20-7.07 (m, 6H), 6.92 (t, *J* = 7.5 Hz, 1H), 6.63-6.55 (m, 1H), 6.29-6.22 (m, 1H), 5.97 (dd, *J* = 15.9, 9.2 Hz, 1H, =CH), 5.47 (d, *J* = 9.2 Hz, 1H, =CH), 5.24-5.16 (m, 1H), 3.79-3.67 (m, 1H), 2.34 (s, 3H), 1.91-1.76 (m, 2H), 1.63-1.54 (m, 2H), 1. 51-1.44 (m, 1H), 1.31-1.20 (m, 2H), 1.14-1.00 (m, 3H).

^{13}C NMR (151 MHz, CDCl$_3$) δ ppm: 168.7, 162.4, 141.1, 136.9, 136.2, 135.5, 134.9, 134.4, 132.0, 130.9, 129.2, 129.0, 128.5, 128.2, 127.8, 126.8, 124.8, 122.4, 121.2, 120.3, 111.6, 106.5, 64.6, 48.6, 32.9, 25.5, 24.8, 21.2.

(E)-N-(2-chloro-4-methylphenyl)-N-(1-(cyclohexylamino)-4-(4-methoxyphenyl)-1-oxobut-3-en-2-yl)-1H-indole-2-carboxamide (5o).

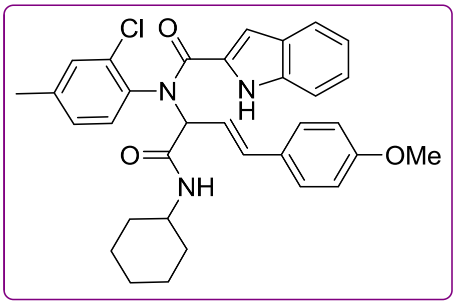

pale yellow powder (**Yield:** 80%), **mp:** 110 °C.

HRMS (ESI) m/z: Calcd for $C_{33}H_{34}ClN_3O_3$ $[M+H]^+$ 556.2367, Found 556.2298.

^{1}H NMR (600 MHz, CDCl$_3$) δ ppm: 9.35 (s, 1H, NH), 7.68 (d, *J* = 8.2 Hz, 1H), 7.37-7.32 (m, 3H), 7.24-7.20 (m, 2H), 7.14 (d, *J* = 8.5 Hz, 2H), 7.00 (t, *J* = 7.8 Hz, 1H), 6.79 (d, *J* = 8.8 Hz, 2H), 6.64-6.56 (m, 1H), 6.32-6.27 (m, 1H), 5.90 (dd, *J* = 16.0, 9.5 Hz, 1H, =CH), 5.53 (d, *J* = 9.5 Hz, 1H, =CH), 5.28 (m, 1H), 3.81 (m, 1H), 3.78 (s, 3H) , 2.43 (s, 3H), 2.02-1.95 (m, 1H), 1.95-1.86 (m, 2H), 1.61-1.55 (m, 1H), 1.39-1.31 (m, 2H), 1.26-1.12 (m, 4H).

^{13}C NMR (150 MHz, CDCl$_3$) δ ppm: 168.9, 162.3, 159.7, 141.0, 136.5, 135.4, 134.9, 134.5, 132.0, 130.8, 129.2, 129.0, 128.9, 128.0, 127.8, 124.8, 122.4, 120.3, 118.8, 113.9, 111.5, 106.5, 64.7, 55.3, 48.6, 32.9, 25.5, 24.8, 21.2.

(E)-N-(1-(cyclohexylamino)-1-oxo-4-phenylbut-3-en-2-yl)-N-(p-tolyl)-1H-indole-2-carboxamide (5p).

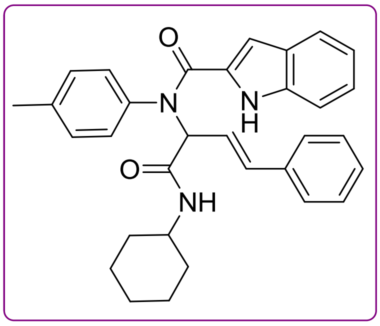

pale yellow powder (**Yield:** 77%), **mp:** 172-174 °C.

HRMS (ESI) m/z: Calcd for $C_{32}H_{33}N_3O_2$ $[M+H]^+$ 492.2651, Found 492.2672. .

^{1}H NMR (300 MHz, CDCl$_3$) δ ppm: 9.57 (s, 1H), 7.66-7.10 (m, 12H), 7.09-6.96 (m, 1H), 6.74 (d, *J* = 15.9 Hz, 1H), 6.54-6.41 (m, 1H), 6.41-6.30 (m, 1H), 5.54 (d, *J* = 8.3 Hz, 1H), 5.35-5.19 (m, 1H), 3.96-3.76 (m, 1H), 2.60-2.21 (m, 3H), 2.04-1.85 (m, 2H), 1.79-1.64 (m, 2H), 1.49-1.27 (m, 3H), 1.26-1.05 (m, 3H).

^{13}C NMR (75 MHz, CDCl$_3$) δ ppm: 168.3, 162.2, 139.3, 137.8, 136.7, 136.0, 135.4, 130.4, 130.0, 129.1, 128.7, 128.4, 127.7, 126.9, 124.8, 122.7, 122.4, 120.2, 111.6, 107.8, 65.4, 48.6, 33.0, 25.5, 24.8, 21.4.

(E)-N-(2-chlorophenyl)-N-(1-(2,6-dimethylphenyl)amino)-1-oxo-4-phenylbut-3-en-2-yl)-1H-indole-2-carboxamide (5q).

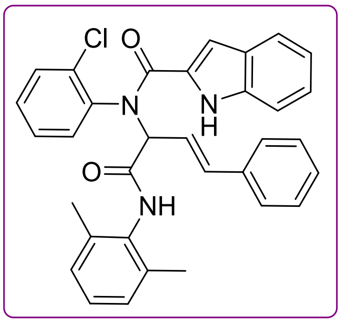

light brown powder (**Yield:** 90%), **mp:** 105 °C.

HRMS (ESI) m/z: Calcd for $C_{33}H_{28}ClN_3O_2$ $[M+H]^+$ 534.1948, Found 534.1943.

^{1}H NMR (600 MHz, $CDCl_3$) δ ppm: 9.52 (s, 1H, NH), 7.98-7.93 (m, 1H), 7.57 (dd, *J* = 8.2, 1.2 Hz, 1H), 7.41-7.33 (m, 4H), 7.33-7.30 (m, 1H), 7.25-7.21 (m, 2H), 7.20-7.17 (m, 2H), 7.12-6.98 (m, 6H), 6.87-6.82 (m, 1H), 6.10 (dd, *J* = 15.7, 9.5 Hz, 1H, =CH), 5.90 (d, *J* = 9.2 Hz, 1H, =CH), 5.33-5.27 (m, 1H), 2.26 (s, 6H).

^{13}C NMR (150 MHz, $CDCl_3$) δ ppm: 168.4, 162.5, 137.6, 137.0, 136.0, 135.6, 135.5, 135.2, 133.6, 132.9, 130.7, 130.4, 129.0, 128.8, 128.6, 128.3, 128.2, 127.8, 127.3, 126.8, 125.0, 122.4, 120.7, 120.4, 111.6, 106.8, 64.5, 18.5.

(E)-2-(N-(2-chlorophenyl)-2-(1H-indol-3-yl)acetamido)-N-cyclohexyl-4-phenylbut-3-enamide (5r).

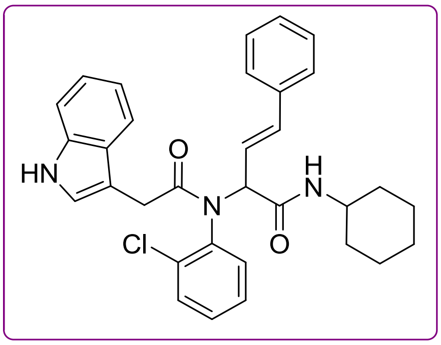

white powder (**Yield:** 73%), **mp:** 175 °C.

HRMS (ESI) m/z: Calcd for $C_{32}H_{32}ClN_3O_2$ $[M+H]^+$ 526.2261, Found 526.2334.

^{1}H NMR (600 MHz, $CDCl_3$) δ ppm: 8.16 (s, 1H, NH), 7.54 (d, *J* = 7.2 Hz, 1H), 7.45-7.41 (m, 2H), 7.36-7.33 (m, 1H), 7.33-7.27 (m, 3H), 7.26-7.19 (m, 3H), 7.16-7.11 (m, 2H), 7.06-7.01 (m, 1H), 6.90-6.87 (m, 1H), 6.69-6.62 (m, 1H), 6.32-6.21 (m, 1H), 5.88 (dd, *J* = 15.5, 9.2 Hz, 1H, =CH), 5.51 (d, *J* = 9.2 Hz, 1H, =CH), 3.80-3.68 (m, 1H), 3.63-3.47 (m, 2H), 1.94-1.69 (m, 3H), 1.69-1.51 (m, 3H), 1.37-1.22 (m, 2H), 1.17-1.00 (m, 2H).

^{13}C NMR (150 MHz, $CDCl_3$) δ ppm: 172.0, 169.1, 136.8, 136.2, 136.1, 134.5, 132.0, 130.1, 130.0, 128.5, 128.1, 127.9, 127.3, 126.7, 123.5, 123.2, 121.9, 121.3, 119.4, 118.9, 111.1, 108.8, 62.9, 48.5, 32.7, 31.7, 25.4, 24.7.

(E)-N-benzyl-N-(1-(cyclohexylamino)-1-oxo-4-phenylbut-3-en-2-yl)-1H-indole-2-carboxamide (5s).

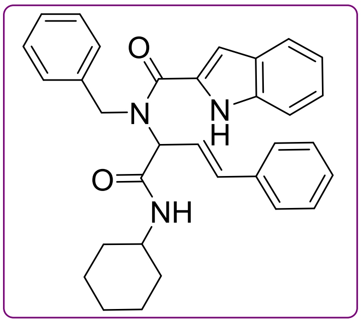

white powder (**Yield:** 75%), **mp:** 177 - 179 °C.

HRMS (ESI) m/z: Calcd for $C_{32}H_{33}N_3O_2$ [M+H]$^+$ 492.2651, Found 492.2639.   .

^{1}H NMR (600 MHz, CDCl$_3$) δ ppm: 9.52 (s, 1H, NH), 7.56-7.48 (m, 1H), 7.41-7.31 (m, 7H), 7.25-7.21 (m, 3H), 7.15-7.06 (m, 2H), 6.86-6.60 (m, 2H), 5.24-4.68 (m, 3H), 4.01 (dd, *J* = 9.8, 3.7 Hz, 1H), 3.71-3.64 (m, 1H), 2.50-2.40 (m, 1H), 2.16-2.09 (m, 1H), 1.88-1.69 (m, 3H), 1.67-1.51 (m, 4H), 1.37-1.23 (m, 3H).

^{13}C NMR (150 MHz, CDCl$_3$) δ ppm: 169.3, 162.8, 140.9, 139.1, 136.6, 135.8, 128.9, 128.5, 127.9, 127.8, 126.4, 122.3, 120.6, 111.7, 107.7, 106.0, 56.6, 48.4, 38.2, 32.8, 25.5, 24.6.

4-benzyl-2-(2-chlorophenyl)-N-cyclohexyl-1-oxo-1,2,3,4-tetrahydropyrazino[1,2-a]indole-3-carboxamide (6a).

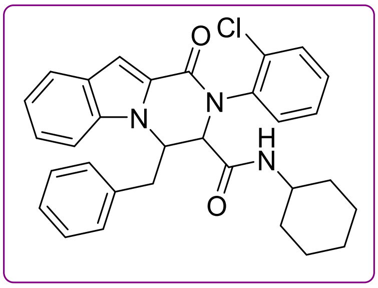

white powder, **mp:** 235-237 °C.

(**Yield:** 75%, **dr:** 66:34).

HRMS (ESI) m/z: Calcd for $C_{31}H_{30}ClN_3O_2$ [M+H]$^+$ 512.2105, Found 512.2132.

^{1}H NMR (600 MHz, CDCl$_3$) δ ppm: 7.70-7.65 (m, 1H), 7.61-7.54 (m, 1H), 7.48-7.43 (m, 1H), 7.42-7.36 (m, 1H), 7.36-7.30 (m, 2H), 7.22-7.17 (m, 1H), 7.17-7.12 (m, 3H), 7.12-7.08 (m, 1H), 7.07-7.00 (m, 2H), 6.98 (d, *J* = 6.3 Hz, 2H), 5.97-5.84 (m, 1H), 5.22-5.14 (m, 1H), 4.43-4.35 (m, 1H), 3.69-3.59 (m, 1H), 3.46-3.37 (m, 1H), 1.82-1.73 (m, 1H), 1.61-1.55 (m, 1H), 1.50-1.42 (m, 1H), 1.42-1.29 (m, 3H), 1.24-1.16 (m, 1H), 1.15-0.97 (m, 3H).

^{13}C NMR (150 MHz, CDCl$_3$) δ: 168.2, 158.3, 138.3, 136.5, 132.2, 131.0, 130.9, 129.2, 129.0, 128.8, 128.2, 127.4, 127.2, 127.0, 126.5, 125.3, 122.8, 120.9, 109.8, 108.8, 67.0, 57.2, 48.7, 40.0, 32.2, 25.1, 24.3.

2-(2-chlorophenyl)-N-cyclohexyl-4-(4-methoxybenzyl)-1-oxo-1,2,3,4-tetrahydropyrazino-[1,2-a]indole-3-carboxamide (6b).

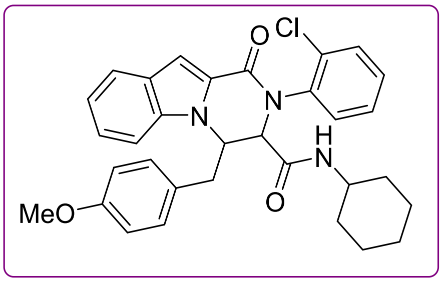

white crystal, **mp:** 248-250 °C.

(**Yield:** 85%, **dr:** 67:33).

HRMS (ESI) m/z: Calcd for $C_{32}H_{32}ClN_3O_3$ $[M+H]^+$ 542.2210, Found 542.2252.

^{1}H NMR (600 MHz, CDCl$_3$) δ: 7.68 (d, *J* = 7.7 Hz, 1H), 7.60-7.51 (m, 1H), 7.46-7.42 (m, 1H), 7.41-7.36 (m, 1H), 7.36-7.30 (m, 2H), 7.22 (t, *J* = 6.7 Hz, 1H), 7.11 (t, *J* = 7.5 Hz, 1H), 7.06-6.99 (m, 1H), 6.94 (d, *J* = 7.1 Hz, 1H), 6.90-6.84 (m, 1H), 6.71-6.63 (m, 2H), 5.95-5.85 (m, 1H), 5.16-5.06 (m, 1H), 4.44-4.31 (m, 1H), 3.71 (s, 3H), 3.68-3.58 (m, 1H), 3.39-3.19 (m, 2H), 1.83-1.66 (m, 2H), 1.62-1.54 (m, 1H), 1.51-1.42 (m, 1H), 1.41-1.27 (m, 2H), 1.15-0.97 (m, 3H), 0.79-0.64 (m, 1H).

^{13}C NMR (150 MHz, CDCl$_3$) δ: 168.3, 158.8, 158.3, 138.4, 136.7, 132.2, 131.0, 130.2, 129.3, 128.7, 128.2, 127.4, 127.0 126.5, 125.3, 122.8, 120.9, 114.3, 109.9, 108.8, 66.9, 57.4, 55.3, 48.7, 39.1, 32.2, 25.2, 24.3.

2-(2-chlorophenyl)-N-cyclohexyl-4-(furan-2-ylmethyl)-1-oxo-1,2,3,4-tetrahydropyrazino[1,2-a]indole-3-carboxamide (6c).

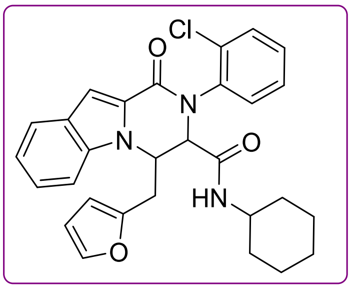

light brown powder, **mp:** 110-113 °C.

(**Yield:** 63%, **dr:** 66:33).

HRMS (ESI) m/z: Calcd for $C_{29}H_{28}ClN_3O_3$ $[M+H]^+$ 502.1897, Found 502.1908.

^{1}H NMR (600 MHz, CDCl$_3$) δ ppm: 7.69 (d, *J* = 8.0 Hz, 1H), 7.59-7.51 (m, 1H), 7.46-7.39 (m, 2H), 7.38-7.32 (m, 2H), 7.32-7.27 (m, 1H), 7.19-7.11 (m, 2H), 7.11-6.95 (m, 1H), 6.20-6.13 (m, 1H), 5.98-5.89 (m, 1H), 5.83 (d, *J* = 6.9 Hz, 1H), 5.33-5.22 (m, 1H), 4.48-4.35 (m, 1H), 3.60-3.43 (m, 2H), 3.39-3.27 (m, 1H), 1.84-1.73 (m, 2H), 1.64-1.53 (m, 2H), 1.52-1.44 (m, 1H), 1.44-1.37 (m, 1H), 1.19-0.96 (m, 4H).

^{13}C NMR (150 MHz, CDCl$_3$) δ ppm: 168.0, 158.4, 150.1, 142.2, 138.2, 136.6, 132.2, 130.9, 129.4, 128.7, 128.1, 127.1, 126.5, 125.5, 122.9, 121.1, 110.6, 109.6, 108.9, 108.6. 66.8, 54.5, 48.8, 32.5, 25.2, 24.5, 24.3.

4-benzyl-N-butyl-2-(2-chlorophenyl)-1-oxo-1,2,3,4-tetrahydropyrazino[1,2-a]indole-3-carboxamide (6d).

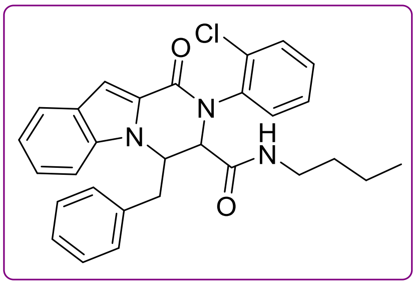

pale yellow powder, **mp:** 215-217 ^{o}C.

(**Yield:** 80%, **dr:** 70:30).

HRMS (ESI) m/z: Calcd for $C_{29}H_{28}ClN_3O_2$ $[M+H]^+$ 486.1948, Found 486.1978.

^{1}H NMR (600 MHz, CDCl$_3$) δ ppm: 7.69-7.61 (m, 1H), 7.60-7.52 (m, 1H), 7.41-7.31 (m, 3H), 7.31-7.27 (m, 1H), 7.23-7.17 (m, 1H), 7.17-7.12 (m, 3H), 7.12-7.08 (m, 1H), 7.07-7.01 (m, 2H), 7.01-6.94 (m, 1H), 6.44-6.30 (m, 1H), 5.27-5.13 (m, 1H), 4.47-4.35 (m, 1H), 3.46-3.26 (m, 2H), 3.24-2.95 (m, 2H), 1.24-1.12 (m, 2H), 0.95-0.81 (m, 2H), 0.64-0.52 (m, 3H).

^{13}C NMR (150 MHz, CDCl$_3$) δ ppm: 169.2, 158.3, 138.4, 136.7, 136.5, 132.1, 131.0, 129.2, 128.8, 128.2, 127.5, 127.2, 127.0, 126.4, 125.4, 122.8, 120.9, 110.0, 109.0, 67.1, 57.3, 40.0, 39.8, 31.2, 19.5, 13.4.

4-benzyl-N-(tert-butyl)-2-(2-chlorophenyl)-1-oxo-1,2,3,4-tetrahydropyrazino[1,2-a]indole-3-carboxamide (6e).

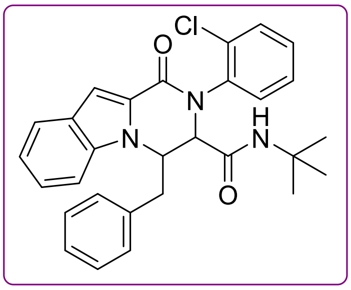

white powder, **mp:** 253-255 ^{o}C.

(**Yield:** 68%, **dr:** 61:39).

HRMS (ESI) m/z: Calcd for $C_{29}H_{28}ClN_3O_2$ $[M+H]^+$ 486.1948, Found 486.1982.

^{1}H NMR (600 MHz, CDCl$_3$) δ ppm: 7.69 (d, *J* = 7.7 Hz, 1H), 7.61-7.57 (m, 1H), 7.50-7.46 (m, 1H), 7.41-7.30 (m, 3H), 7.21 (t, *J* = 7.1 Hz, 1H), 7.18-7.13 (m, 3H), 7.11 (t, *J* = 7.8 Hz, 1H), 7.09-7.02 (m, 2H), 7.02-6.97 (m, 1H), 6.83-6.77 (m, 1H), 5.18-5.09 (m, 1H), 4.34-4.24 (m, 1H), 3.49-3.37 (m, 2H), 1.08 (s, 9H).

^{13}C NMR (150 MHz, CDCl$_3$) δ ppm: 168.5, 158.3, 138.3, 136.7, 136.5, 132.3, 131.1, 129.4, 128.9, 128.8, 128.3, 127.5, 127.2, 126.9, 126.5, 125.4, 122.8, 120.9, 109.8, 108.7, 67.5, 57.4, 52.1, 40.1, 28.4.

4-benzyl-2-(2-chlorophenyl)-N-isopropyl-1-oxo-1,2,3,4-tetrahydropyrazino[1,2-a]indole-3-carboxamide (6f).

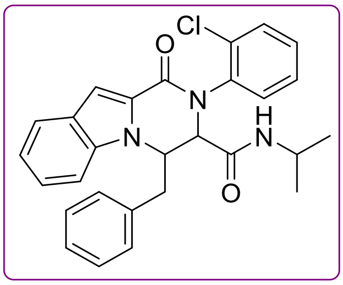

white crystal, **mp:** 280-282 °C.

(**Yield:** 45%, **dr:** 63:37).

HRMS (ESI) m/z: Calcd for $C_{28}H_{26}ClN_3O_2$ $[M+H]^+$ 472.1792, Found 472.1811.

^{1}H NMR (600 MHz, $CDCl_3$) δ ppm: 7.69 (d, *J* = 7.7 Hz, 1H), 7.60 (d, *J* = 7.3 Hz, 1H), 7.49-7.46 (m, 1H), 7.37-7.31 (m, 3H), 7.21 (t, *J* = 7.8 Hz, 1H), 7.18-7.13 (m, 3H), 7.11 (t, *J* = 7.8 Hz, 1H), 7.08-7.00 (m, 2H), 7.00-6.95 (m, 1H), 5.76 (d, *J* = 6.9 Hz, 1H), 5.22-5.14 (m, 1H), 4.41-4.34 (m, 1H), 3.99-3.89 (m, 1H), 3.42 (d, *J* = 7.1 Hz, 2H), 1.11-0.98 (m, 3H), 0.74-0.64 (m, 3H).

^{13}C NMR (150 MHz, $CDCl_3$) δ ppm: 168.4, 158.2, 138.3, 136.4, 132.1, 131.1, 129.3, 129.2, 128.95, 128.9, 128.8, 128.2, 127.3, 127.0, 126.4, 125.4, 122.8, 121.0, 109.9, 108.9, 67.0, 57.1, 42.2, 39.9, 22.1.

2-(2-chlorophenyl)-N-cyclohexyl-4-(2-nitrobenzyl)-1-oxo-1,2,3,4-tetrahydropyrazino[1,2-a]indole-3-carboxamide (6g).

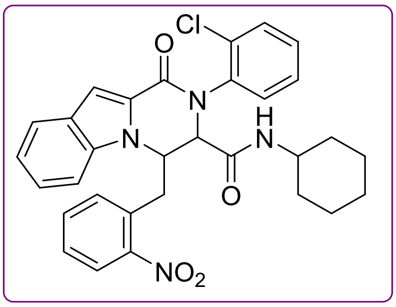

pale yellow powder, **mp:** 143-145 °C.

(**Yield:** 69%, **dr:** 79:21)

HRMS (ESI) m/z: Calcd for $C_{31}H_{29}ClN_4O_4$ $[M+H]^+$ 557.1956, Found 557.1983.

^{1}H NMR (600 MHz, $CDCl_3$) δ ppm: 7.93 (d, *J* = 7.7 Hz, 1H), 7.63-7.47 (m, 3H), 7.47-7.39 (m, 1H), 7.39-7.28 (m, 2H), 7.25-7.15 (m, 1H), 7.02-6.86 (m, 3H), 6.72-6.64 (m, 1H), 6.49 (d, *J* = 7.0 Hz, 1H), 6.06-5.93 (m, 1H), 5.60-5.48 (m, 1H), 4.57-4.45 (m, 1H), 3.90-3.73 (m, 1H), 3.71-3.56 (m, 2H), 1.93-1.70 (m, 2H), 1.61-1.53 (m, 1H), 1.34-1.24 (m, 1H), 1.24-1.11 (m, 2H), 1.11-0.98 (m, 2H), 0.92-0.77 (m, 2H).

^{13}C NMR (150 MHz, $CDCl_3$) δ ppm: 167.4, 158.8, 149.2, 138.1, 137.3, 133.1, 131.8, 130.8, 129.4 ,128.7, 128.6, 128.2, 126.6, 124.9, 122.6, 120.7, 109.6, 109.0, 67.6, 55.7, 48.8, 39.1, 32.4, 25.2, 24.5.

4-benzyl-2-(2-chlorophenyl)-N-cyclopentyl-1-oxo-1,2,3,4-tetrahydropyrazino[1,2-a]indole-3-carboxamide (6h).

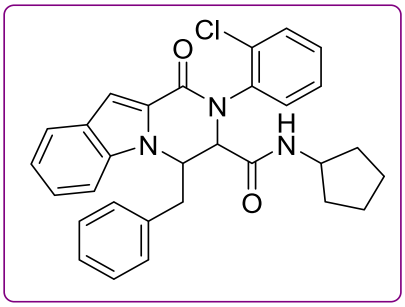

pale yellow powder, **mp:** 235-237 °C.

(**Yield:** 75%, **dr:** 66:34).

HRMS (ESI) m/z: Calcd for $C_{30}H_{28}ClN_3O_2$ $[M+H]^+$ 498.1948, Found 498.1978.

^{1}H NMR (600 MHz, CDCl$_3$) δ ppm: 7.69-7.61 (m, 1H), 7.56-7.47 (m, 1H), 7.47-7.36 (m, 2H), 7.36-7.27 (m, 2H), 7.18-7.02 (m, 5H), 7.02-6.92 (m, 2H), 6.90-6.82 (m, 1H), 6.40-6.19 (m, 1H), 5.26-5.09 (m, 1H), 4.50-4.31 (m, 1H), 4.13-4.02 (m, 1H), 3.52-3.22 (m, 2H), 1.85-1.59 (m, 2H), 1.58-1.41 (m, 3H), 1.41-1.28 (m, 3H).

^{13}C NMR (150 MHz, CDCl$_3$) δ ppm: 168.6, 158.8, 138.5, 136.9, 136.7, 132.4, 132.3, 130.9, 130.8, 129.4, 128.8, 128.2, 127.1, 126.9, 126.7, 125.2, 122.7, 120.8, 109.9, 108.6, 67.2, 57.6, 55.8, 32.7, 30.4, 23.6.

N-(tert-butyl)-2-(2-chlorophenyl)-4-(4-methoxybenzyl)-1-oxo-1,2,3,4-tetrahydropyrazino[1,2-a]indole-3-carboxamide (6i).

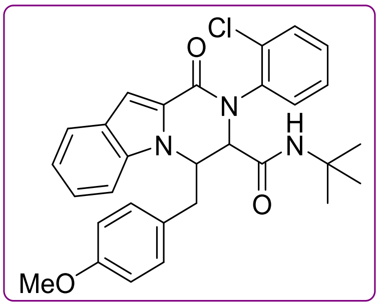

pale yellow powder, **mp:** 237-239 °C.

(**Yield:** 78%, **dr:** 65:35).

HRMS (ESI) m/z: Calcd for $C_{30}H_{30}ClN_3O_3$ $[M+H]^+$ 516.2054, Found 516.2082.

^{1}H NMR (600 MHz, CDCl$_3$) δ ppm: 7.69 (d, *J* = 7.7 Hz, 1H), 7.60-7.56 (m, 1H), 7.48-7.45 (m, 1H), 7.40-7.31 (m, 3H), 7.22 (t, *J* = 7.2 Hz, 1H), 7.11 (t, *J* = 7.2 Hz, 1H), 6.95 (d, *J* = 7.8 Hz, 2H), 6.89 (d, *J* = 6.9 Hz, 1H), 6.72-6.66 (m, 2H), 5.72-5.66 (m, 1H), 5.10-5.03 (m, 1H), 4.31-4.25 (m, 1H), 3.71 (m, 3H), 3.35 (d, *J* = 7.2 Hz, 2H), 1.08 (s, 9H).

^{13}C NMR (150 MHz, CDCl$_3$) δ ppm: 168.6, 158.8, 158.2, 138.4, 136.7, 132.2, 131.0, 130.1, 129.3, 128.5, 128.2, 127.4, 127.0, 126.5, 125.3, 122.8, 120.9, 114.3, 109.7, 108.6, 67.2, 57.4, 55.3, 52.1, 39.1, 28.3.

N-benzyl-2-(2-chlorophenyl)-4-(4-methoxybenzyl)-1-oxo-1,2,3,4-tetrahydropyrazino[1,2-a]indole-3-carboxamide (6j).

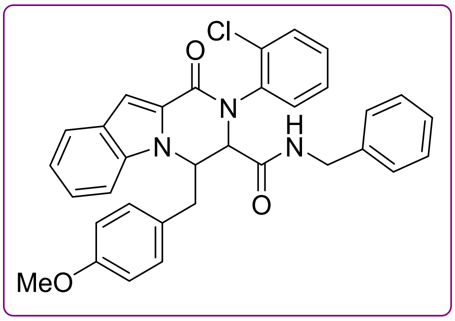

pale yellow powder, **mp:** 246 °C.

(**Yield:** 65%, **dr:** 69:31).

HRMS (ESI) m/z: Calcd for $C_{33}H_{28}ClN_3O_3$ $[M+H]^+$ 550.1897, Found 550.1904.

^{1}H NMR (600 MHz, CDCl$_3$) δ ppm: 7.68 (d, *J* = 8.0 Hz, 1H), 7.56 (d, *J* = 7.8 Hz, 1H), 7.43-7.37 (m, 1H), 7.37-7.32 (m, 1H), 7.32-7.30 (m, 1H), 7.30-7.27 (m, 1H), 7.22 (t, *J* = 7.3 Hz, 1H), 7.16 (t, *J* = 7.5 Hz, 1H), 7.11-7.00 (m, 2H), 6.95 (d, *J* = 7.9 Hz, 1H), 6.91 (t, *J* = 7.3 Hz, 1H), 6.89-6.86 (m, 1H), 6.86-6.80 (m, 1H), 6.72-6.64 (m, 4H), 6.57-6.52 (m, 1H), 5.20-5.14 (m, 1H), 4.52-4.47 (m, 1H), 4.47-4.43 (m, 1H), 4.28-4.22 (m, 1H), 3.72 (s, 3H), 3.41-3.23 (m, 2H).

^{13}C NMR (150 MHz, CDCl$_3$) δ ppm: 169.5, 158.8, 158.1, 138.2, 136.7, 132.0, 131.0, 130.2, 129.2, 128.4, 128.3, 128.2, 127.3, 126.9, 126.6, 125.5, 122.9, 121.1, 114.3, 110.0, 109.3, 67.0, 57.4, 55.3, 43.8, 39.0.

N-butyl-2-(2-chlorophenyl)-4-(furan-2-ylmethyl)-1-oxo-1,2,3,4-tetrahydropyrazino[1,2-a]indole-3-carboxamide (6k).

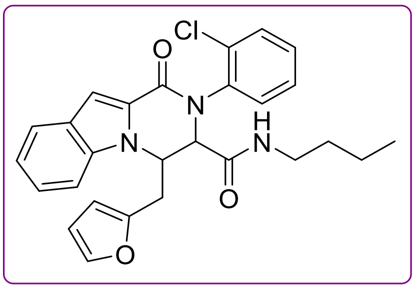

light brown powder, **mp:** 98-100 °C.

(**Yield:** 75%, **dr:** 66: 33).

HRMS (ESI) m/z: Calcd for $C_{27}H_{26}ClN_3O_3$ $[M+H]^+$ 476.1741, Found 476.1763.

^{1}H NMR (600 MHz, CDCl$_3$) δ ppm: 7.69 (d, *J* = 8.0 Hz, 1H), 7.55 (d, *J* = 8.9 Hz, 1H), 7.44-7.38 (m, 3H), 7.38-7.27 (m, 4H), 7.19 (d, *J* = 8.3 Hz, 1H), 7.14 (t, *J* = 7.6 Hz, 1H), 6.27-6.08 (m, 1H), 6.07-5.88 (m, 1H), 5.36-5.25 (m, 1H), 4.51-4.39 (m, 1H), 3.39-3.26 (m, 2H), 3.25-3.07 (m, 2H), 1.27-1.16 (m, 2H), 0.98-0.83 (m, 2H), 0.67-0.53 (m, 3H).

^{13}C NMR (150 MHz, CDCl$_3$) δ ppm: 168.9, 158.4, 150.1, 142.2, 138.2, 136.6, 132.1, 130.9, 129.4, 128.4, 128.1, 127.2, 126.4, 125.6, 122.9, 121.2, 110.6, 109.7, 109.1, 108.6, 66.9, 54.5, 39.7, 32.4, 31.2, 19.5, 13.4.

N-cyclohexyl-2-(2,4-dichlorophenyl)-4-(4-methoxybenzyl)-1-oxo-1,2,3,4-tetrahydropyrazino[1,2-a]indole-3-carboxamide (6l).

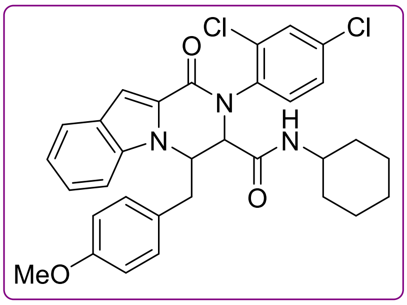

pale yellow powder, **mp:** 273-275 °C.

(**Yield:** 77%, **dr:** 71: 28).

HRMS (ESI) m/z: Calcd for $C_{32}H_{31}Cl_2N_3O_3$ $[M+H]^+$ 576.1821, Found 576.1833.

^{1}H NMR (600 MHz, $CDCl_3$) δ ppm: 7.68 (d, *J* = 7.3 Hz, 1H), 7.62-7.55 (m, 1H), 7.45 (s, br, 1H), 7.31 (s, br, 1H), 7.25-7.19 (m, 1H), 7.15-7.08 (m, 1H), 7.03 (d, *J* = 7.2 Hz, 1H), 6.95 (d, *J* = 6.7 Hz, 1H), 6.90-6.84 (m, 1H), 6.84-6.78 (m, 1H), 6.72 (d, *J* = 7.1 Hz, 1H), 6.70-6.63 (m, 1H), 5.75-5.68 (m, 1H), 5.11-5.04 (m, 1H), 4.36-4.29 (m, 1H), 3.73 (s, 3H), 3.67-3.58 (m, 1H), 3.36-3.29 (m, 2H), 1.80-1.72 (m, 1H), 1.71-1.61 (m, 2H), 1.60-1.53 (m, 1H), 1.50-1.42 (m, 1H), 1.42-1.32 (m, 2H), 1.16-1.08 (m, 1H), 1.08-0.96 (m, 2H).

^{13}C NMR (150 MHz, $CDCl_3$) δ ppm: 168.1, 158.9, 158.2, 137.0, 136.7, 134.5, 133.1, 130.8, 130.1, 128.9, 128.5, 128.3, 127.0, 126.2, 125.5, 122.9, 121.0, 114.4, 109.8, 109.0, 66.6, 57.3, 55.3, 48.8, 39.0, 32.2, 25.1, 24.2.

4-benzyl-N-butyl-2-(2,4-dichlorophenyl)-1-oxo-1,2,3,4-tetrahydropyrazino[1,2-a] indole-3-carboxamide (6m).

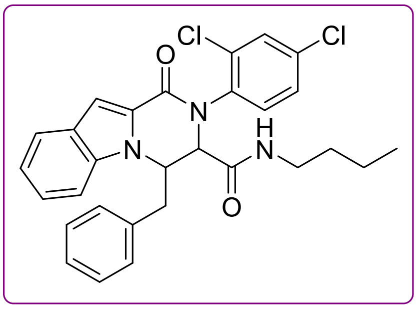

white powder, **mp:** 222-225 °C.

(**Yield:** 77%).

HRMS (ESI) m/z: Calcd for $C_{29}H_{27}Cl_2N_3O_2$ $[M+H]^+$ 520.1559, Found 520.1560.

^{1}H NMR (600 MHz, $CDCl_3$) δ ppm: 8.34 (t, *J* = 5.4 Hz, 1H), 7.85 (d, *J* = 2.1 Hz, 1H), 7.58-7.53 (m, 2H), 7.37 (d, *J* = 8.6 Hz, 1H), 7.16 (s, br, 1H), 7.13-7.07 (m, 3H), 6.99-6.95 (m, 2H), 6.95-6.88 (m, 2H), 6.58 (d, *J* = 8.0 Hz, 1H), 5.18-5.14 (m, 1H), 4.69-4.65 (m, 1H), 3.44-3.34 (m, 2H), 3.04-2.92 (m, 2H), 1.24-1.17 (m, 2H), 1.06-0.99 (m, 2H), 0.65 (t, *J* = 7.3 Hz, 3H).

^{13}C NMR (150 MHz, DMSO) δ ppm: 168.1, 158.9, 137.7, 137.0, 136.4, 133.0, 130.7, 129.7, 129.3, 128.3, 127.6, 126.8, 126.0, 123.9, 121.8, 120.0, 110.0, 106.4, 65.9, 57.4, 41.2, 38.5, 30.8, 19.2, 13.4.

4-benzyl-2-(2-chloro-4-methylphenyl)-N-cyclohexyl-1-oxo-1,2,3,4-tetrahydropyrazino[1,2-a]indole-3-carboxamide (6n).

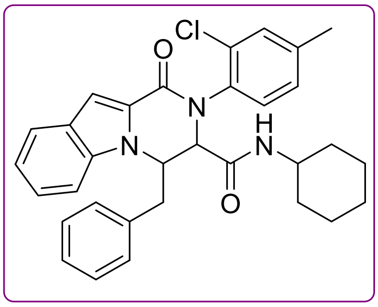

pale yellow powder, **mp:** 205-208 °C.

(**Yield:** 72%, **dr:** 63:37).

HRMS (ESI) m/z: Calcd for $C_{32}H_{32}ClN_3O_2$ $[M+H]^+$ 526.2261, Found 526.2265.

^{1}H NMR (600 MHz, $CDCl_3$) δ ppm: 7.74-7.69 (m, 1H), 7.67 (d, *J* = 6.9 Hz, 1H), 7.55-7.50 (m, 1H), 7.48-7.42 (m, 1H), 7.42-7.33 (m, 1H), 7.24 (d, *J* = 7.3 Hz, 1H), 7.21-7.17 (m, 1H), 7.17-7.08 (m, 3H), 7.07-7.00 (m, 2H), 6.98 (d, *J* = 6.7 Hz, 1H), 5.90-5.80 (m, 1H), 5.20-5.12 (m, 1H), 4.26-4.18 (m, 1H), 3.68-3.57 (m, 1H), 3.46-3.36 (m, 2H), 2.38 (s, 3H), 1.82-1.73 (m, 1H), 1.72-1.65 (m, 1H), 1.65-1.53 (m, 2H), 1.50-1.38 (m, 3H), 1.14-0.97 (m, 3H).

^{13}C NMR (150 MHz, $CDCl_3$) δ ppm: 167.7, 158.3, 139.8, 136.7, 136.5, 135.5, 132.4, 131.4, 130.9, 129.2, 128.9, 128.8, 127.8, 127.2, 127.0, 125.3, 122.8, 120.9, 109.8, 108.8, 68.1, 57.1, 48.7, 38.7, 32.2, 30.3, 28.9, 23.7.

2-(2-chloro-4-methylphenyl)-N-cyclohexyl-4-(4-methoxybenzyl)-1-oxo-1,2,3,4-tetrahydropyrazino[1,2-a]indole-3-carboxamide (6o).

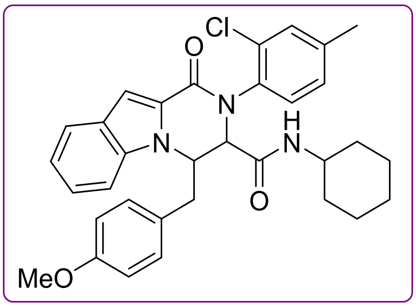

pale yellow powder, **mp:** 235-238 °C.

(**Yield:** 75%, **dr:** 64:36).

HRMS (ESI) m/z: Calcd for $C_{33}H_{34}ClN_3O_3$ $[M+H]^+$ 556.2367, Found 556.2373.

^{1}H NMR (600 MHz, $CDCl_3$) δ ppm: 7.67 (d, *J* = 7.7 Hz, 1H), 7.45-7.41 (m, 1H), 7.41-7.34 (m, 1H), 7.25-7.16 (m, 2H), 7.14-7.05 (m, 2H), 7.00 (d, *J* = 8.0 Hz, 1H), 6.95 (d, *J* = 7.1 Hz, 1H), 6.86 (d, *J* = 6.6 Hz, 1H), 6.69 (d, *J* = 7.1 Hz, 1H), 6.66 (d, *J* = 8.6 Hz, 1H), 5.87-5.80 (m, 1H), 5.14-5.06 (m, 1H), 4.40-4.33 (m, 1H), 3.71 (s, 3H), 3.67-3.58 (m, 1H), 3.40-3.30 (m, 2H), 2.38 (s, 3H), 1.82-1.69 (m, 2H), 1.61-1.54 (m, 1H), 1.49-1.42 (m, 1H), 1.39-1.16 (m, 3H), 1.15-0.96 (m, 3H).

^{13}C NMR (150 MHz, $CDCl_3$) δ ppm: 168.4, 158.8, 158.4, 139.7, 136.7, 135.6, 131.6, 131.4, 130.2, 128.9, 128.5, 127.8, 127.0, 126.5, 125.3, 122.8, 120.9, 114.3, 109.9, 108.7, 66.9, 57.3, 55.3, 48.7, 39.1, 32.2, 25.1, 24.2, 20.9.

4-benzyl-N-cyclohexyl-1-oxo-2-(p-tolyl)-1,2,3,4-tetrahydropyrazino[1,2-a]indole-3-carboxamide (6p).

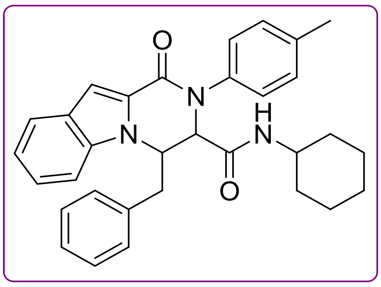

white powder, **mp:** 190-192 ^{o}C.

(**Yield:** 70%).

HRMS (ESI) m/z: Calcd for $C_{32}H_{33}N_3O_2$ $[M+H]^+$ 492.2651, Found 492.2661.

^{1}H NMR (300 MHz, $CDCl_3$) δ ppm: 7.74 (d, *J* = 7.7 Hz, 1H), 7.46 (s, 1H), 7.43-7.27 (m, 5H), 7.25-7.10 (m, 5H), 7.07-6.92 (m, 2H), 6.29-6.09 (m, 1H), 5.57-5.40 (m, 1H), 4.68-4.53 (m, 1H), 3.76-3.58 (m, 1H), 3.40-3.10 (m, 2H), 2.46 (s, 3H), 1.92-1.78 (m, 1H), 1.72-1.59 (m, 1H), 1.58-1.41 (m, 2H), 1.41-1.24 (m, 2H), 1.23-0.99 (m, 3H), 0.91-0.71 (m, 1H).

^{13}C NMR (75 MHz, $CDCl_3$) δ ppm: 168.0, 158.8, 138.6, 136.8, 136.5, 136.0, 130.1, 129.2, 128.8, 127.3, 127.1, 126.9, 125.3, 124.2, 122.7, 120.9. 110.0, 108.5, 66.9, 55.9, 48.7, 39.9, 32.5, 25.2, 24.5, 21.0.

^{1}H-NMR (600 MHz, $CDCl_3$) 5a

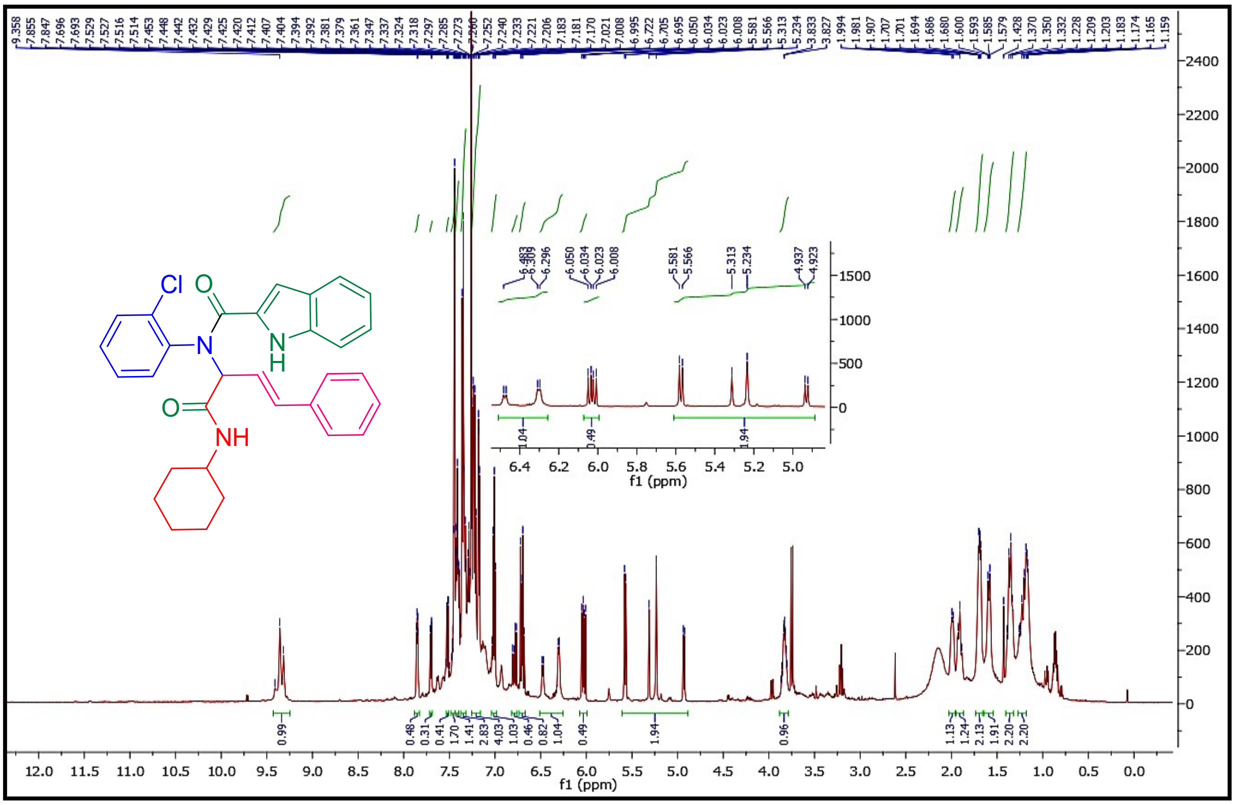

^{13}C-NMR (150 MHz, $CDCl_3$) 5a

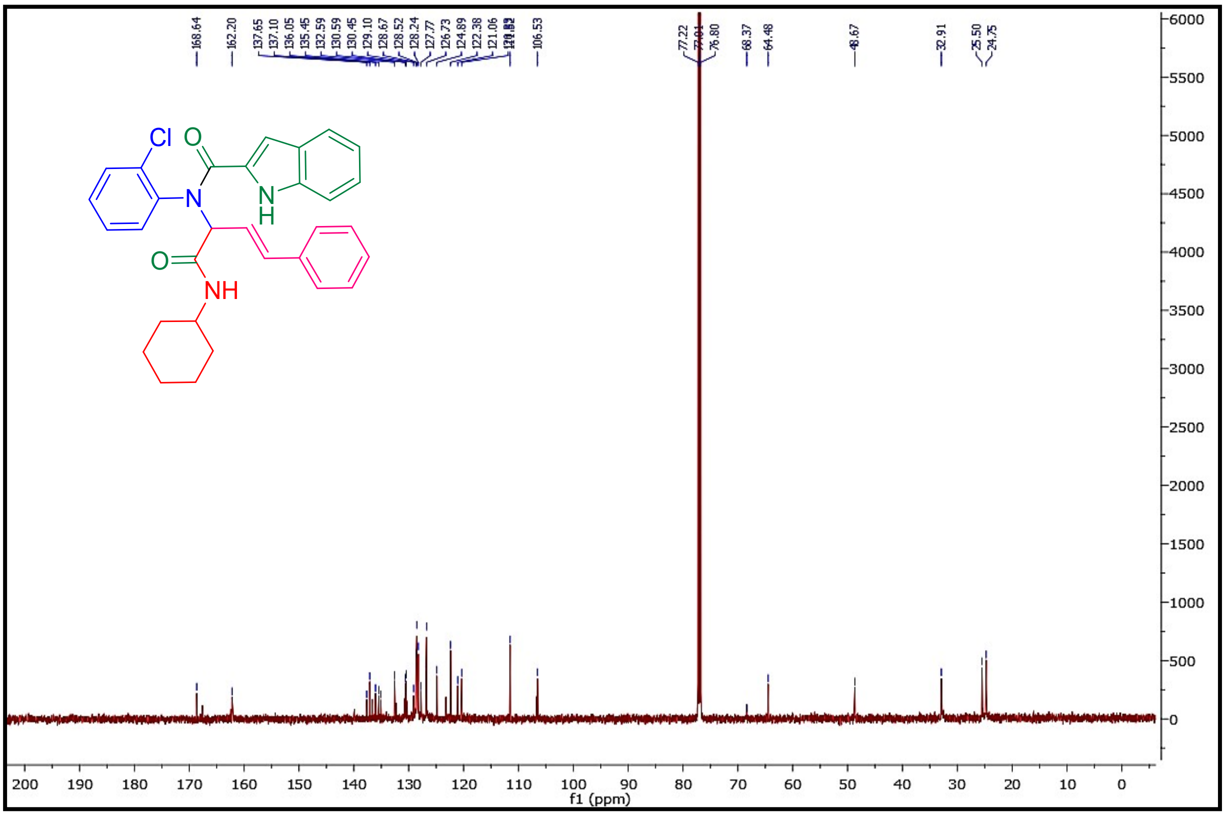

^{1}H-NMR (600 MHz, $CDCl_3$) 5b

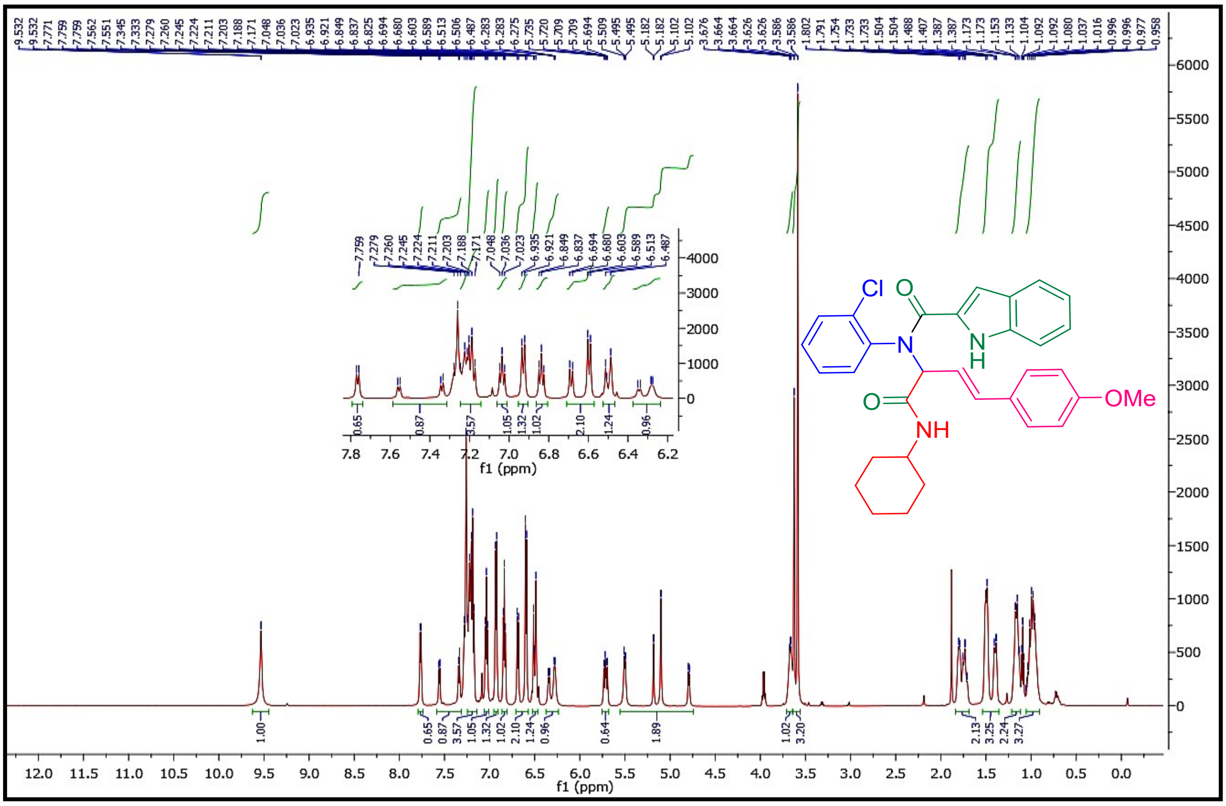

^{13}C-NMR (150 MHz, $CDCl_3$) 5b

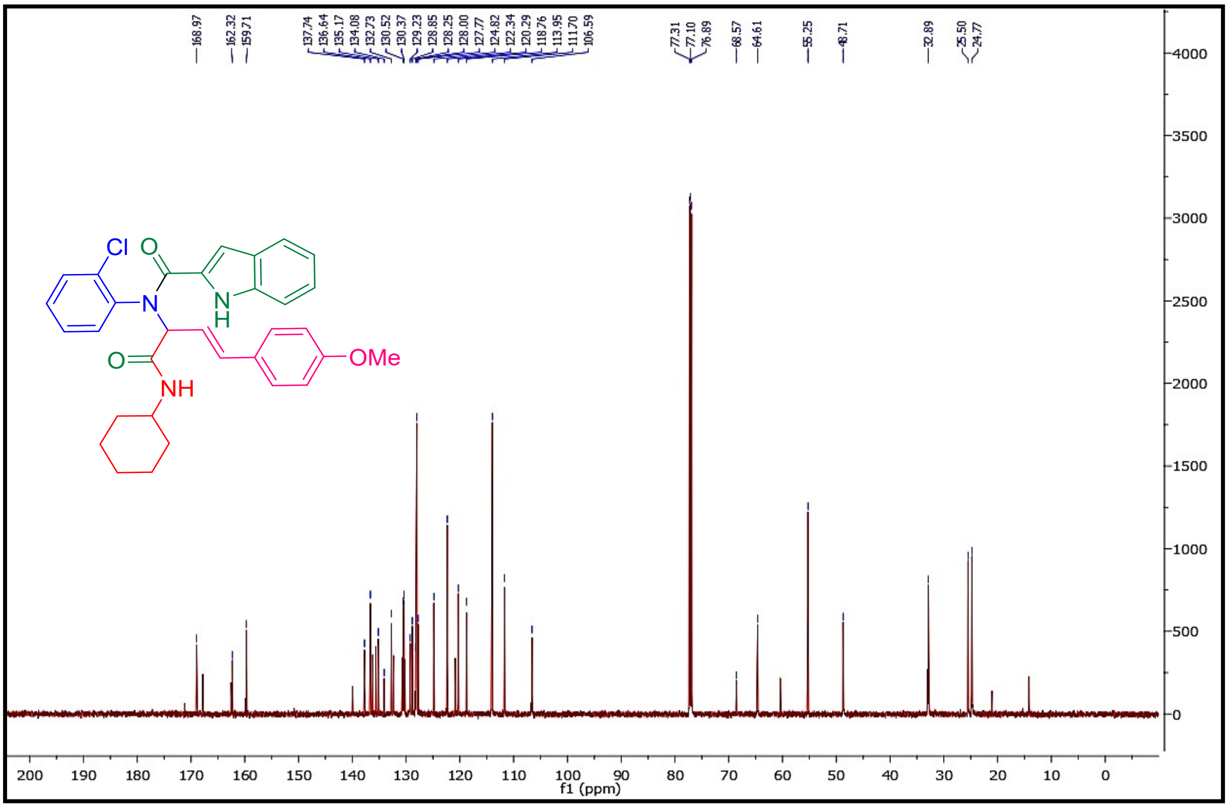

^{1}H-NMR (600 MHz, $CDCl_3$) 5c

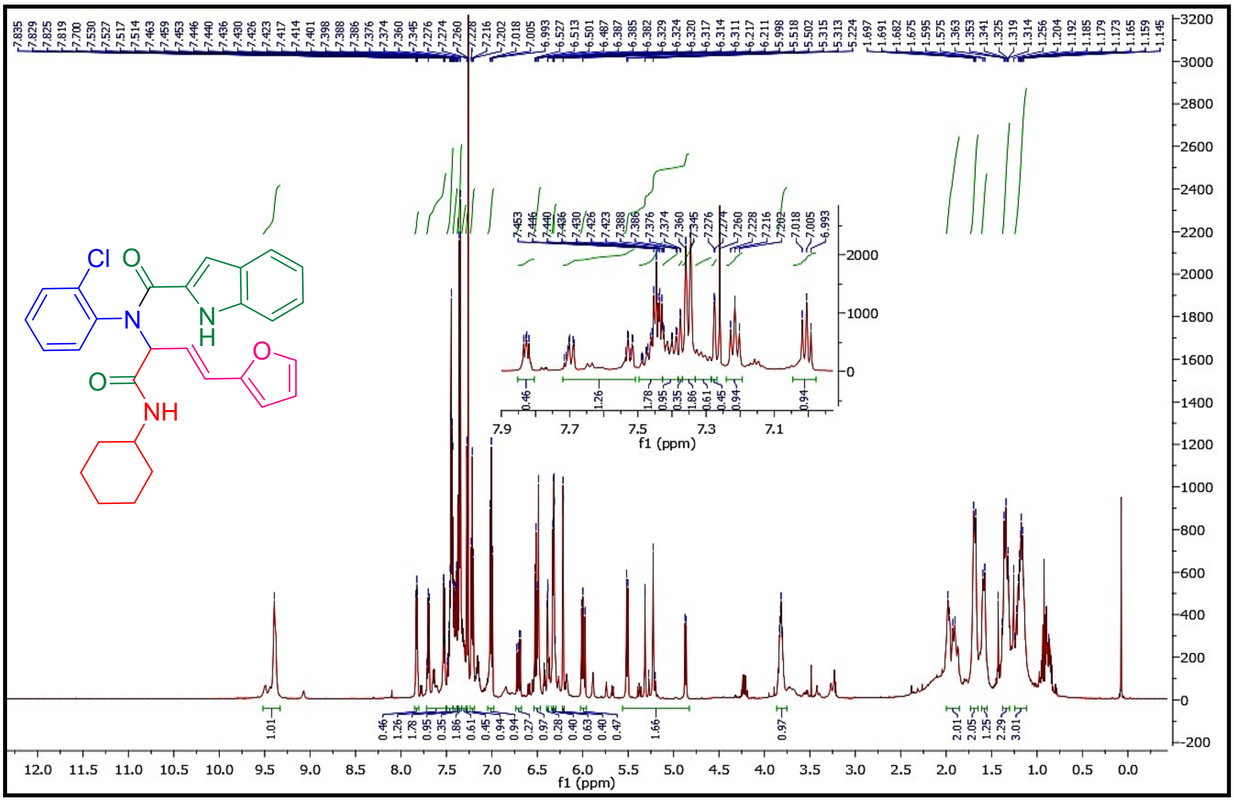

^{13}C-NMR (150 MHz, $CDCl_3$) 5c

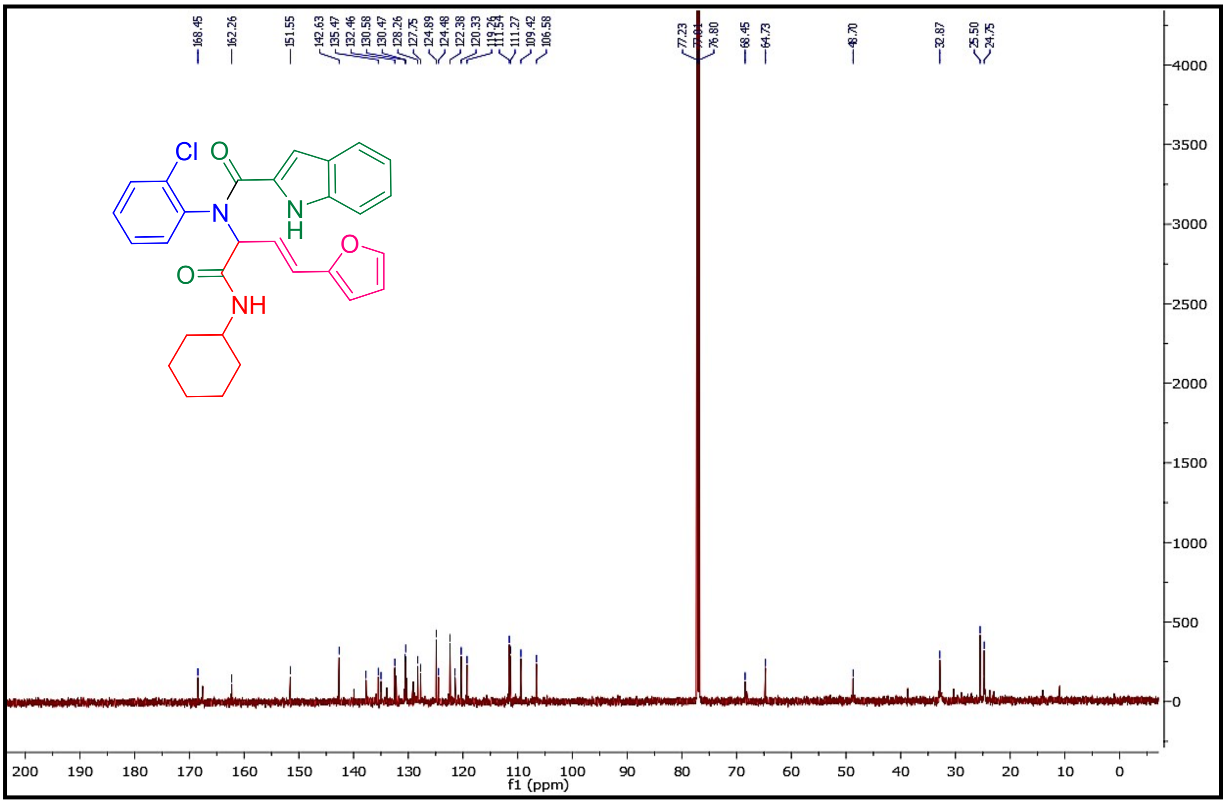

^{1}H-NMR (600 MHz, $CDCl_3$) 5d

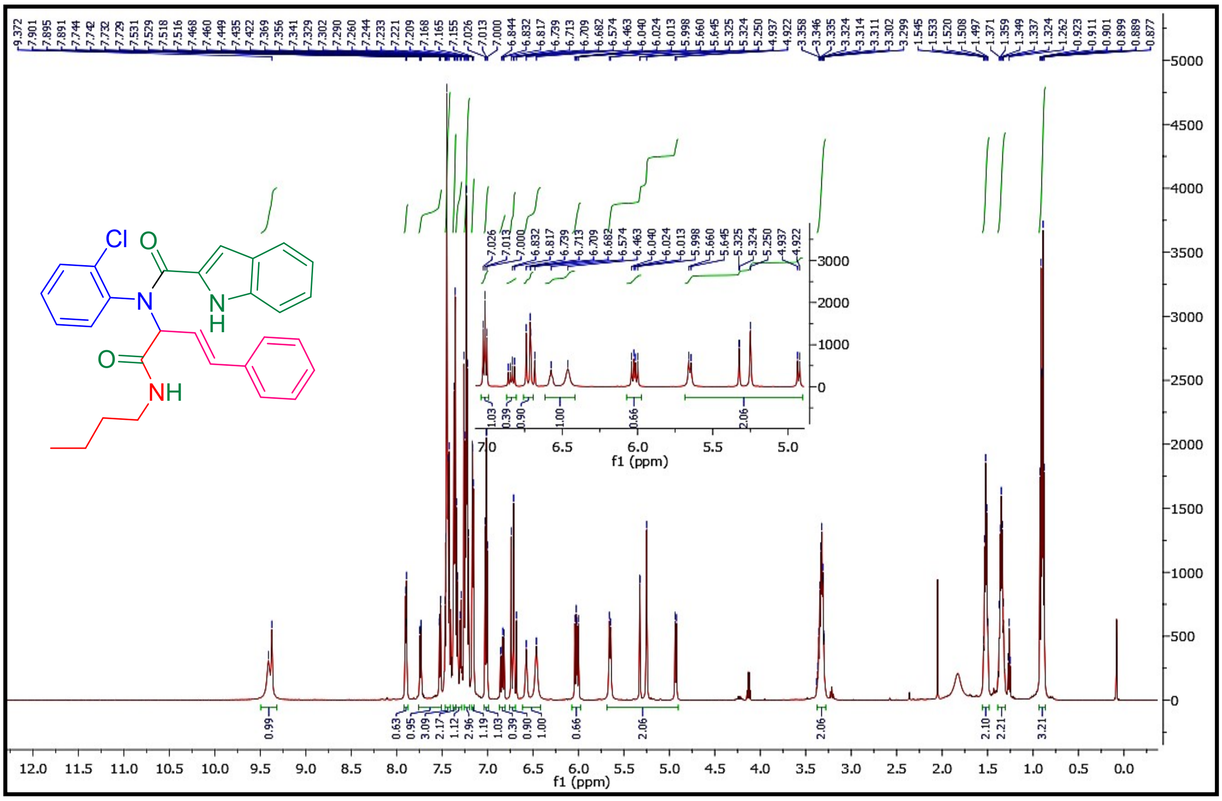

^{13}C-NMR (150 MHz, $CDCl_3$) 5d

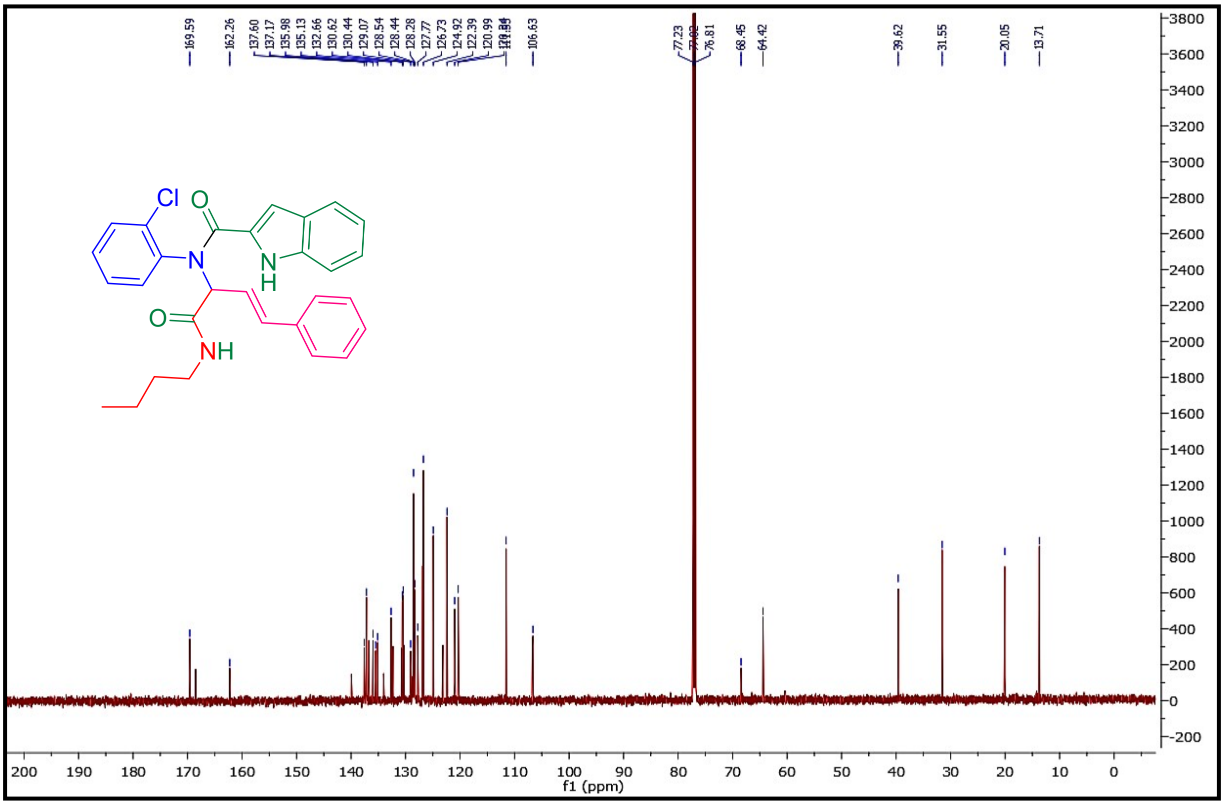

^{1}H-NMR (600 MHz, $CDCl_3$) 5e

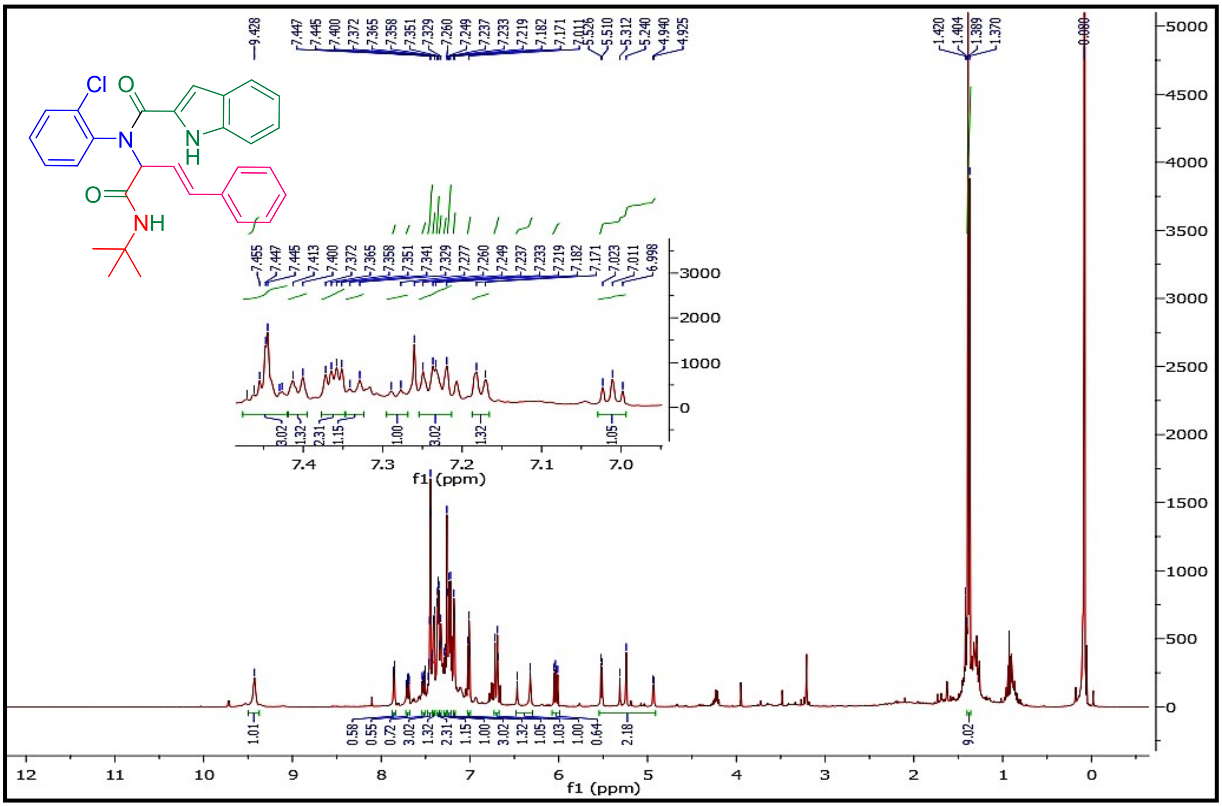

^{13}C-NMR (150 MHz, $CDCl_3$) 5e

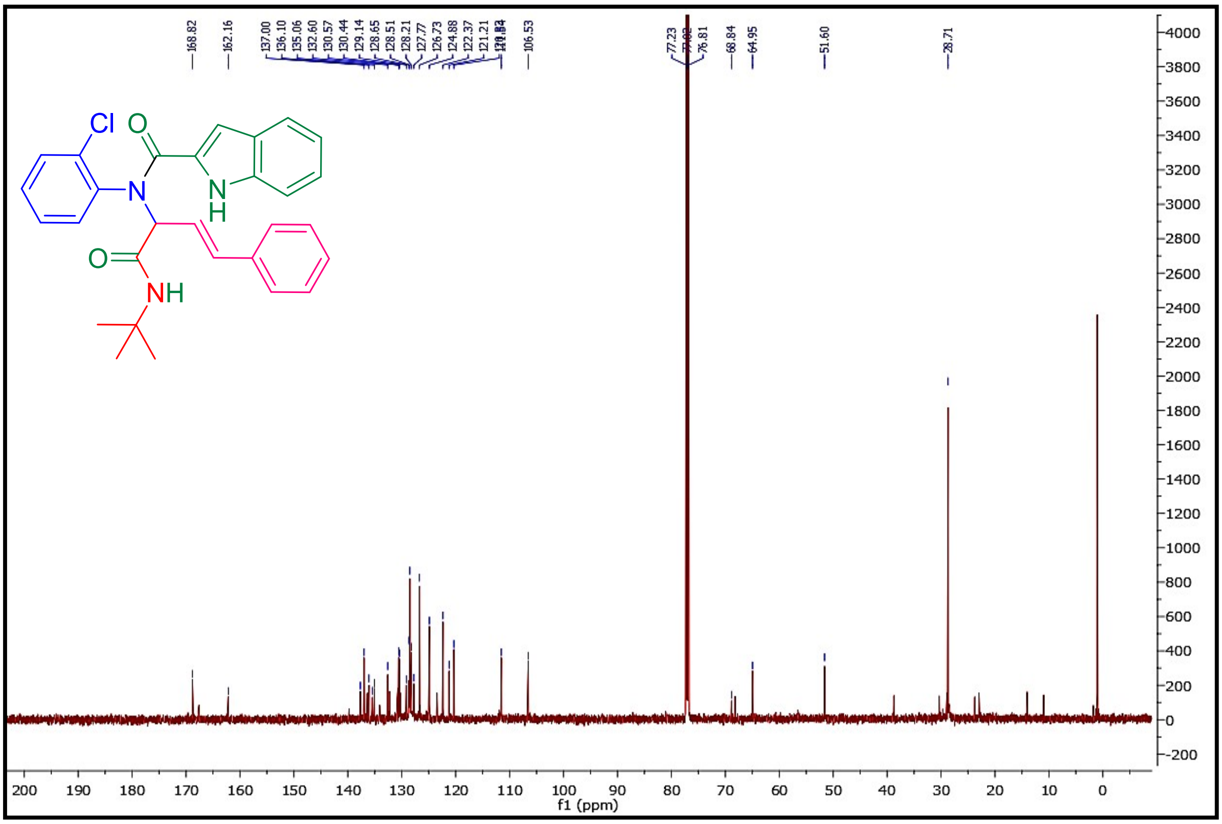

^{1}H-NMR (600 MHz, $CDCl_3$) 5f

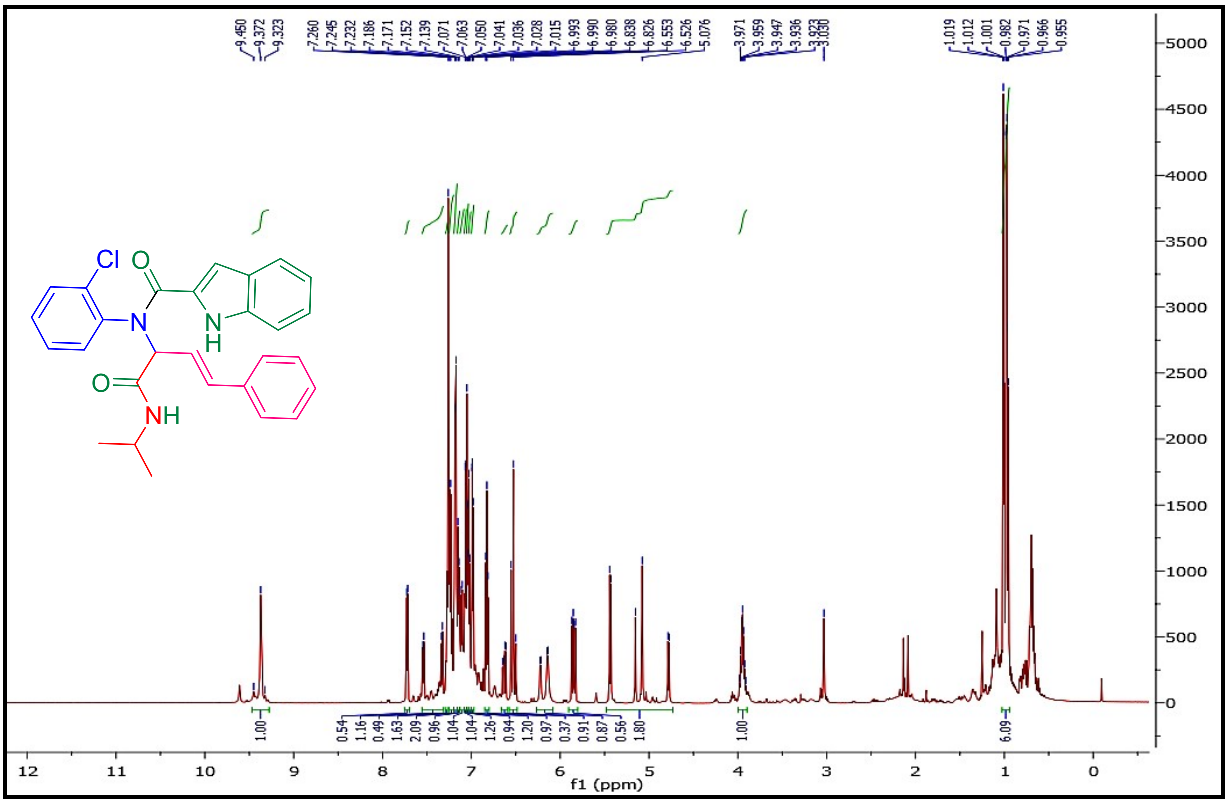

^{13}C-NMR (150 MHz, $CDCl_3$) 5f

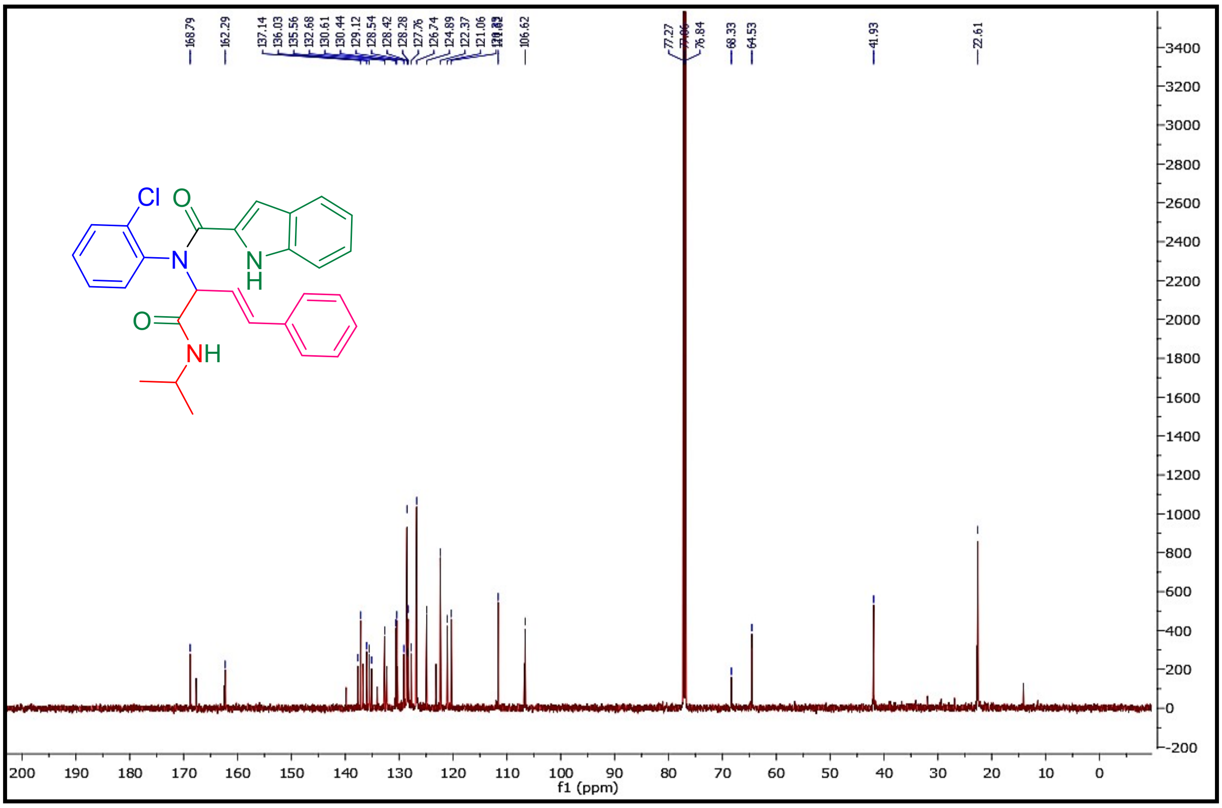

^{1}H-NMR (600 MHz, $CDCl_3$) 5g

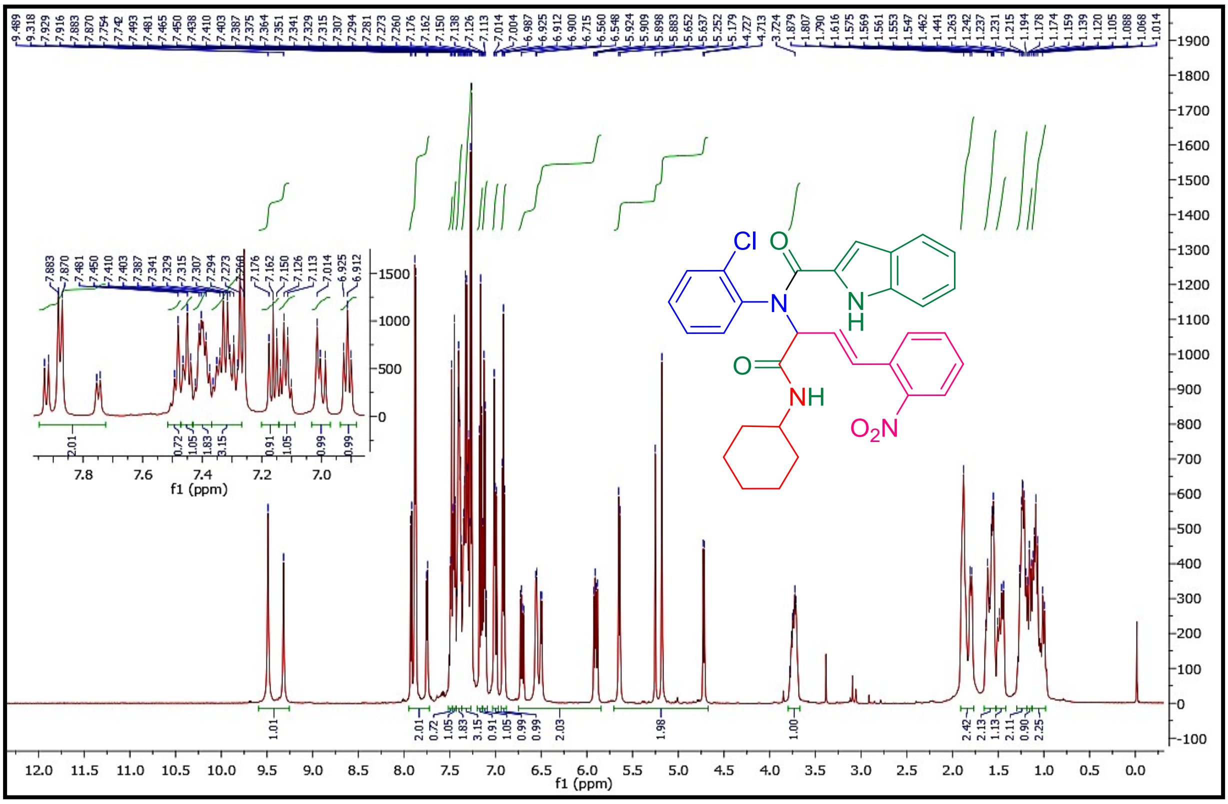

^{13}C-NMR (150 MHz, $CDCl_3$) 5g

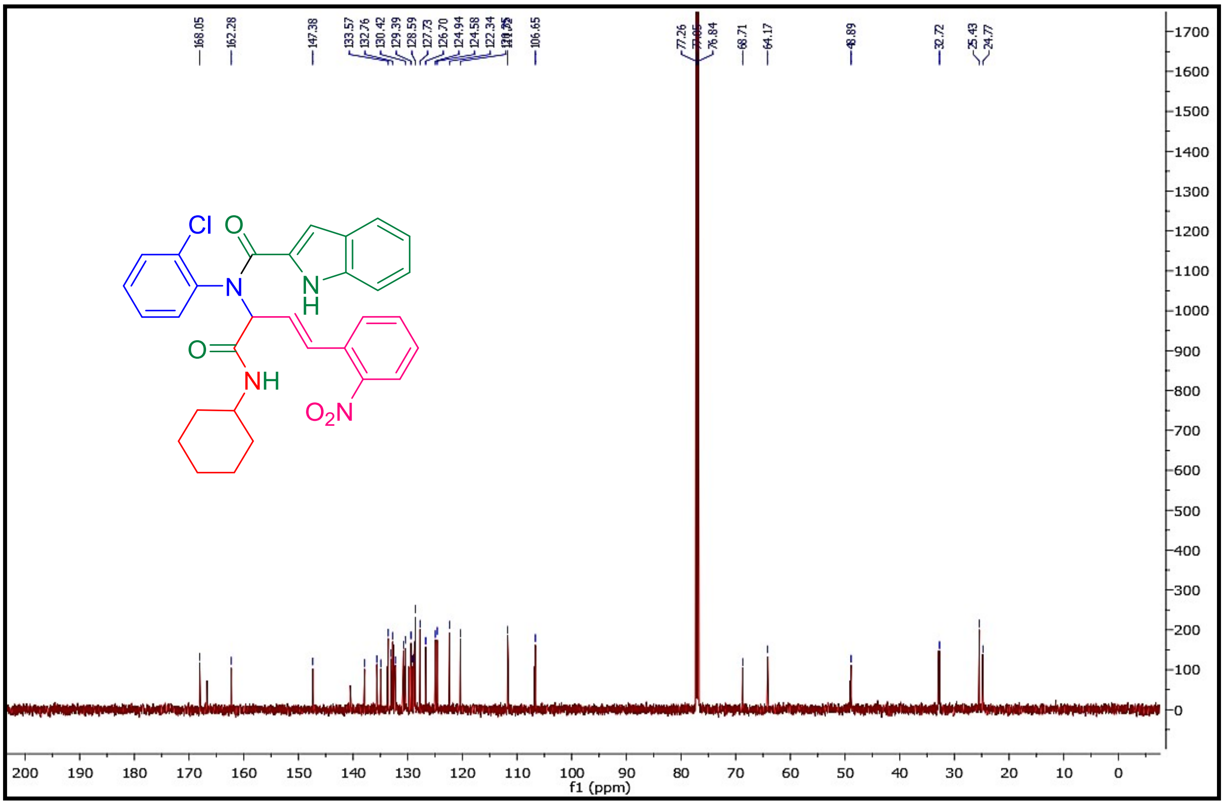

^{1}H-NMR (600 MHz, $CDCl_3$) 5h

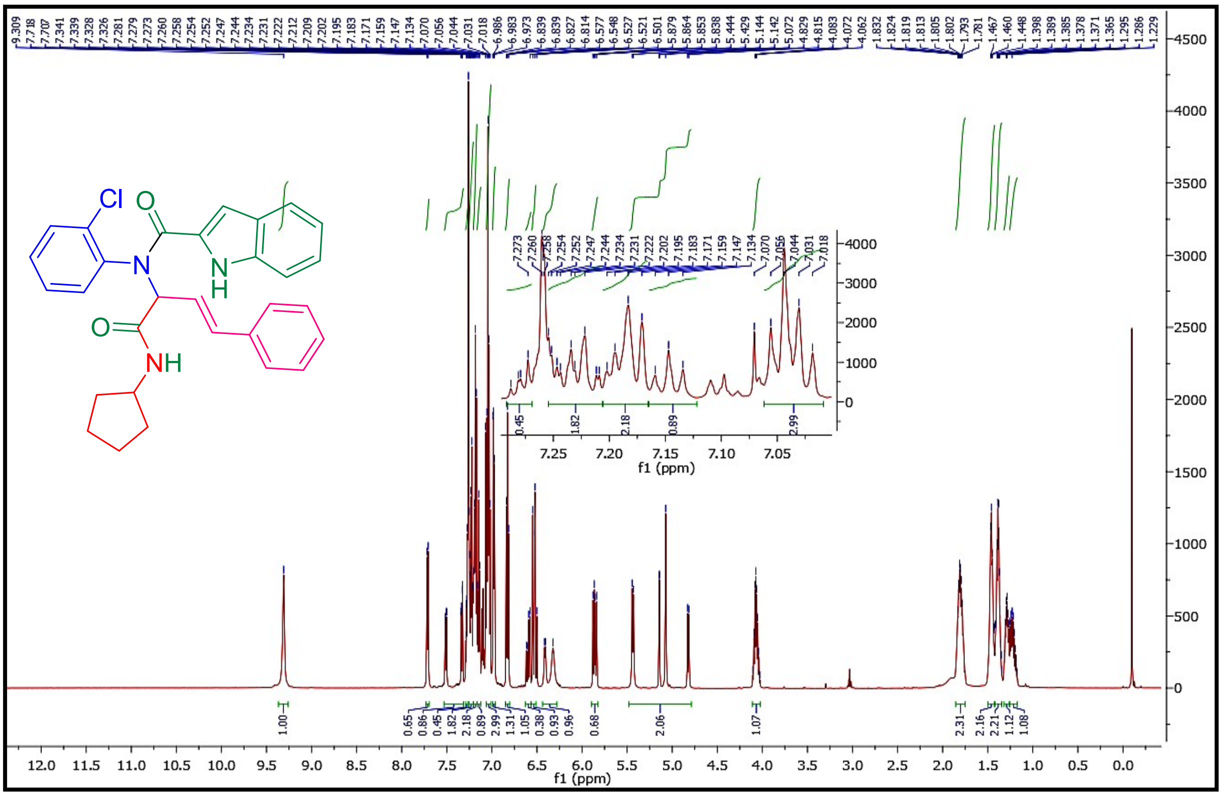

^{13}C-NMR (150 MHz, $CDCl_3$) 5h

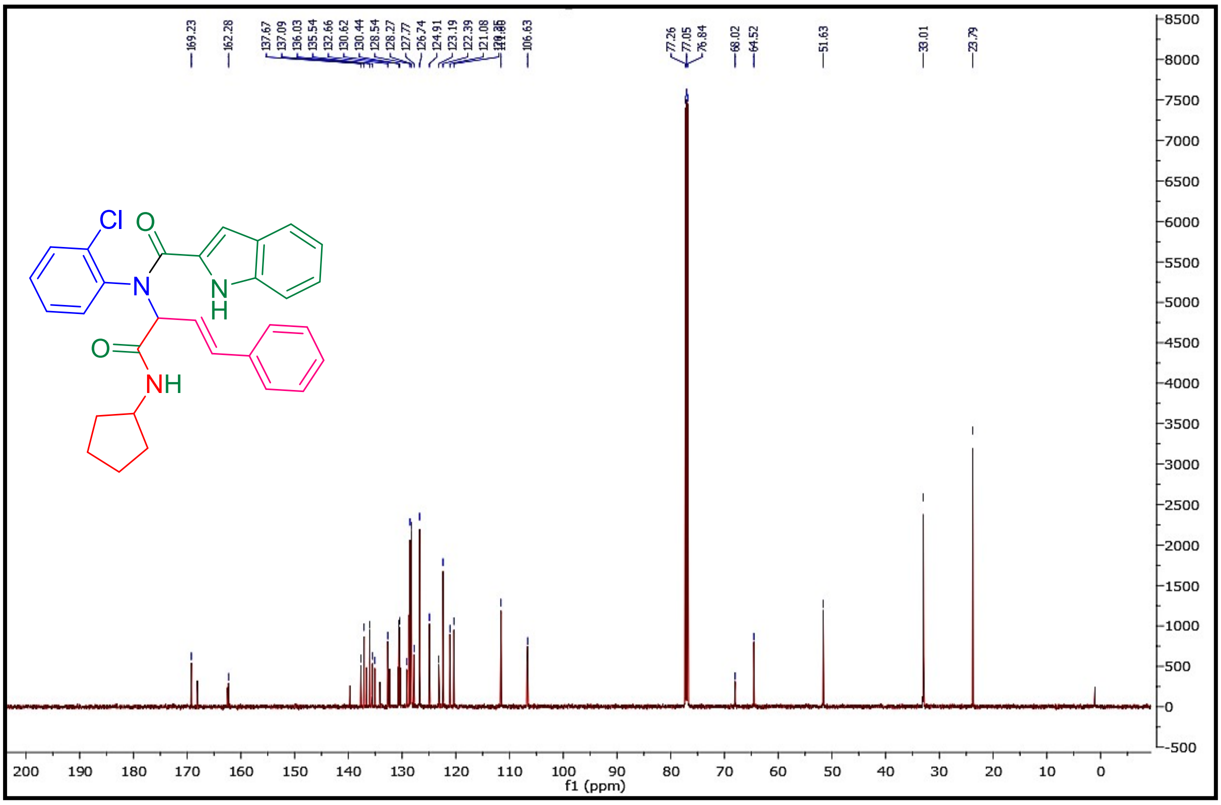

^{1}H-NMR (600 MHz, $CDCl_3$) 5i

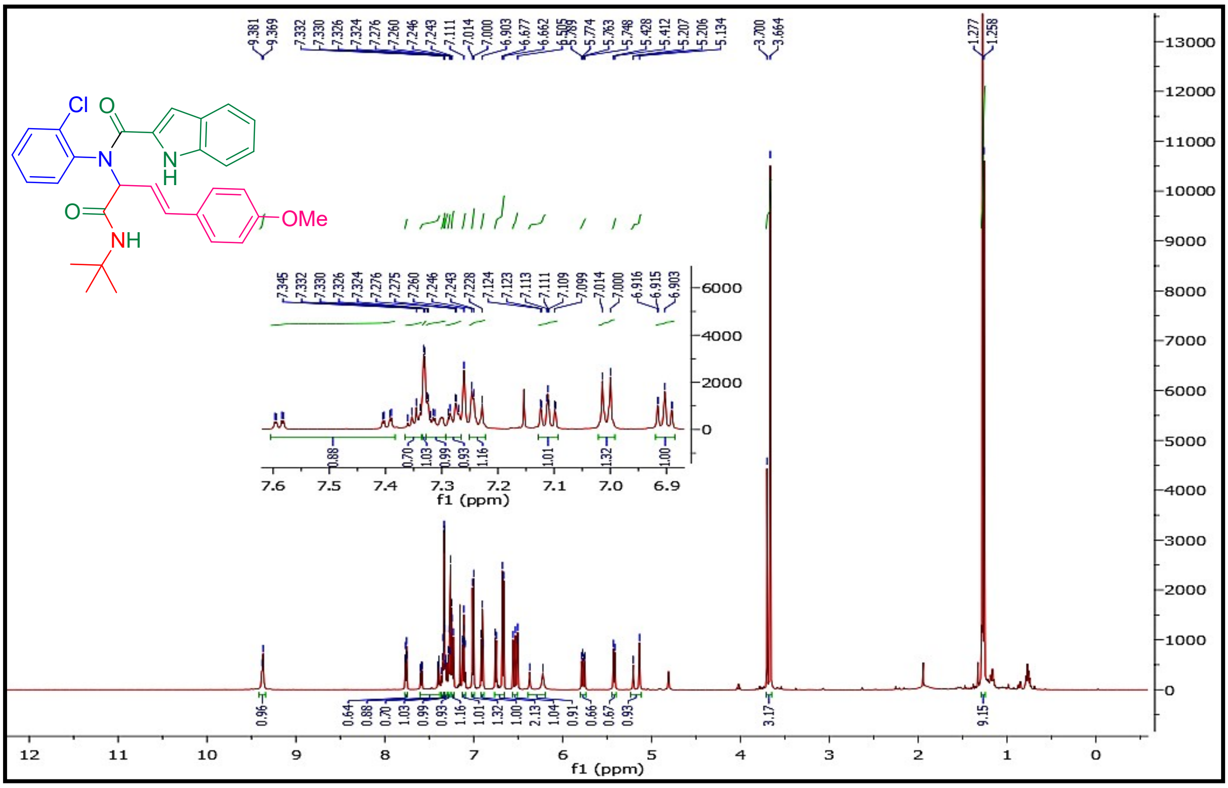

^{13}C-NMR (150 MHz, $CDCl_3$) 5i

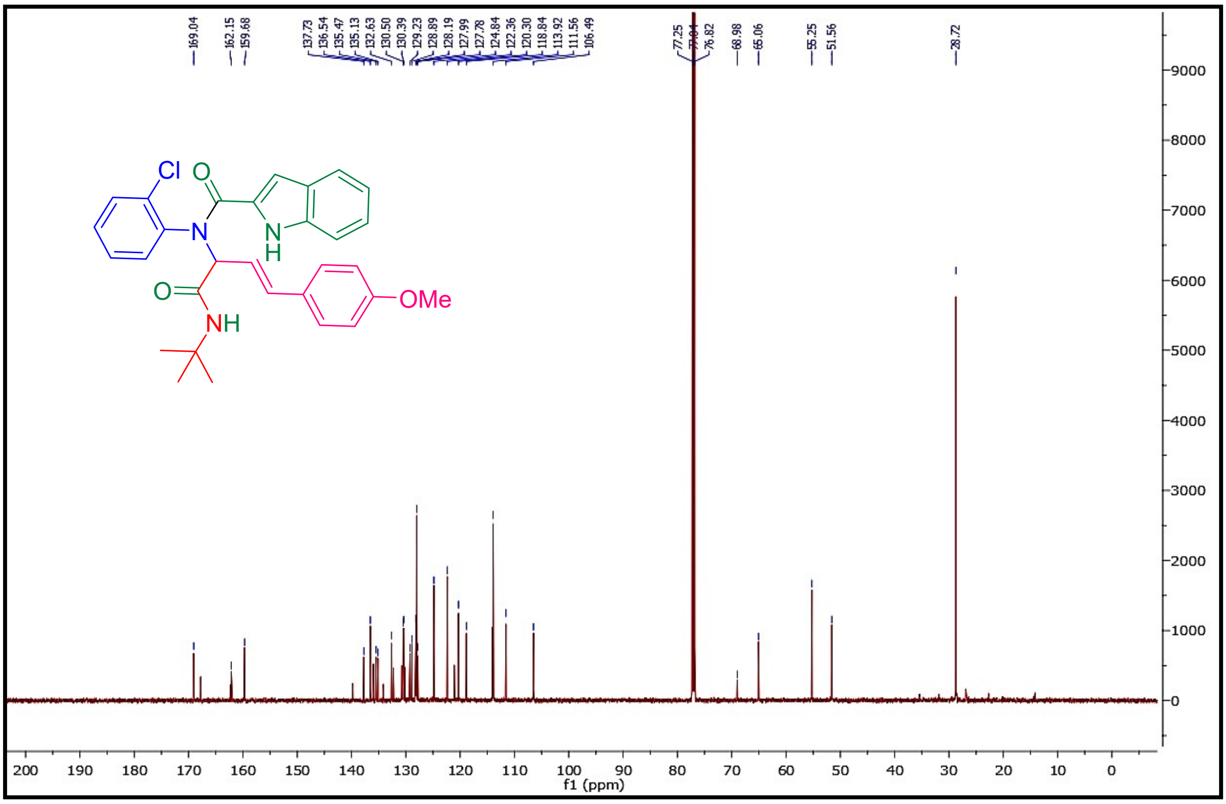

^{1}H-NMR (600 MHz, $CDCl_3$) 5j

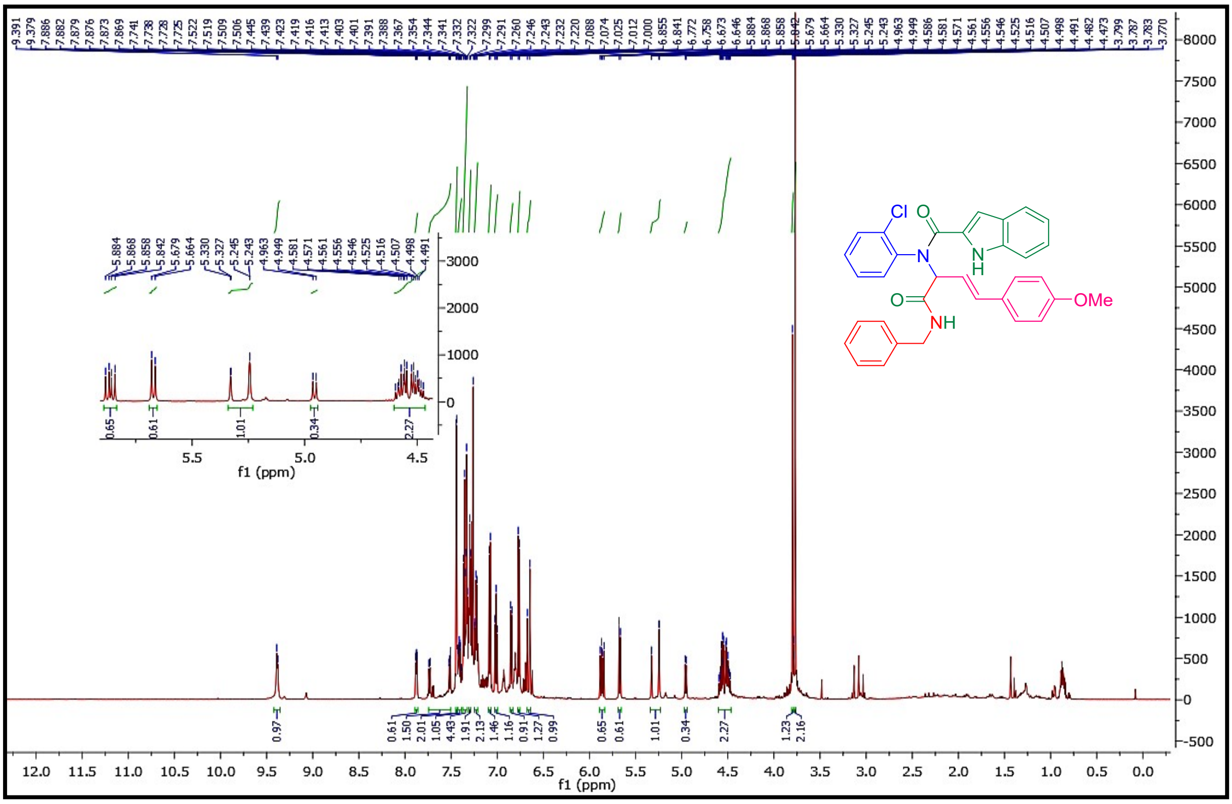

^{13}C-NMR (150 MHz, $CDCl_3$) 5j

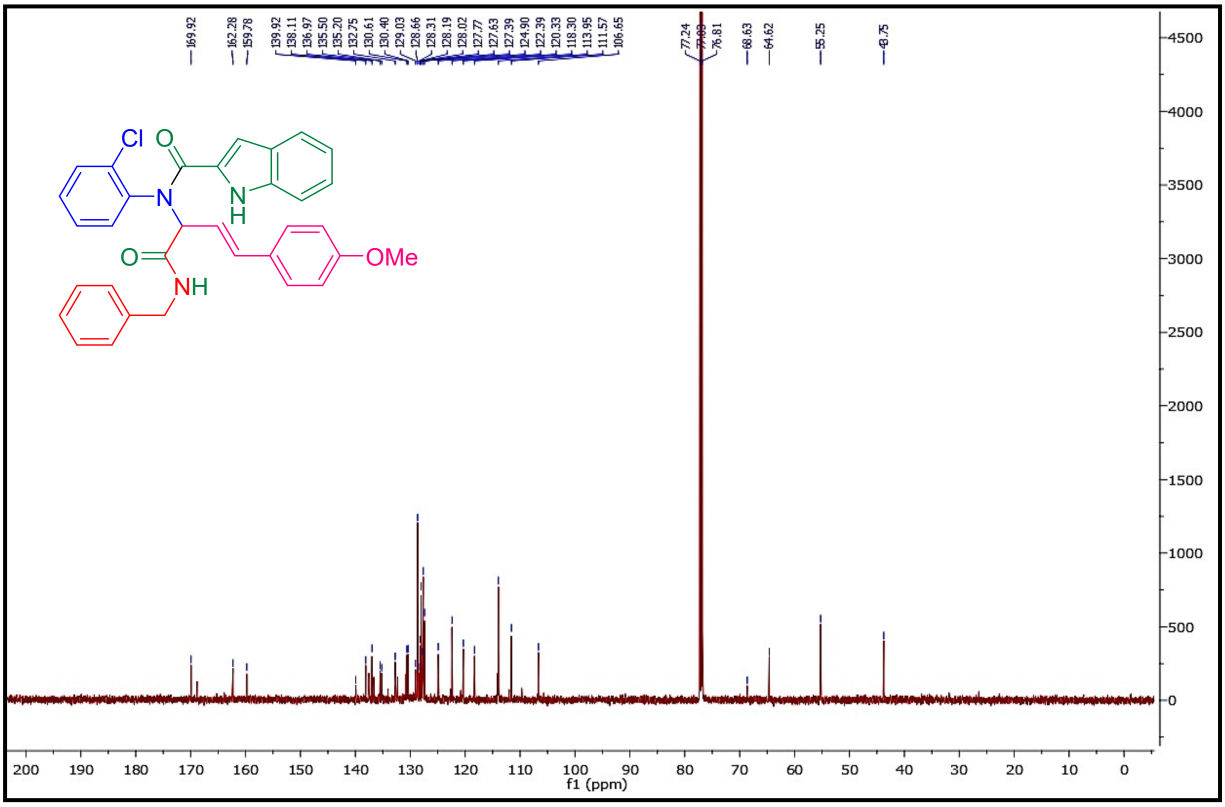

^{1}H-NMR (600 MHz, CDCl$_3$) 5k

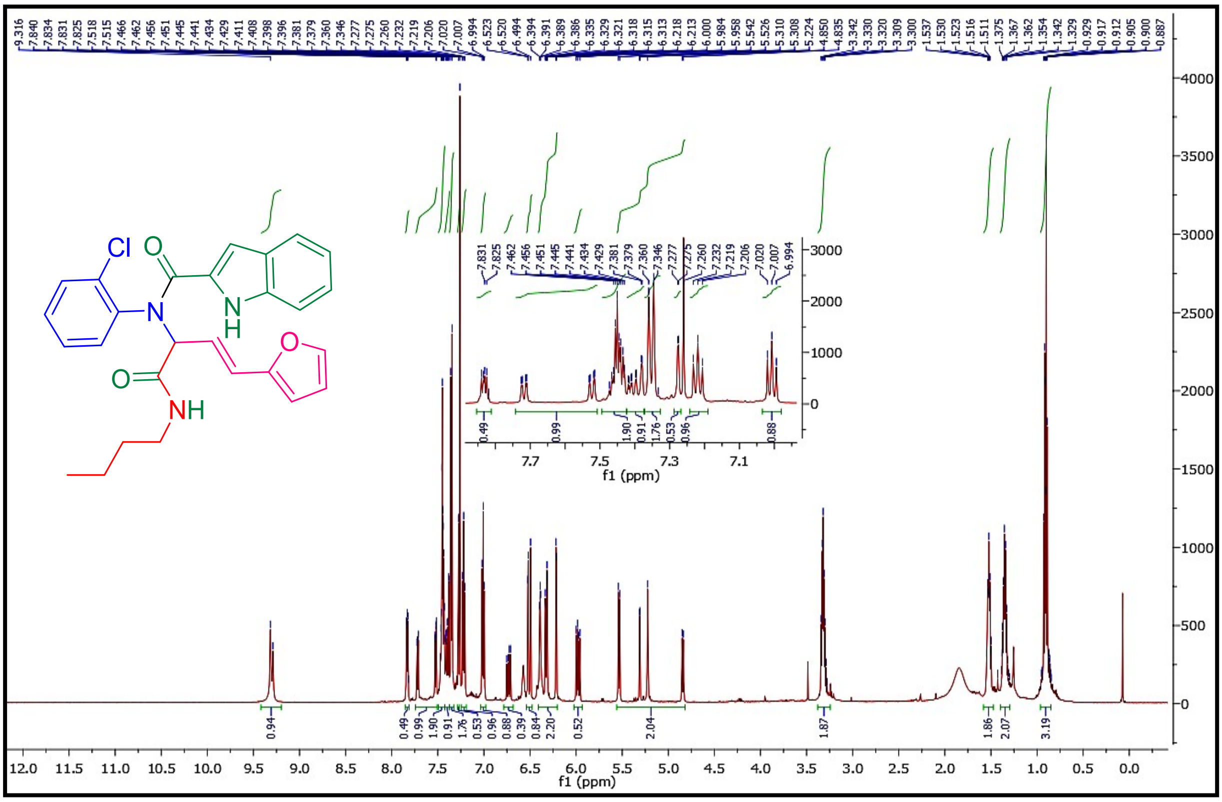

^{13}C-NMR (150 MHz, CDCl$_3$) 5k

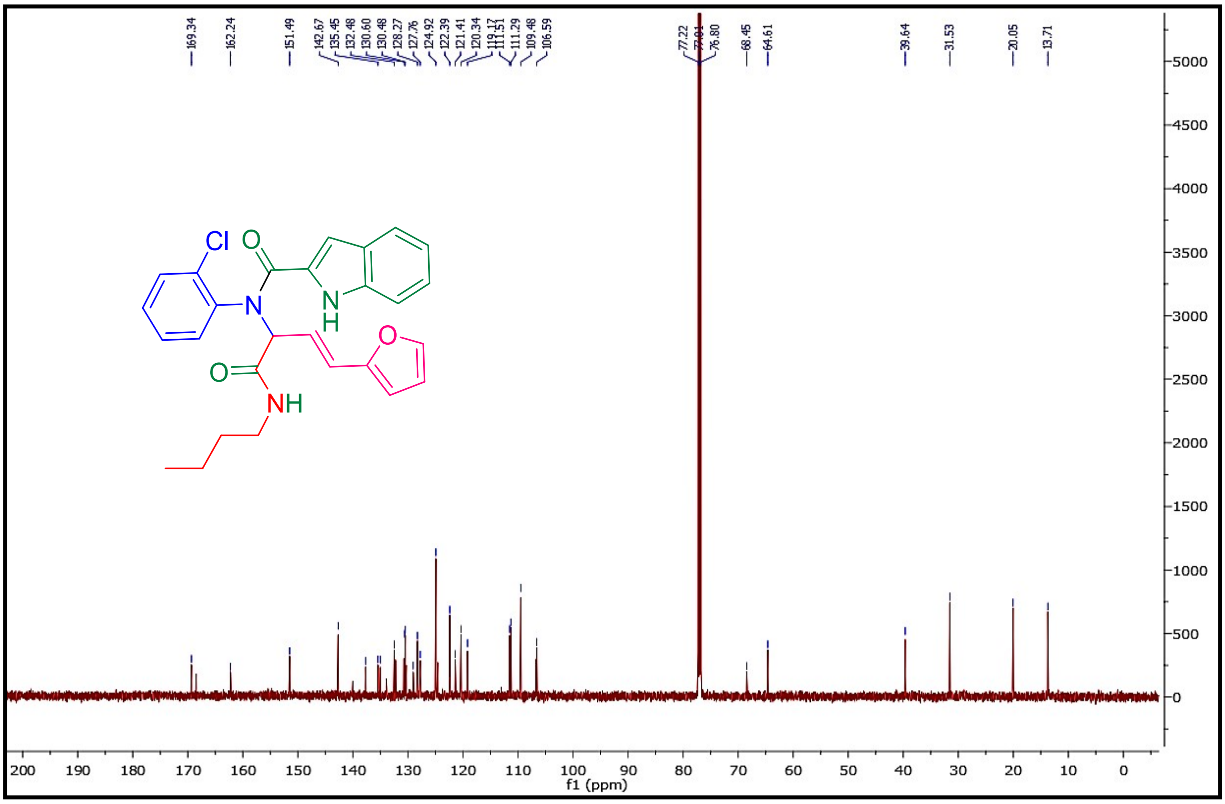

^{1}H-NMR (600 MHz, $CDCl_3$) 5l

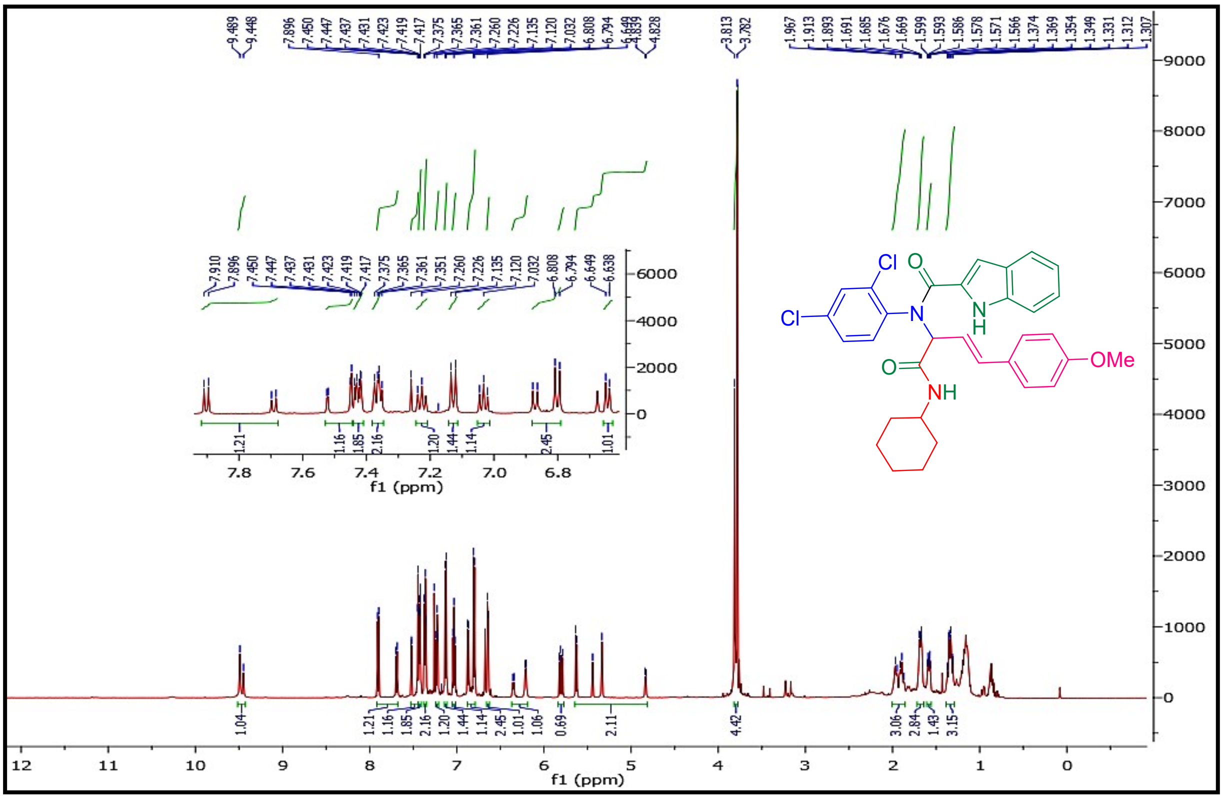

^{13}C-NMR (150 MHz, $CDCl_3$) 5l

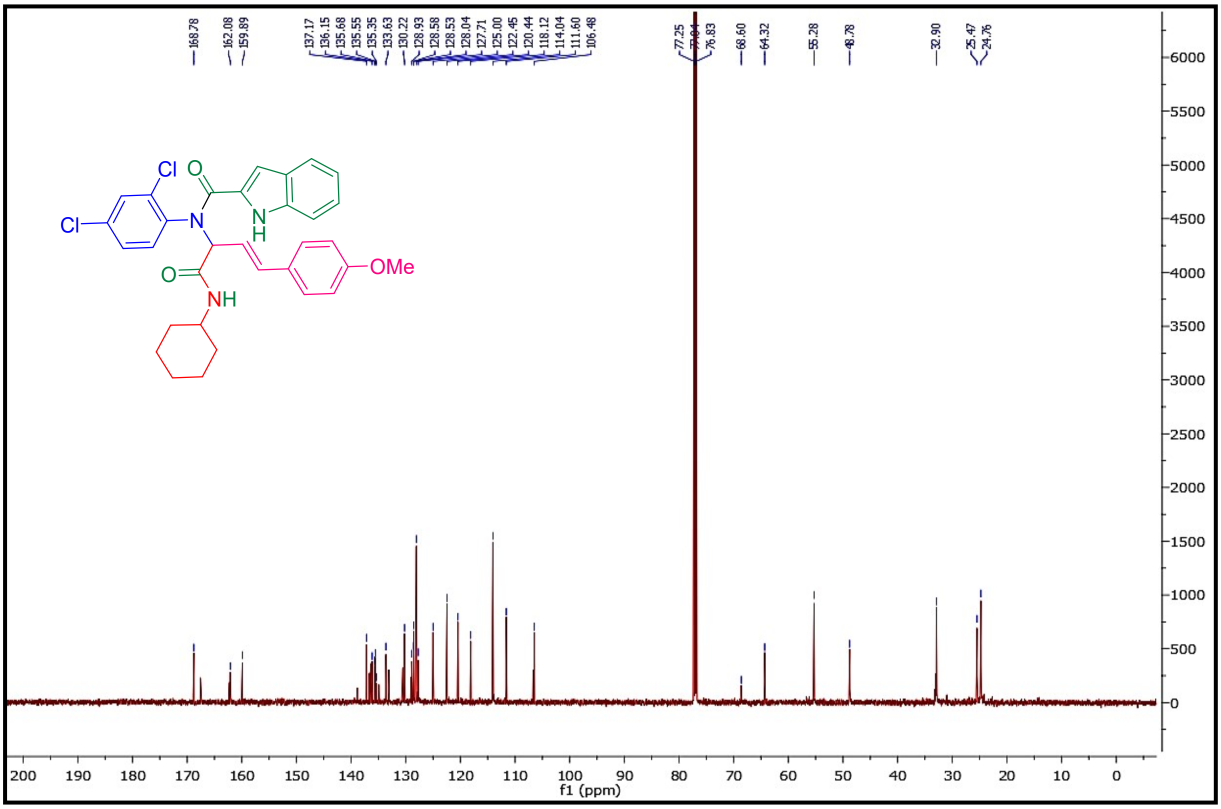

^{1}H-NMR (600 MHz, DMSO-d*6*) 5m

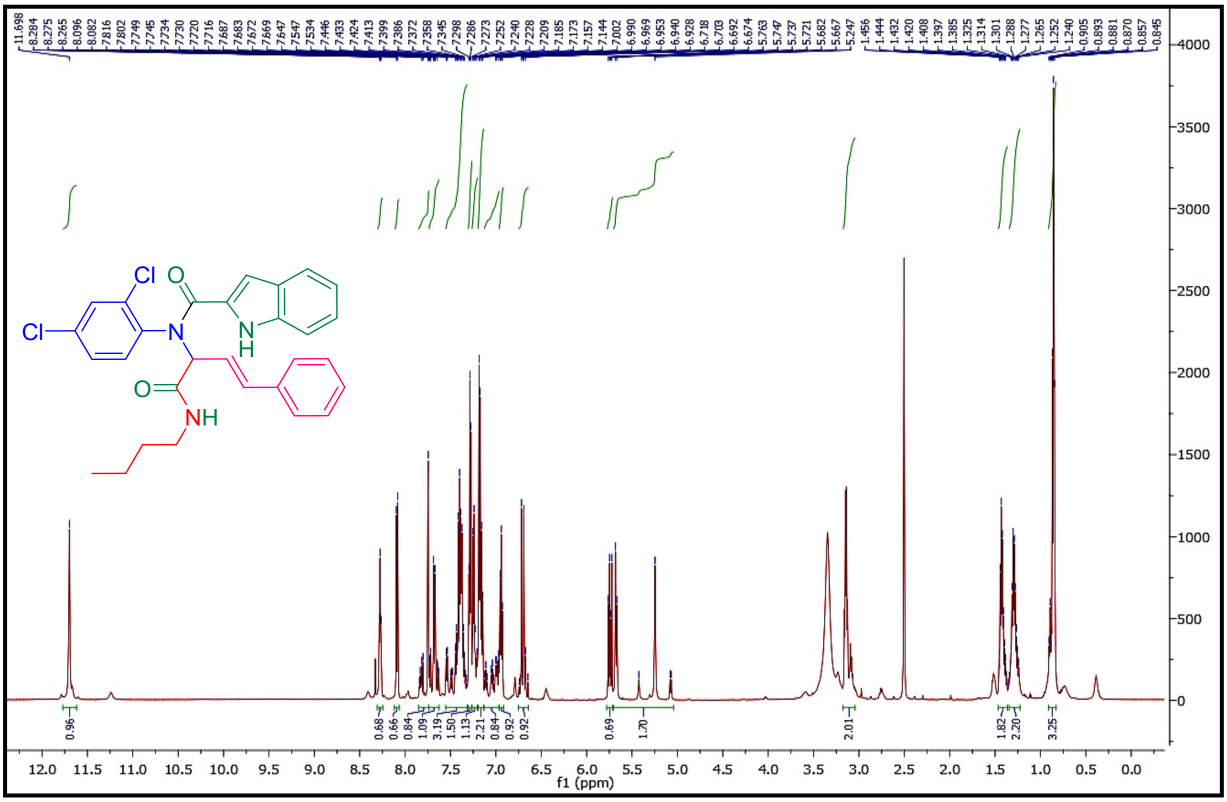

^{13}C-NMR (150 MHz, DMSO-d*6*) 5m

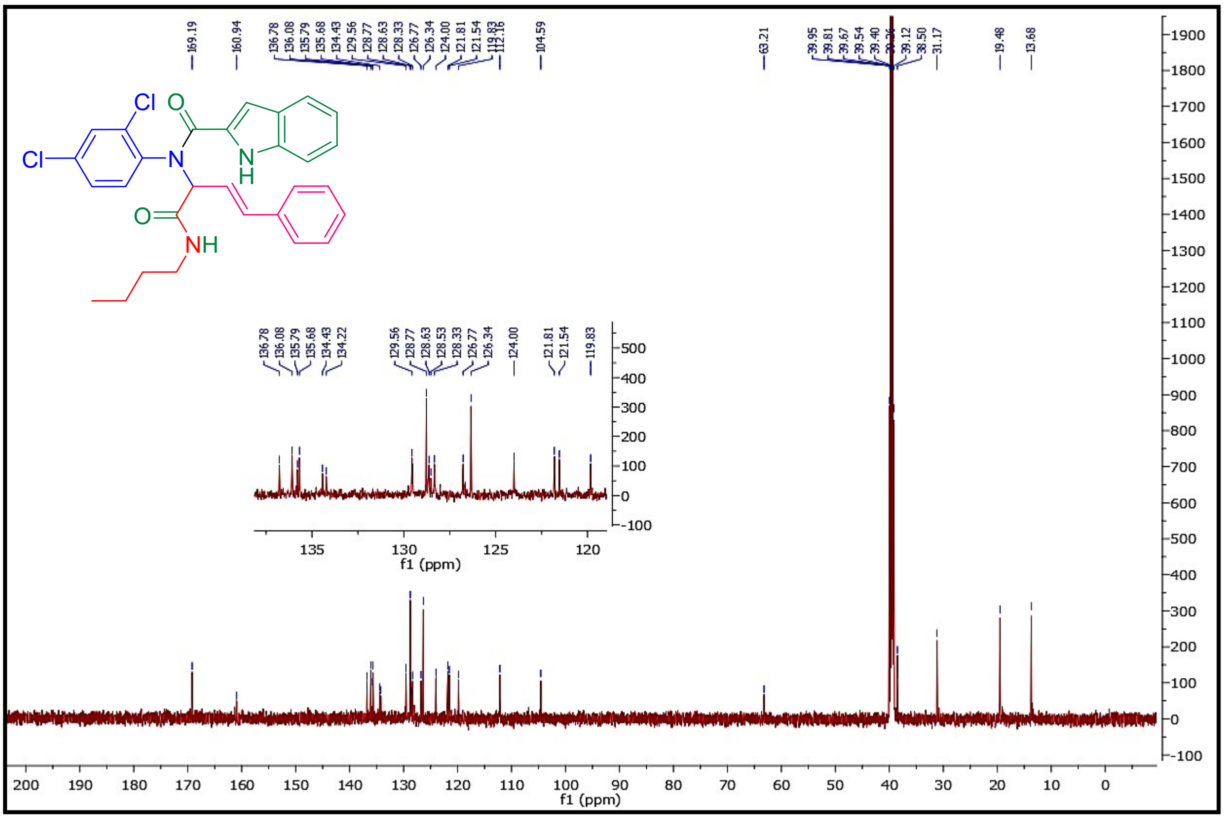

^{1}H-NMR (600 MHz, $CDCl_3$) 5n

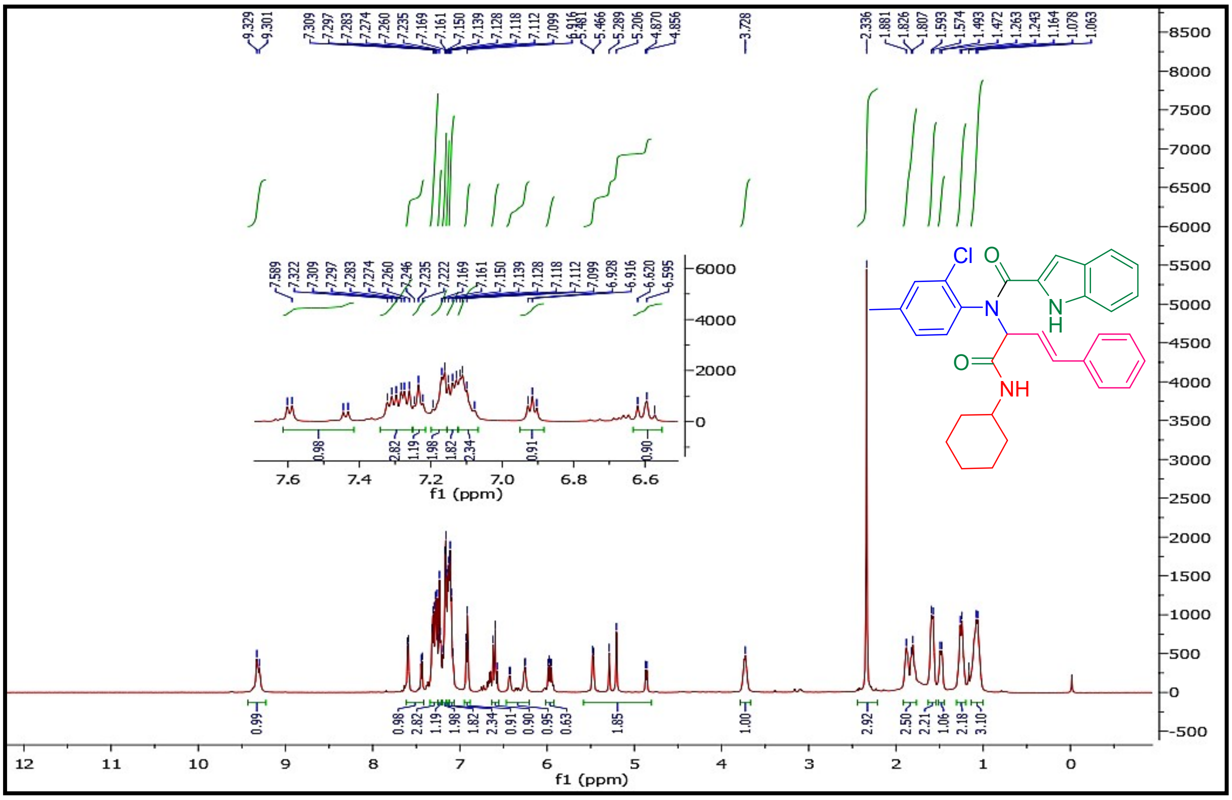

^{13}C-NMR (150 MHz, $CDCl_3$) 5n

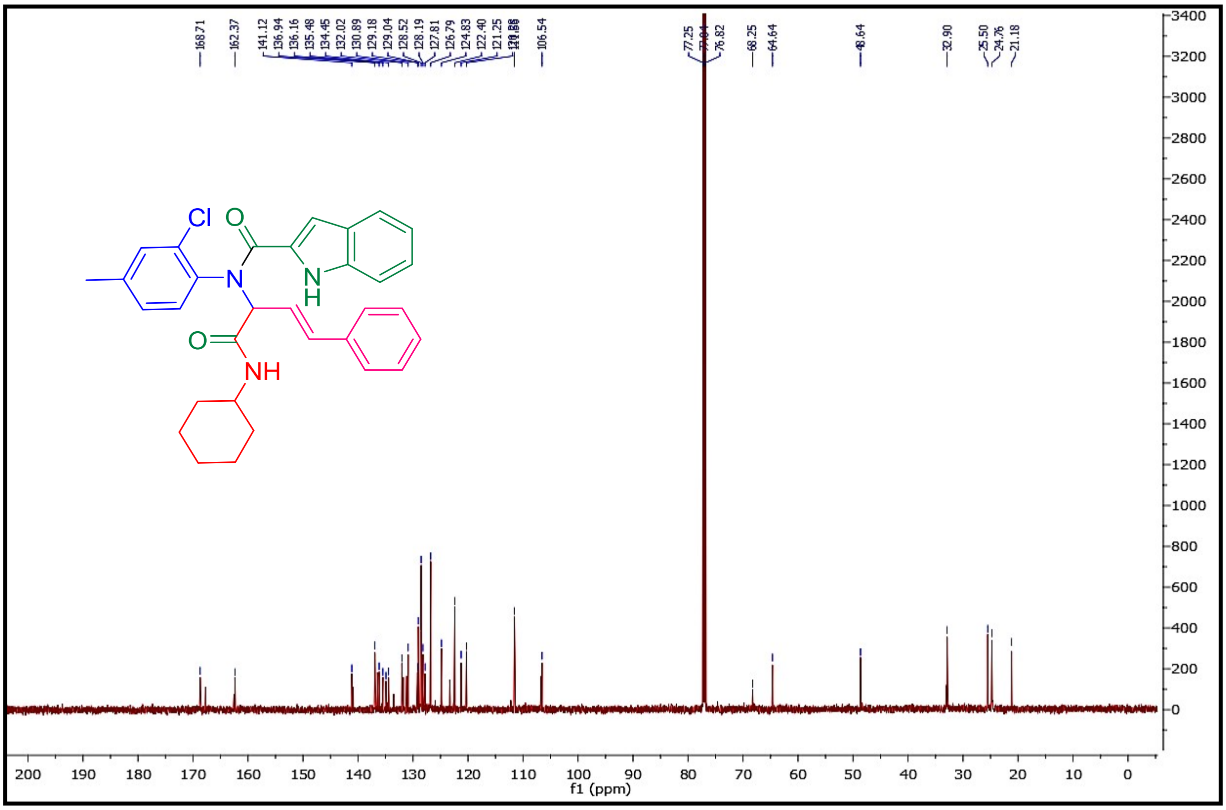

¹H-NMR (600 MHz, $CDCl_3$) 5o

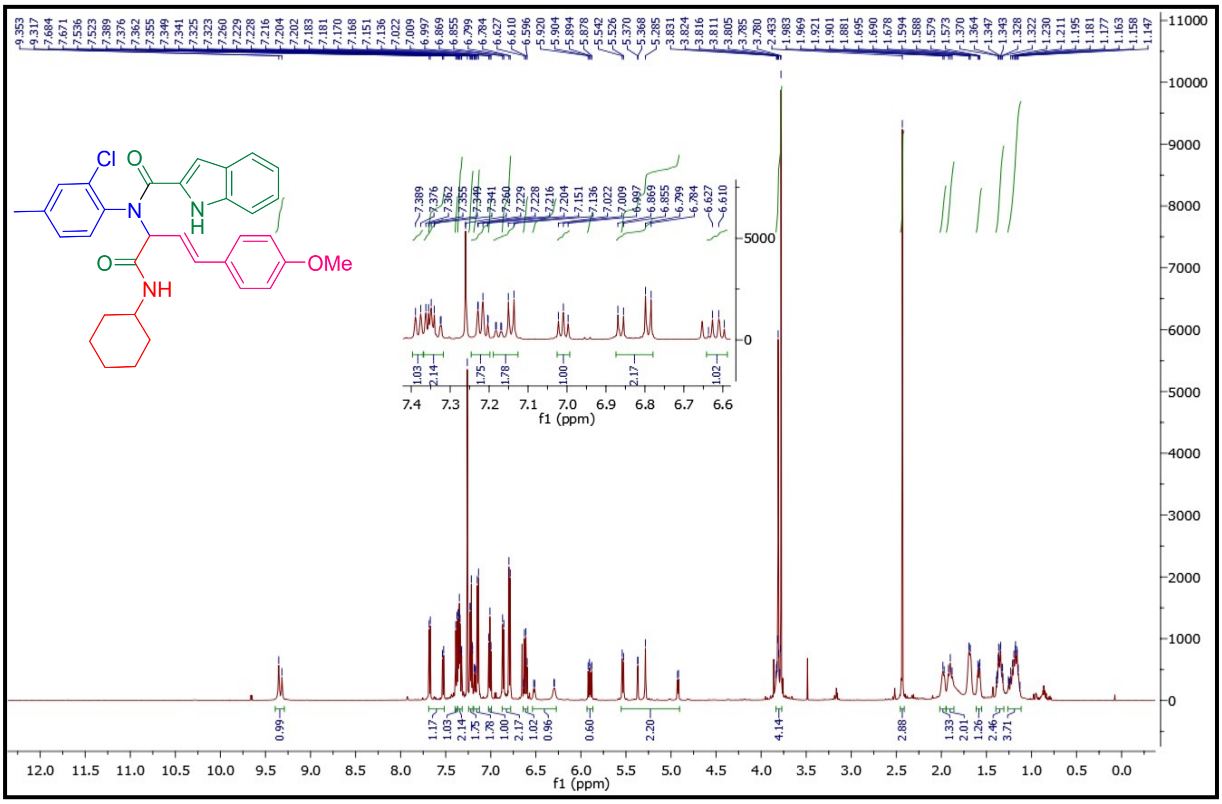

¹³C-NMR (150 MHz, $CDCl_3$) 5o

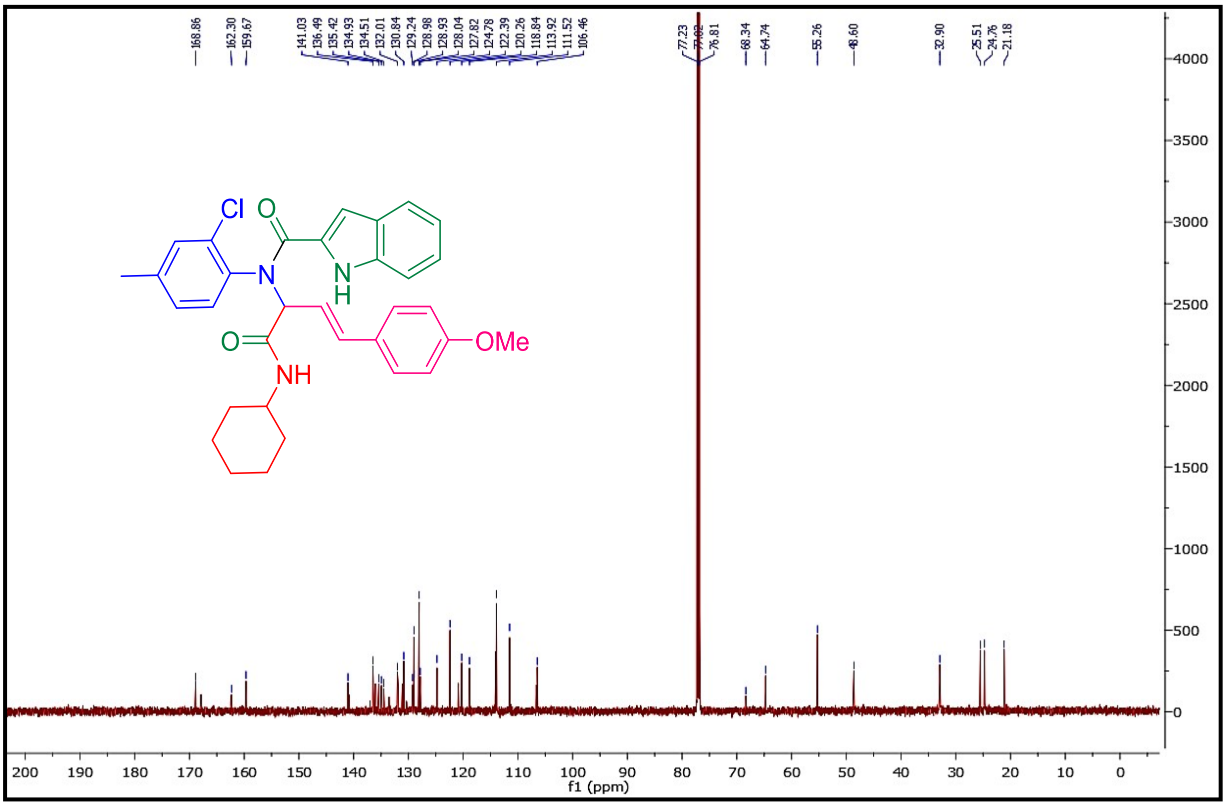

^{1}H-NMR (300 MHz, $CDCl_3$) 5p

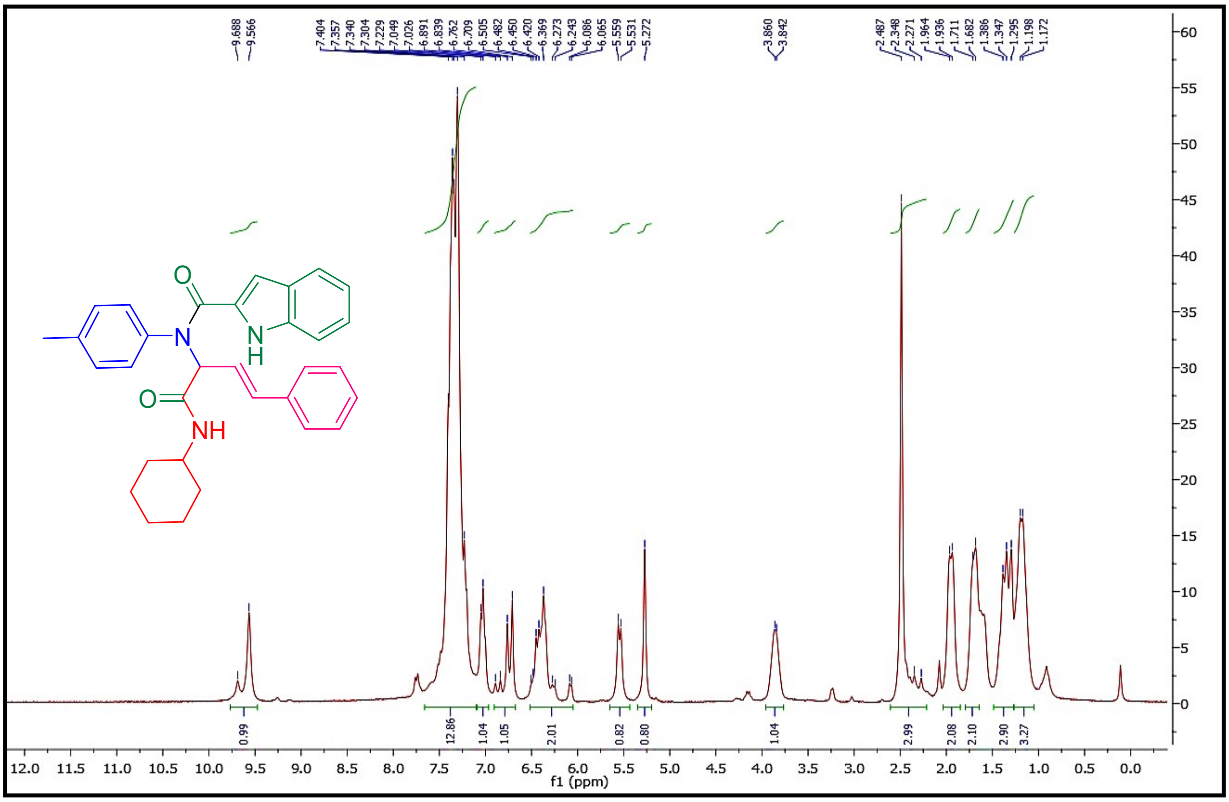

^{13}C-NMR (75 MHz, $CDCl_3$) 5p

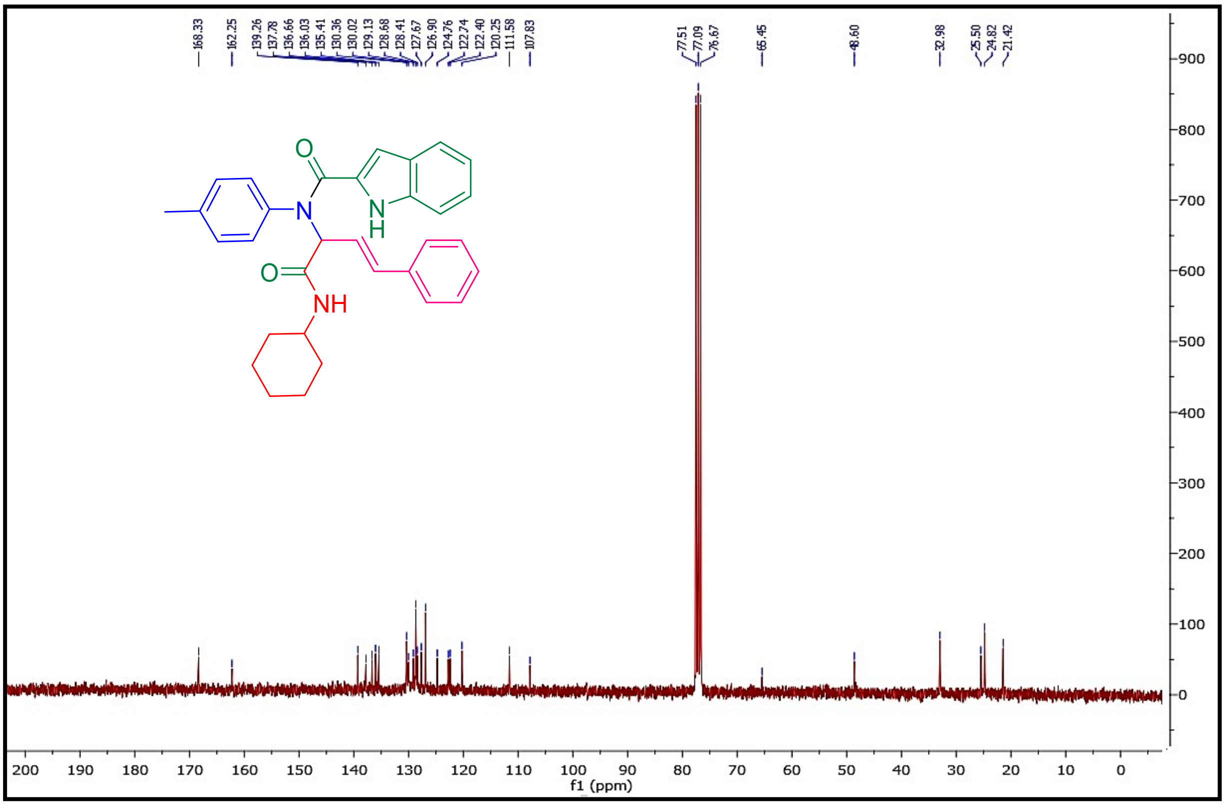

^{1}H-NMR (600 MHz, $CDCl_3$) 5q

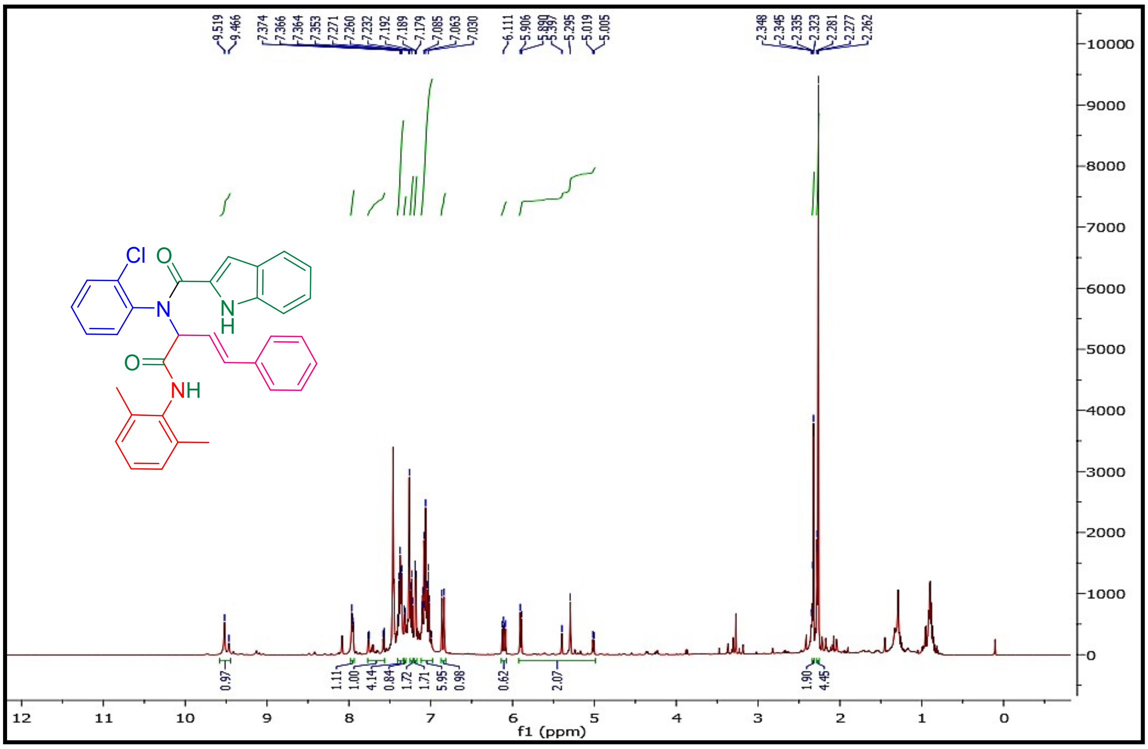

^{13}C-NMR (150 MHz, $CDCl_3$) 5q

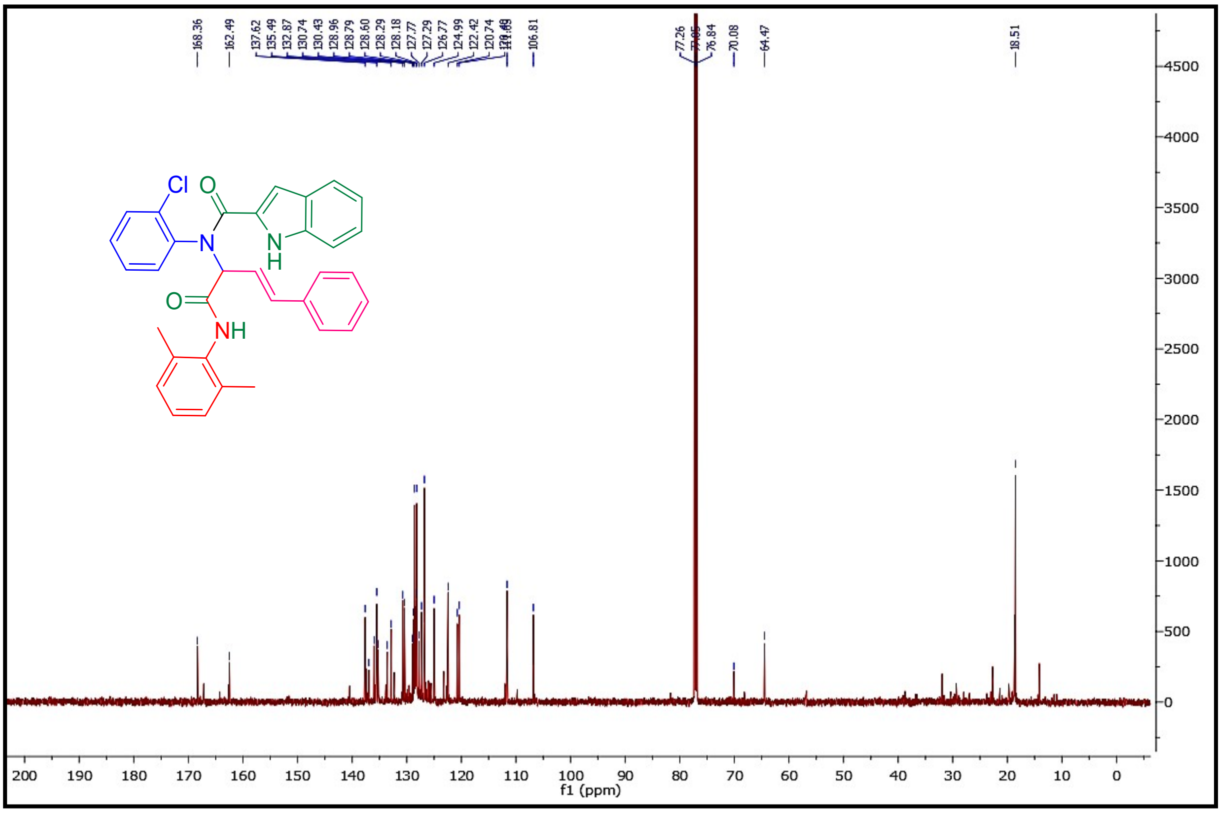

^{1}H-NMR (600 MHz, $CDCl_3$) 5r

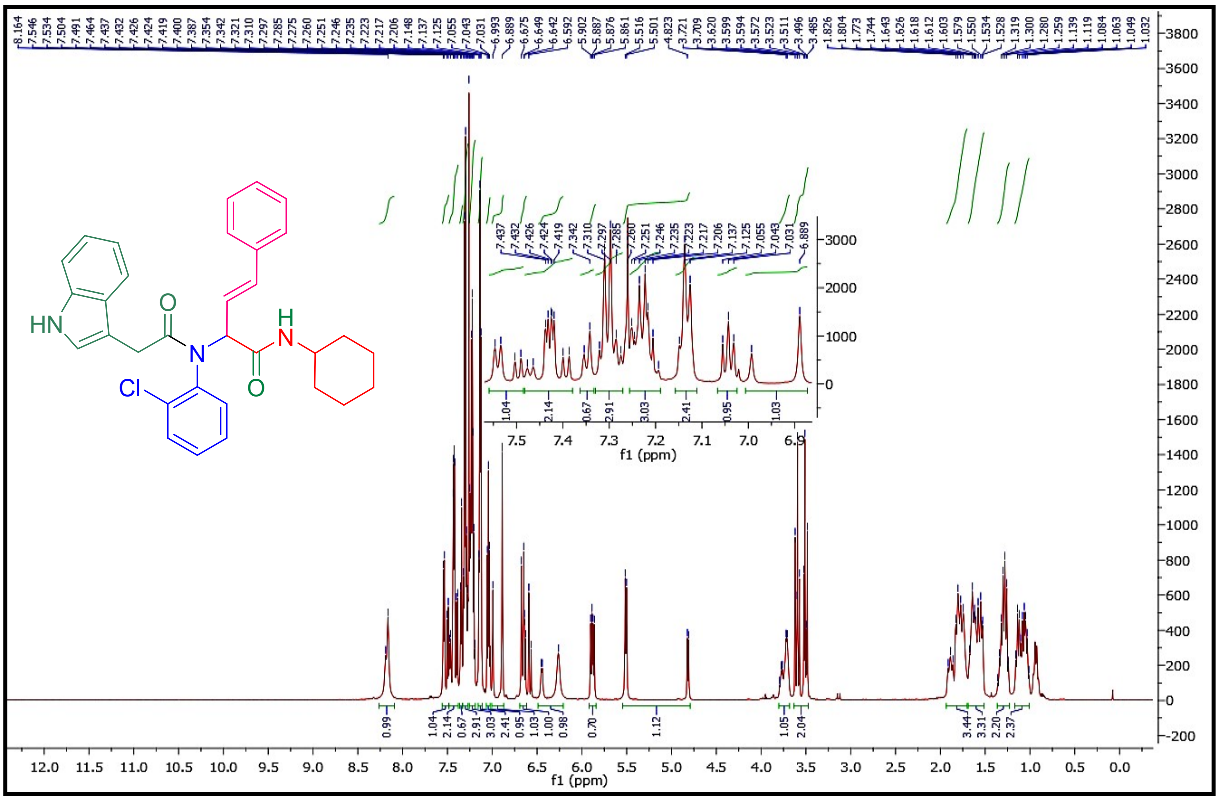

^{13}C-NMR (150 MHz, $CDCl_3$) 5r

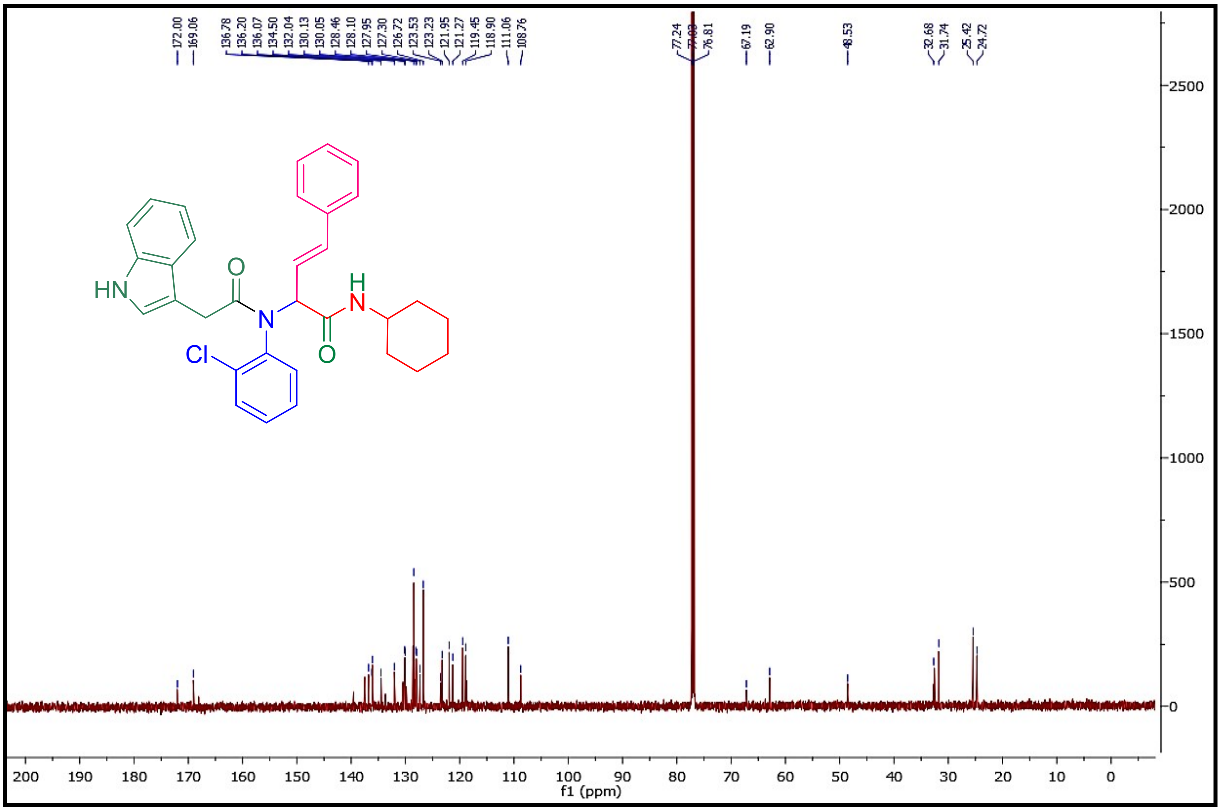

^{1}H-NMR (600 MHz, CDCl$_3$) 5s

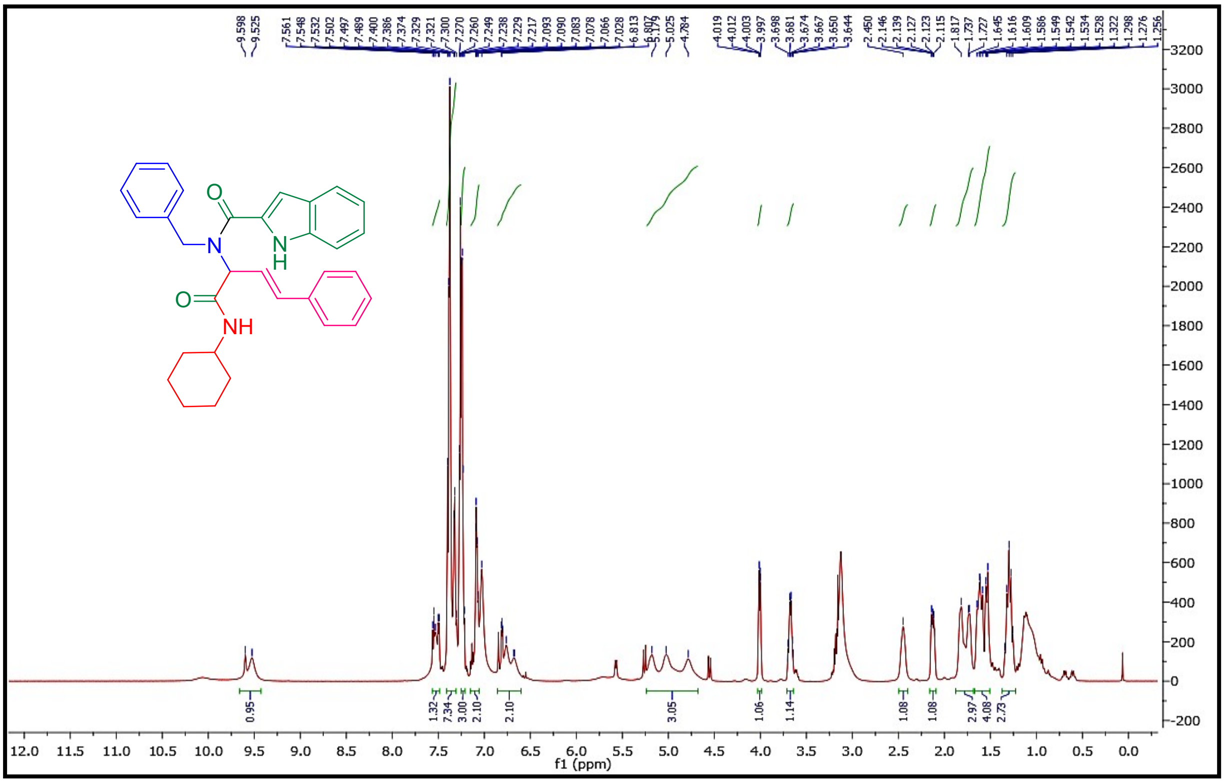

^{13}C-NMR (150 MHz, CDCl$_3$) 5s

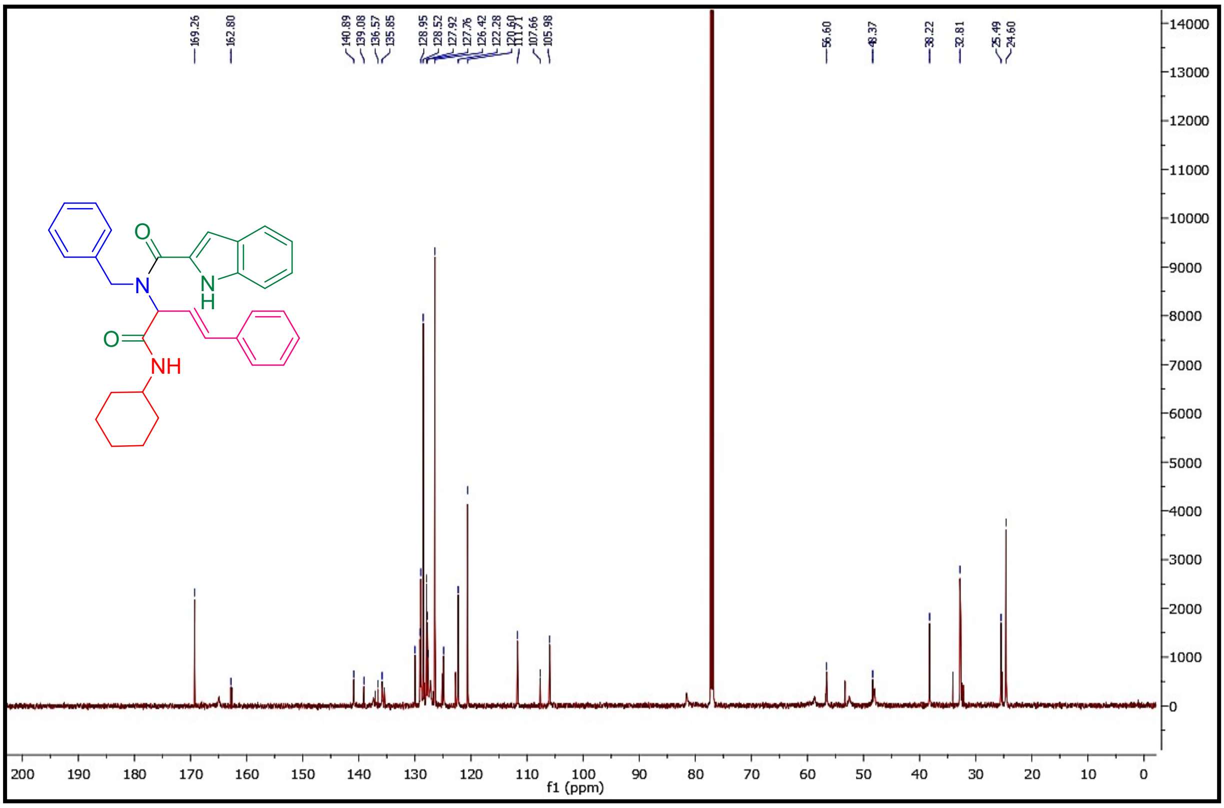

^{1}H-NMR (600 MHz, $CDCl_3$) 6a

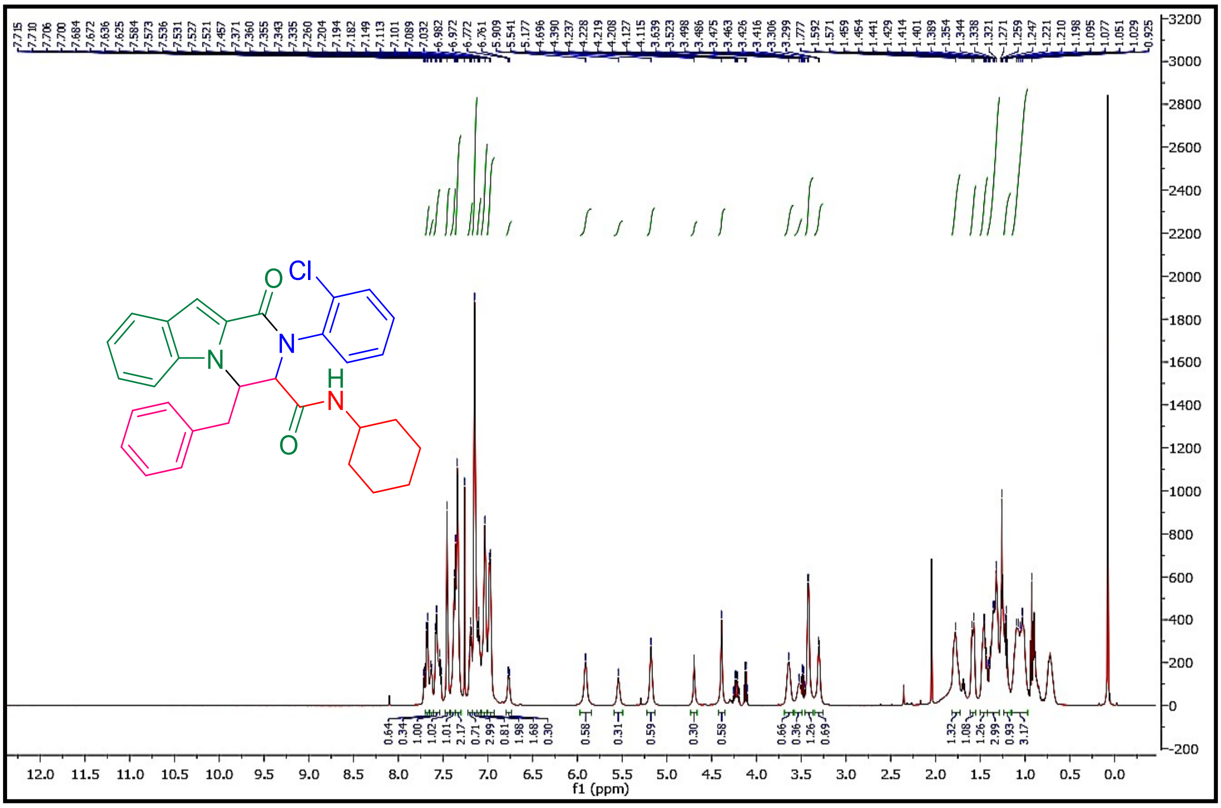

^{13}C-NMR (150 MHz, $CDCl_3$) 6a

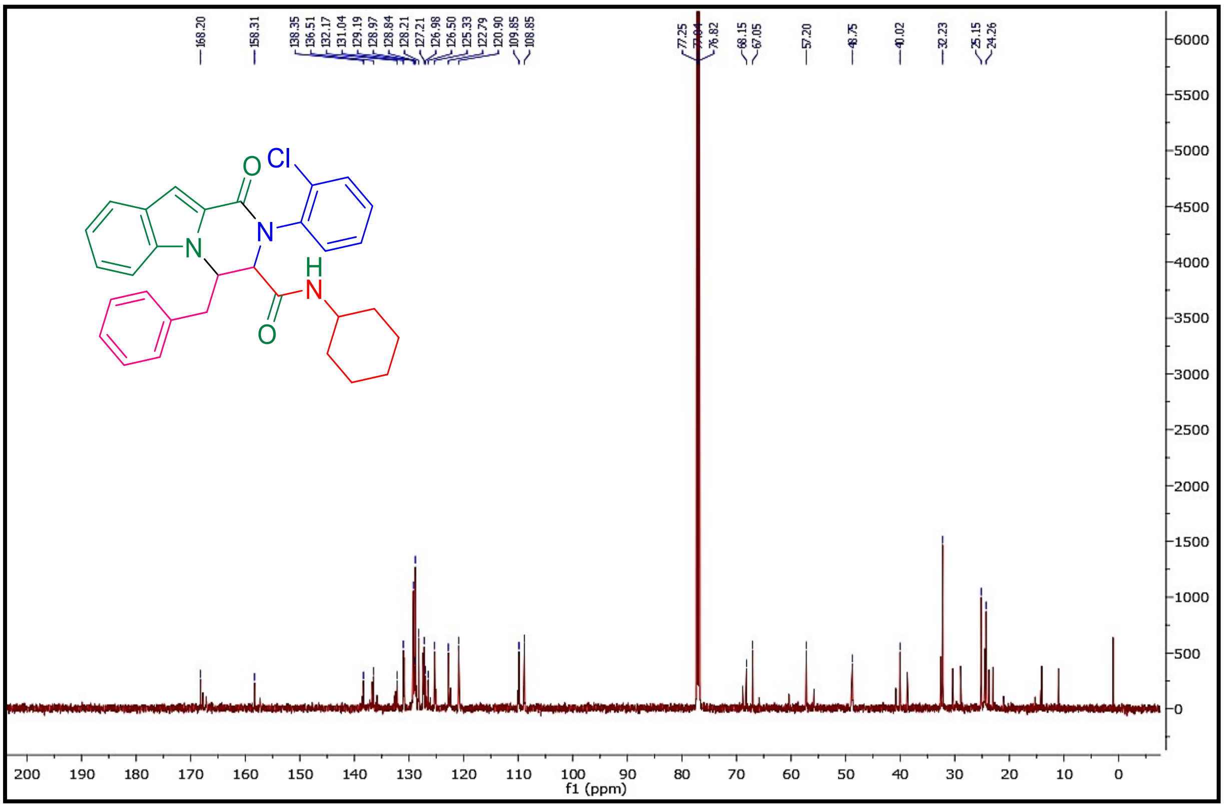

^{1}H-NMR (600 MHz, $CDCl_3$) 6b

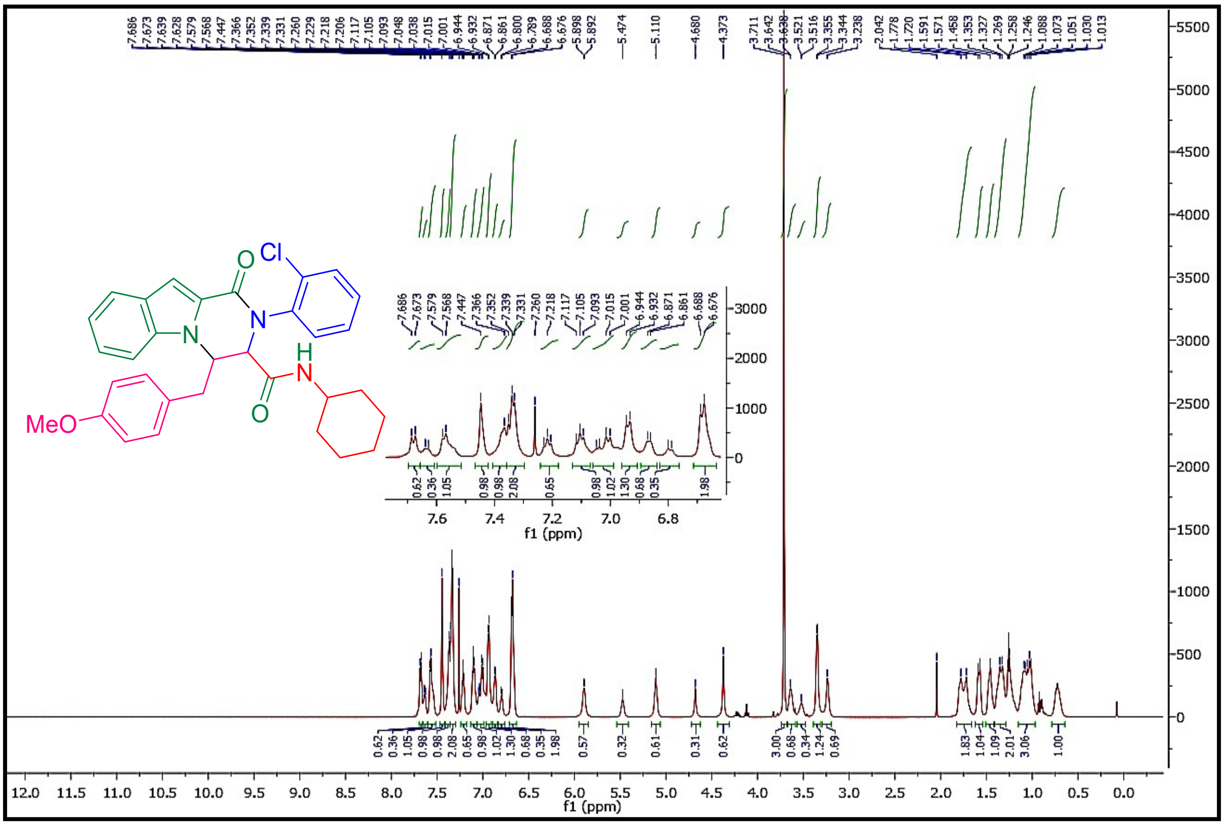

^{13}C-NMR (150 MHz, $CDCl_3$) 6b

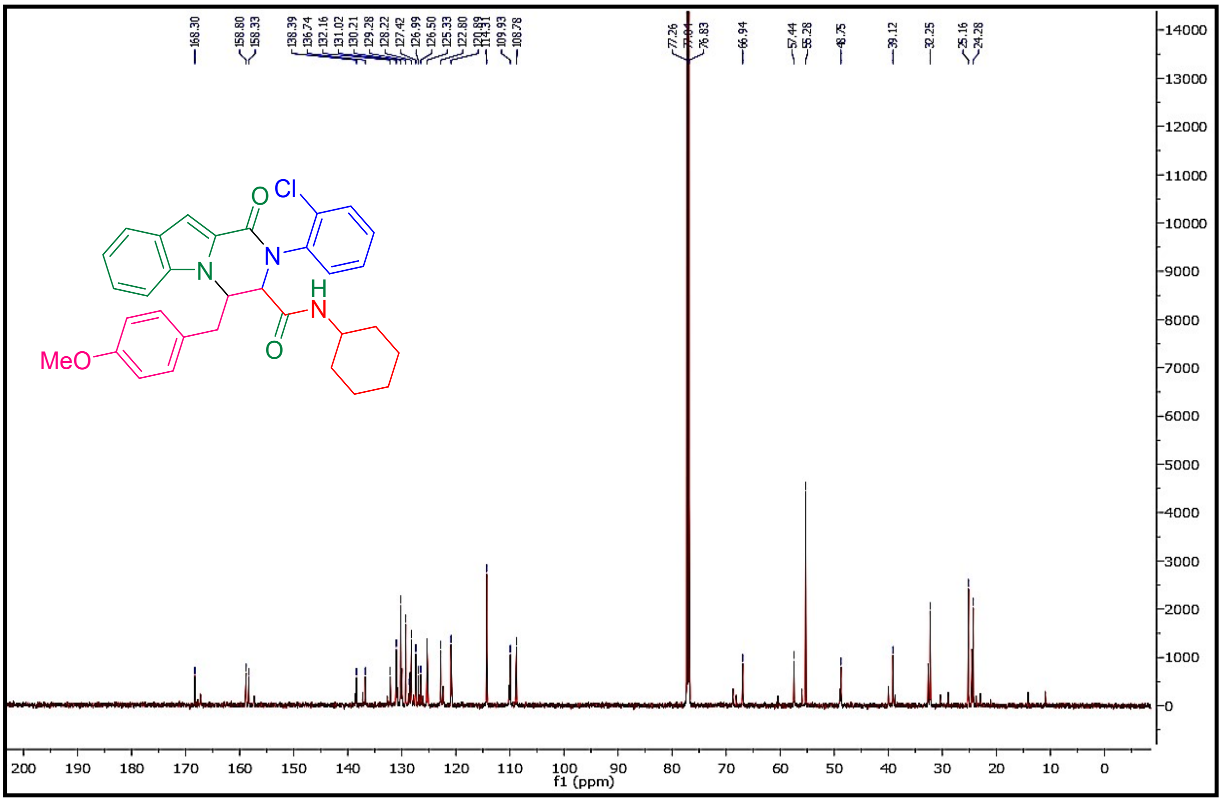

^{1}H-NMR (600 MHz, $CDCl_3$) 6c

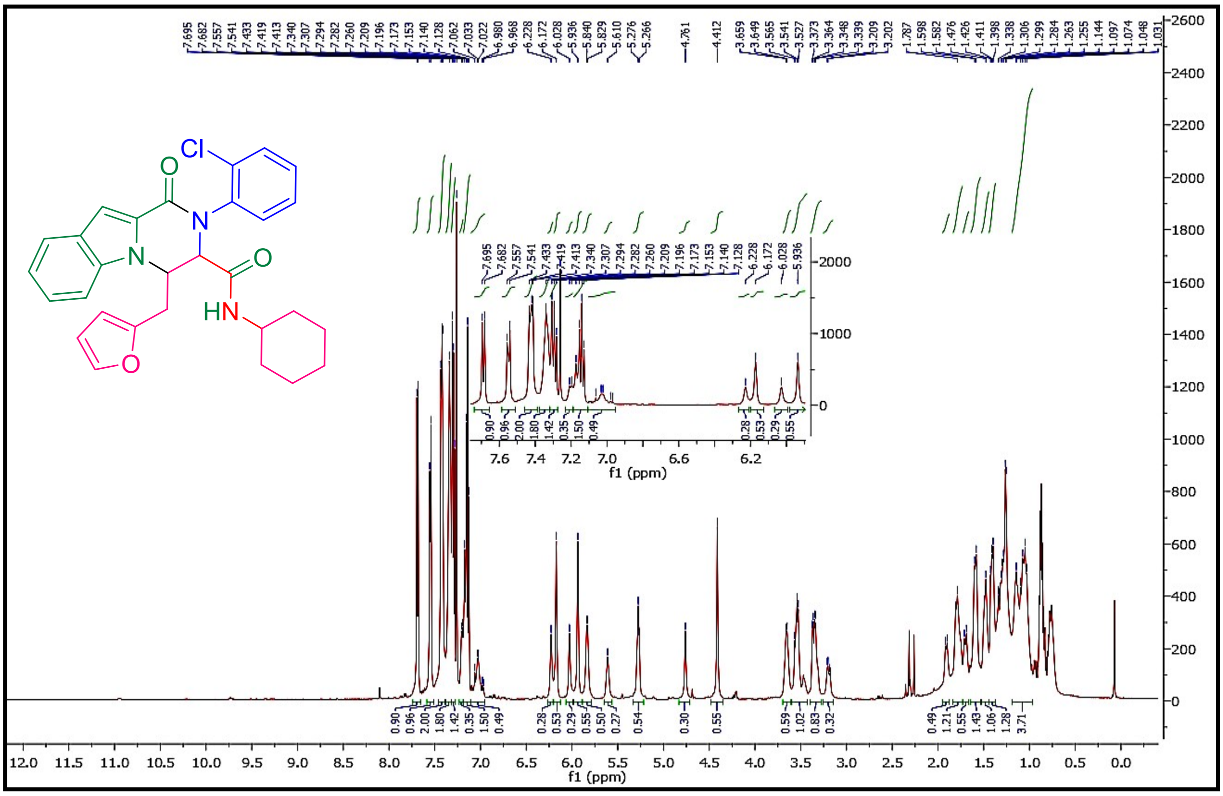

^{13}C-NMR (150MHz, $CDCl_3$) 6c

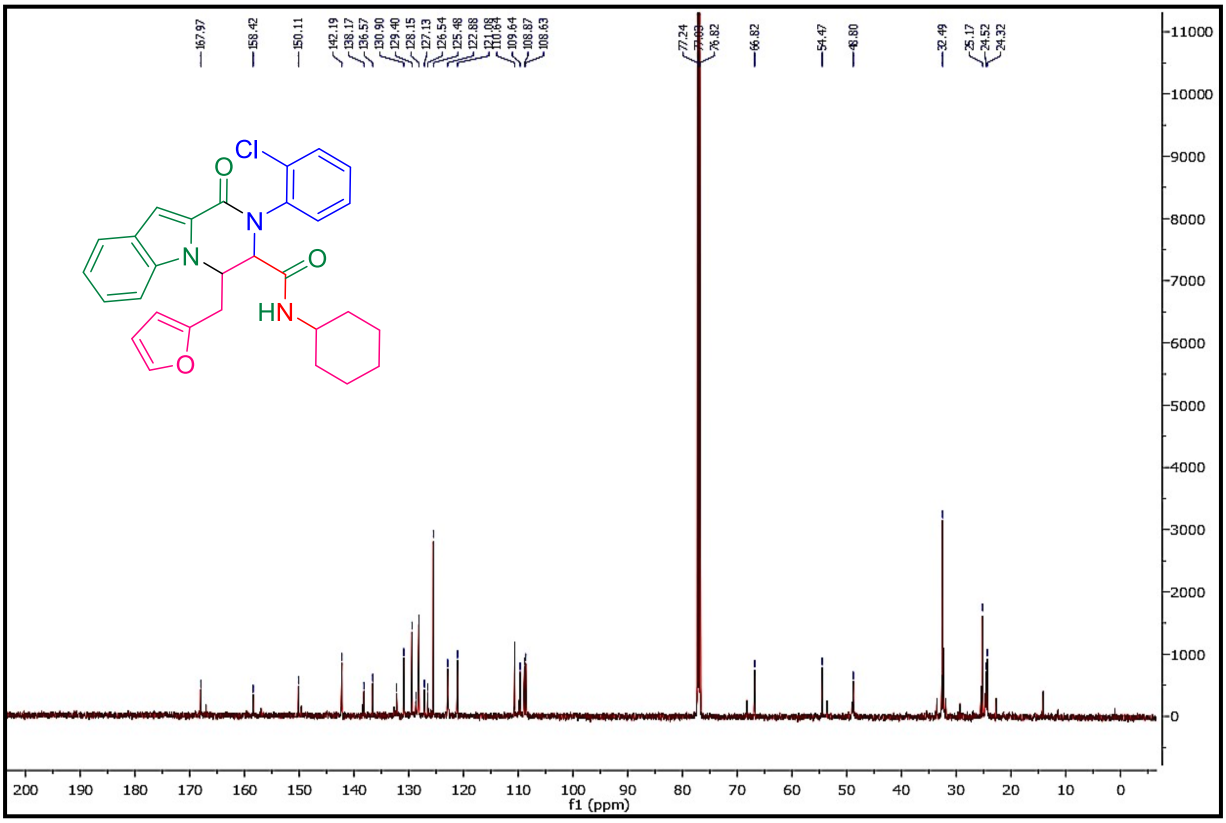

^{1}H-NMR (600 MHz, $CDCl_3$) 6d

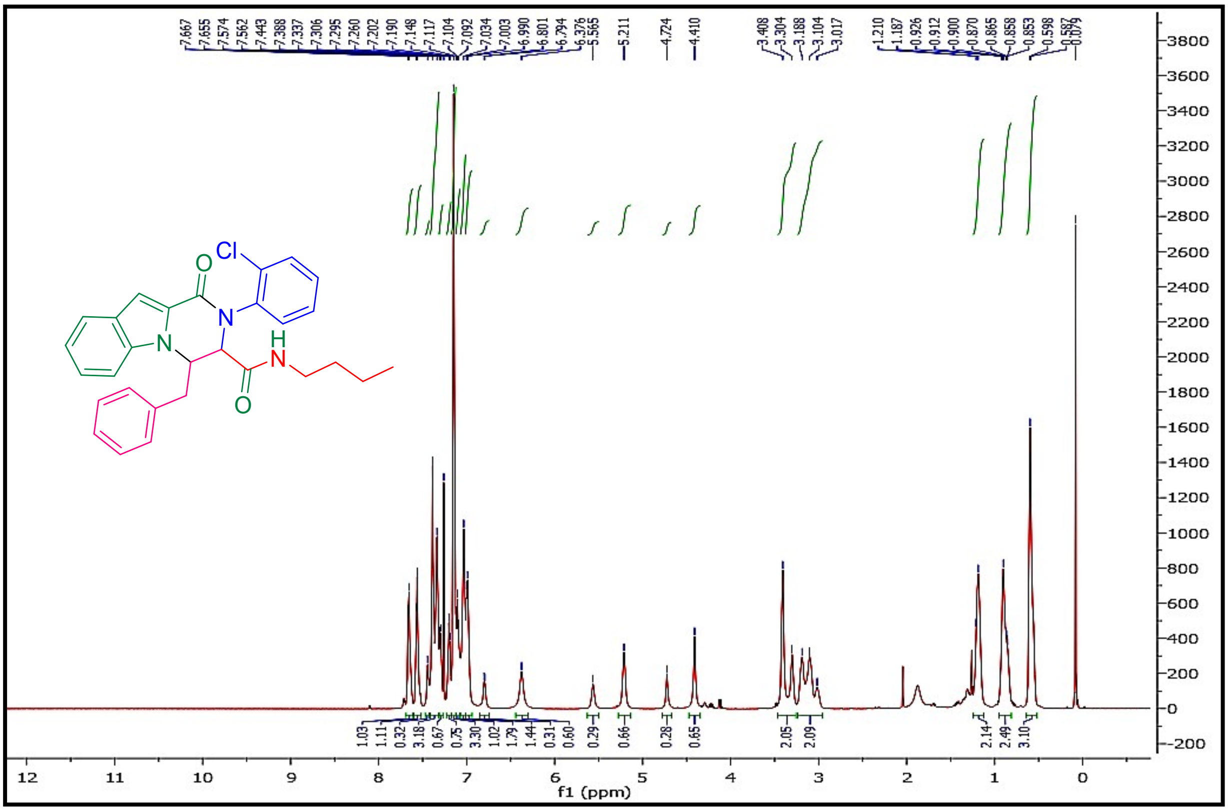

^{13}C-NMR (150 MHz, $CDCl_3$) 6d

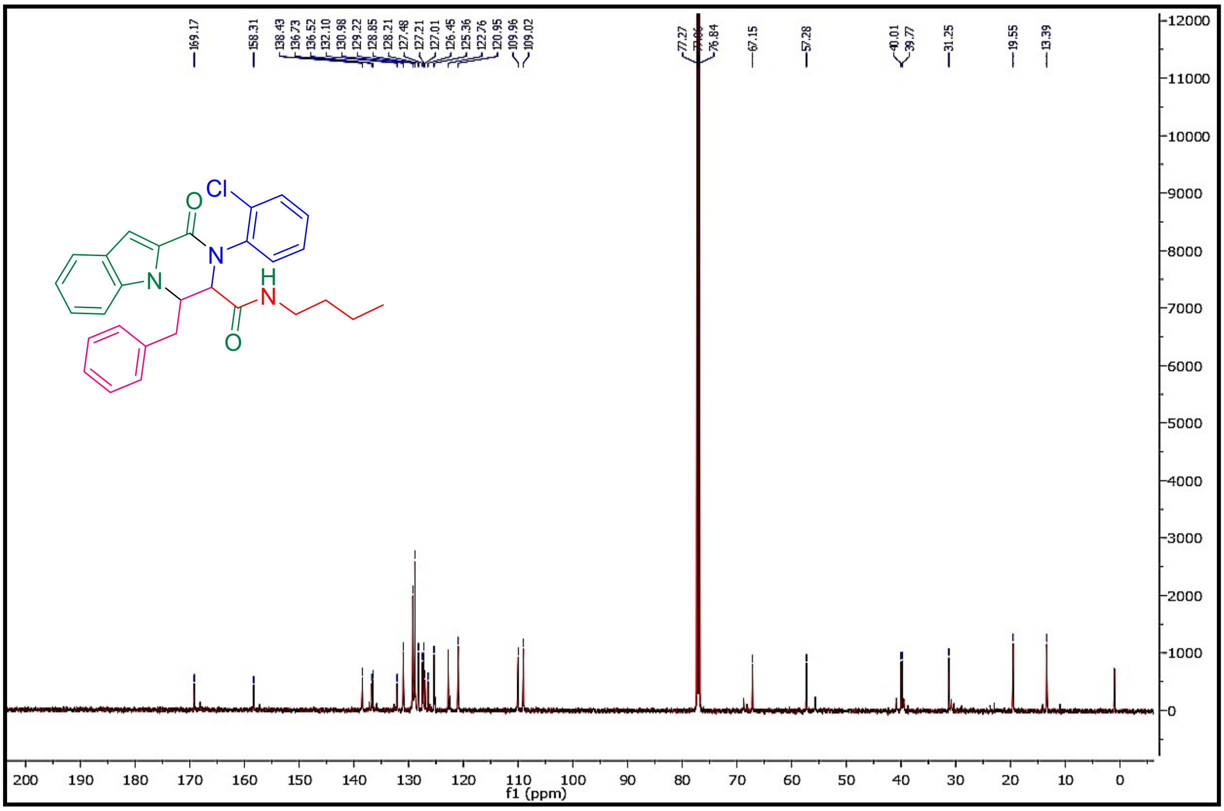

^{1}H-NMR (600 MHz, $CDCl_3$) 6e

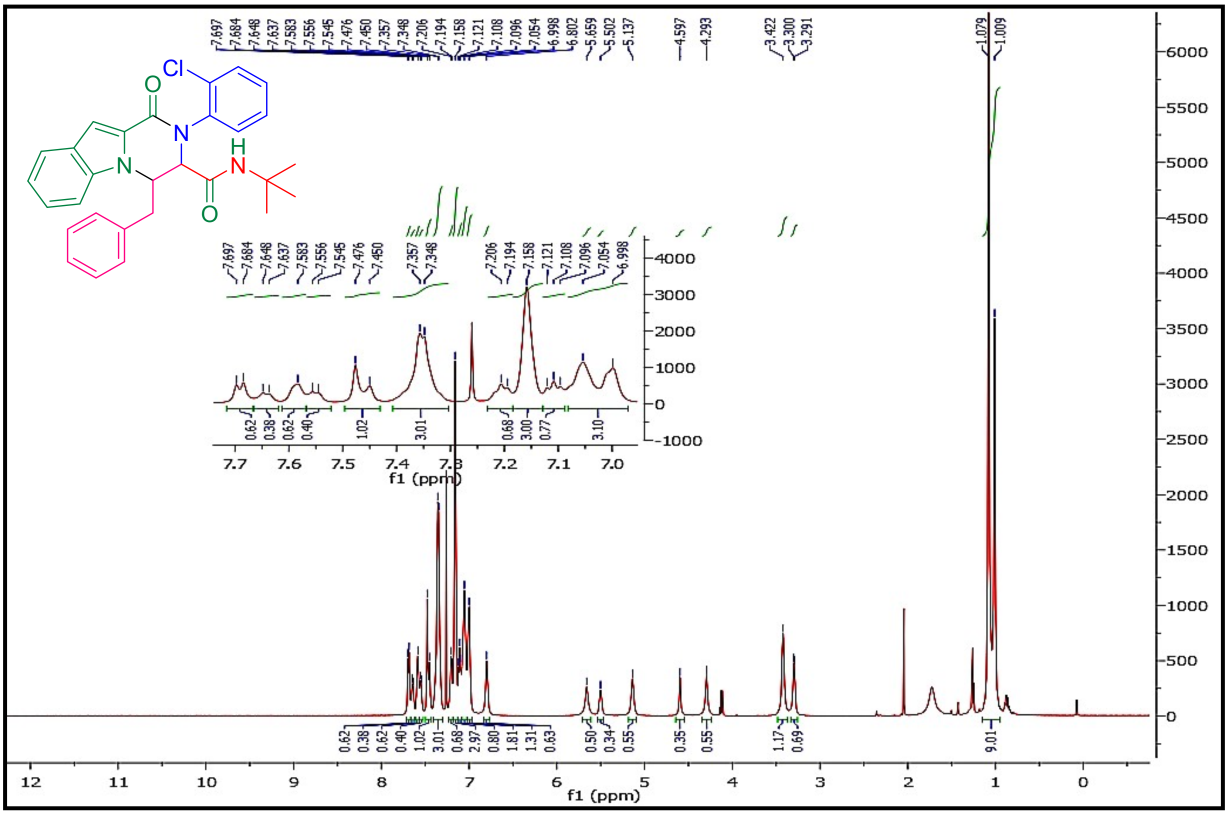

^{13}C-NMR (150 MHz, $CDCl_3$) 6e

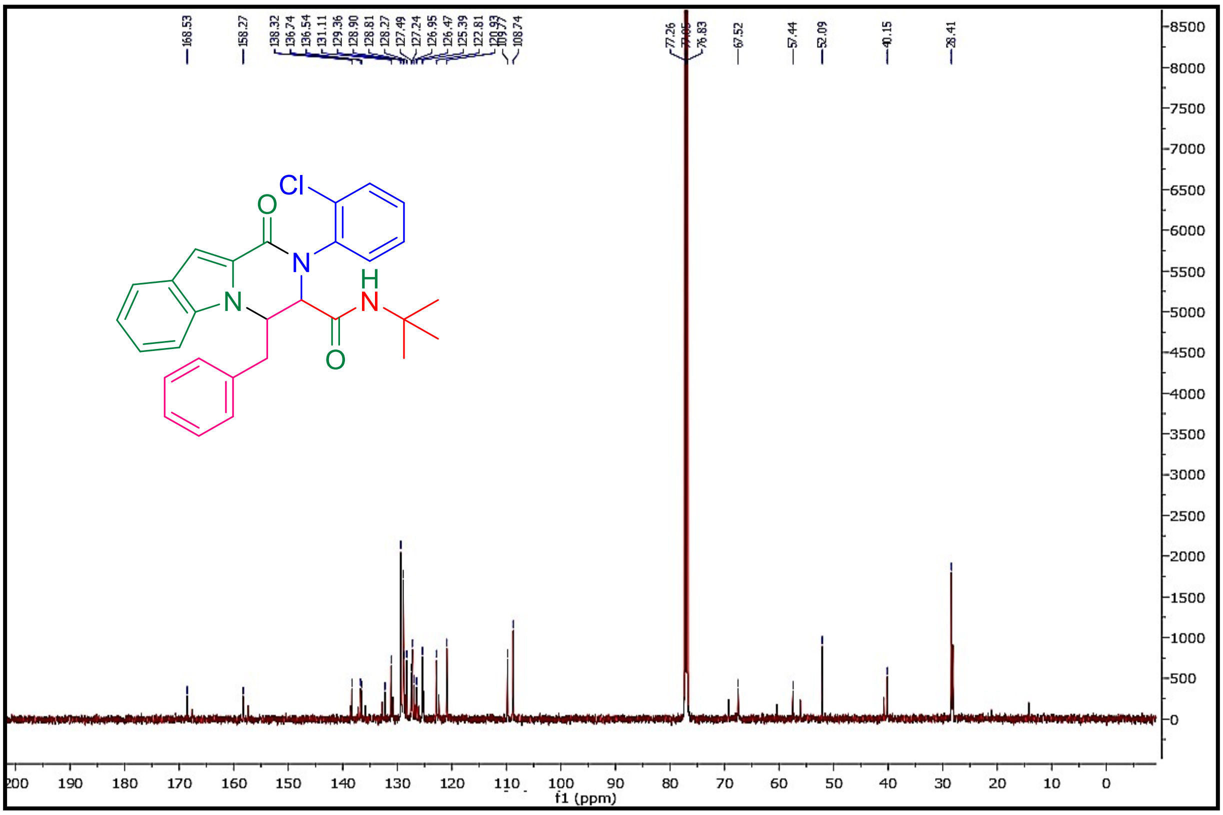

^{1}H-NMR (600 MHz, $CDCl_3$) 6f

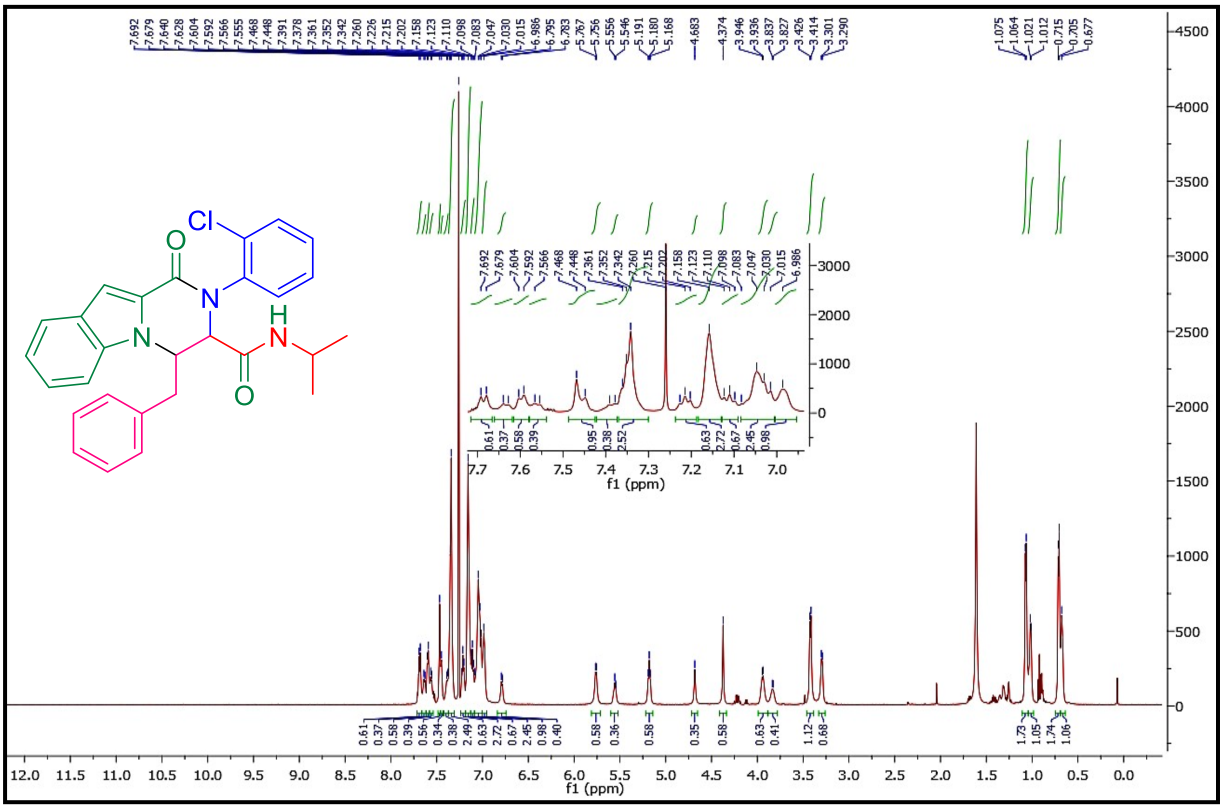

^{13}C-NMR (150 MHz, $CDCl_3$) 6f

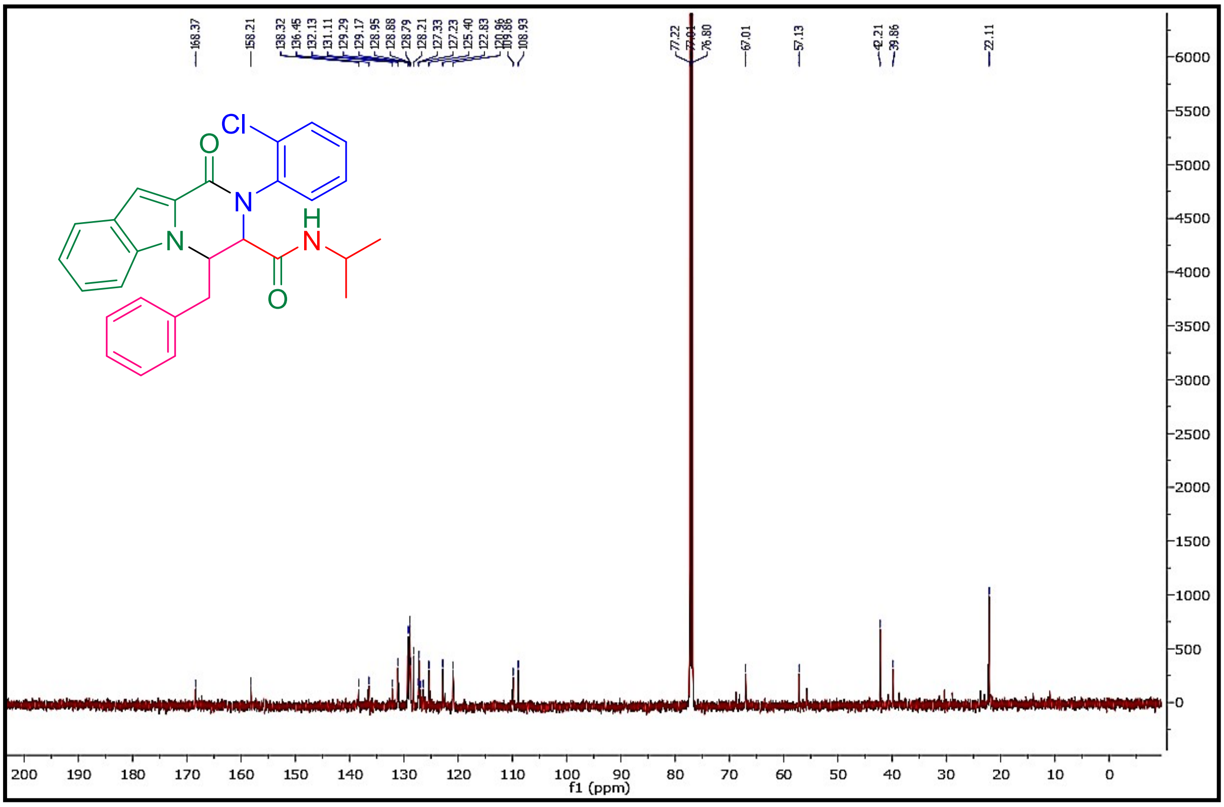

^{1}H-NMR (600 MHz, $CDCl_3$) 6g

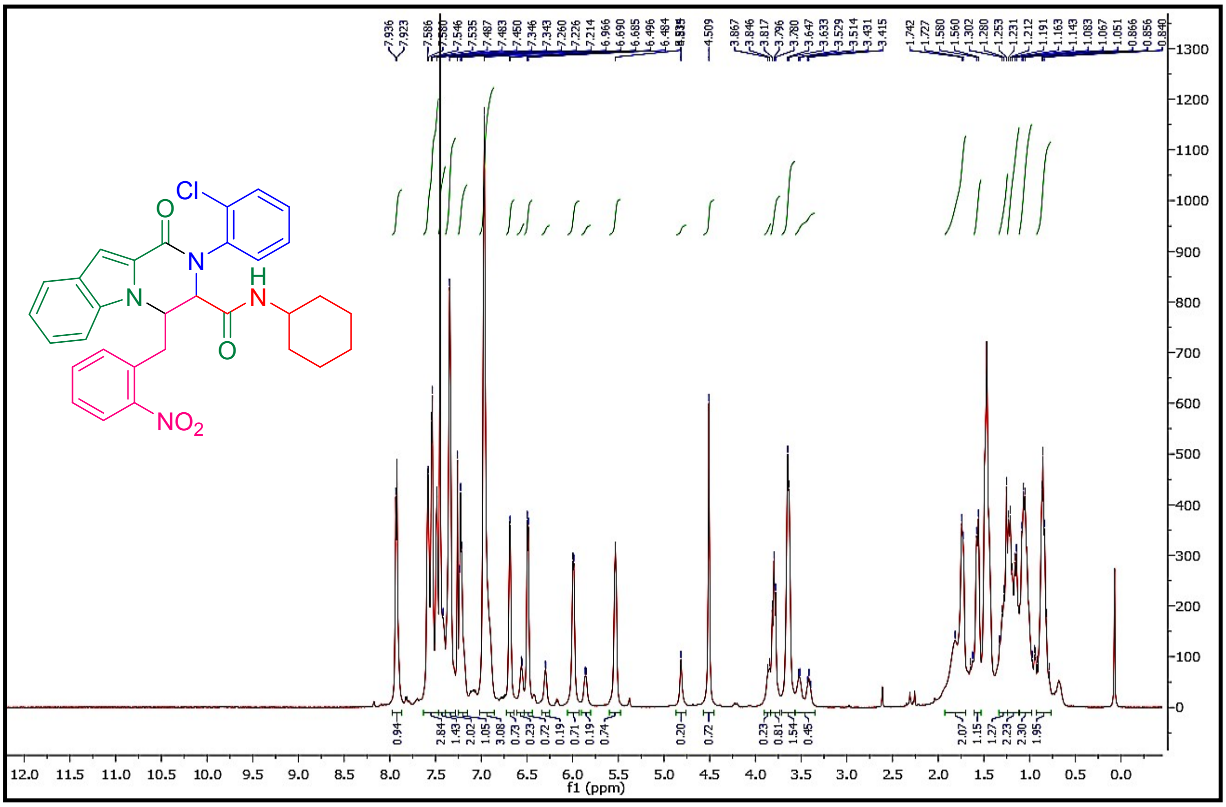

^{13}C-NMR (150 MHz, $CDCl_3$) 6g

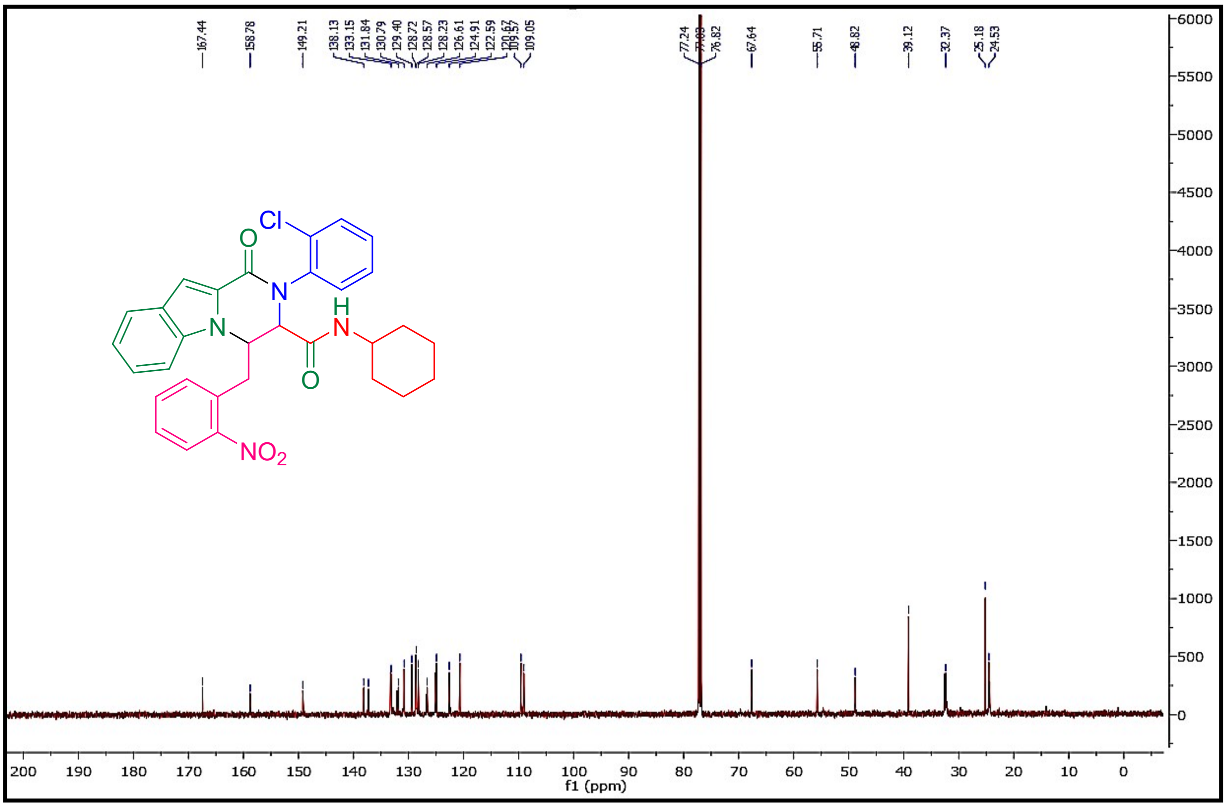

^{1}H-NMR (600 MHz, $CDCl_3$) 6h

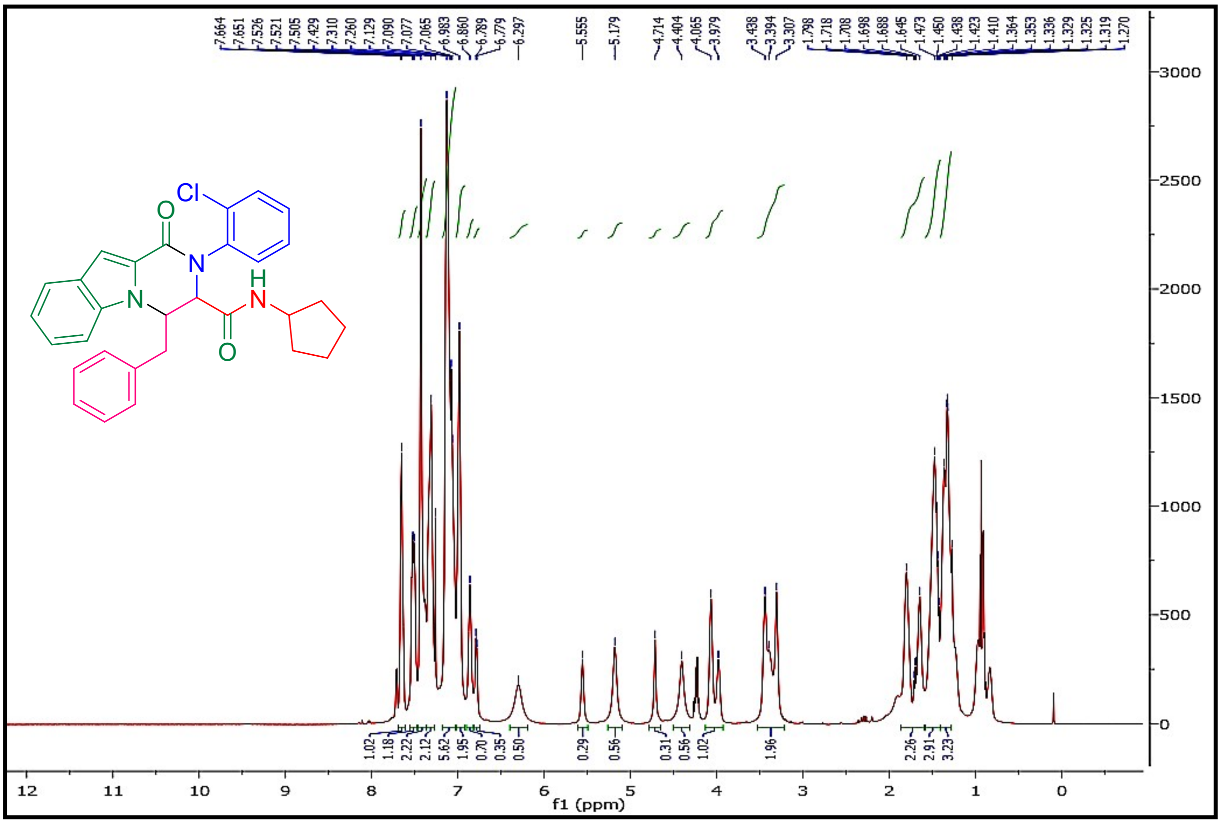

^{1}H-NMR (600 MHz, $CDCl_3$) 6h

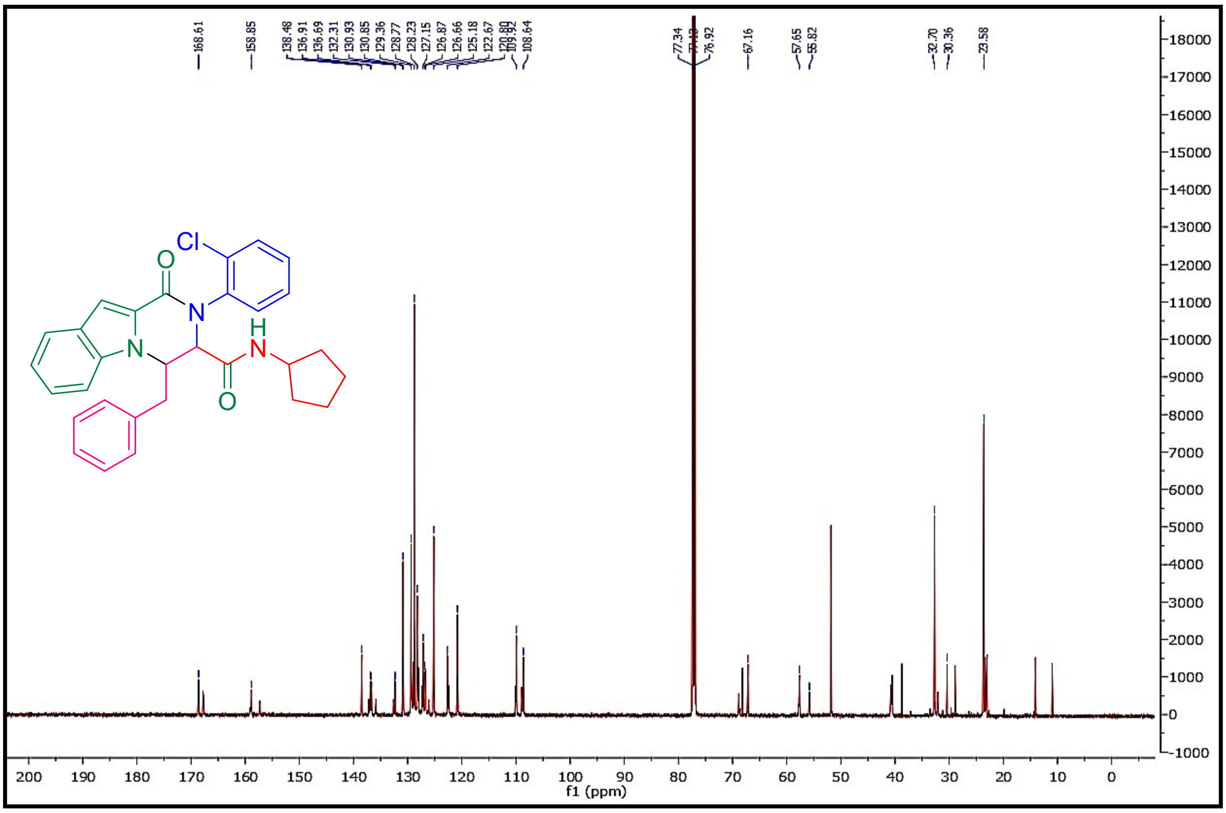

^{1}H-NMR (600 MHz, $CDCl_3$) 6i

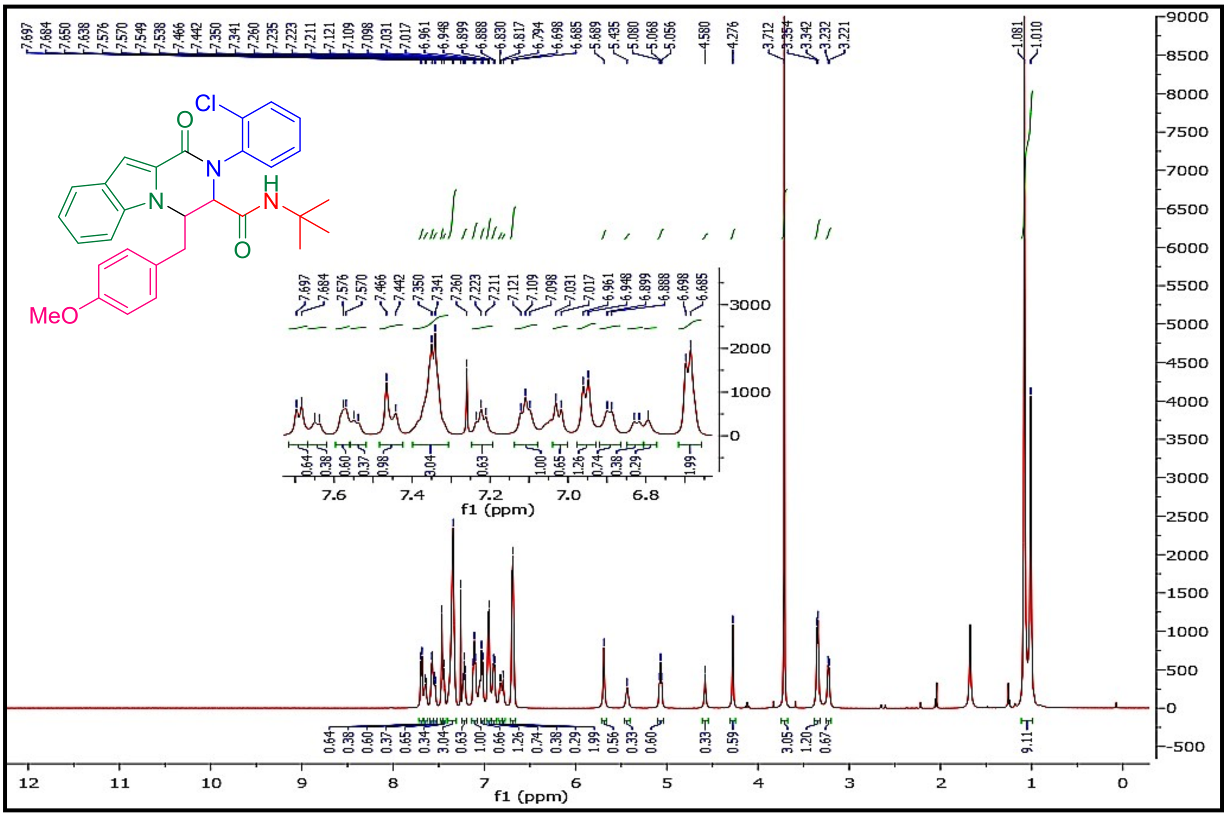

^{13}C-NMR (150 MHz, $CDCl_3$) 6i

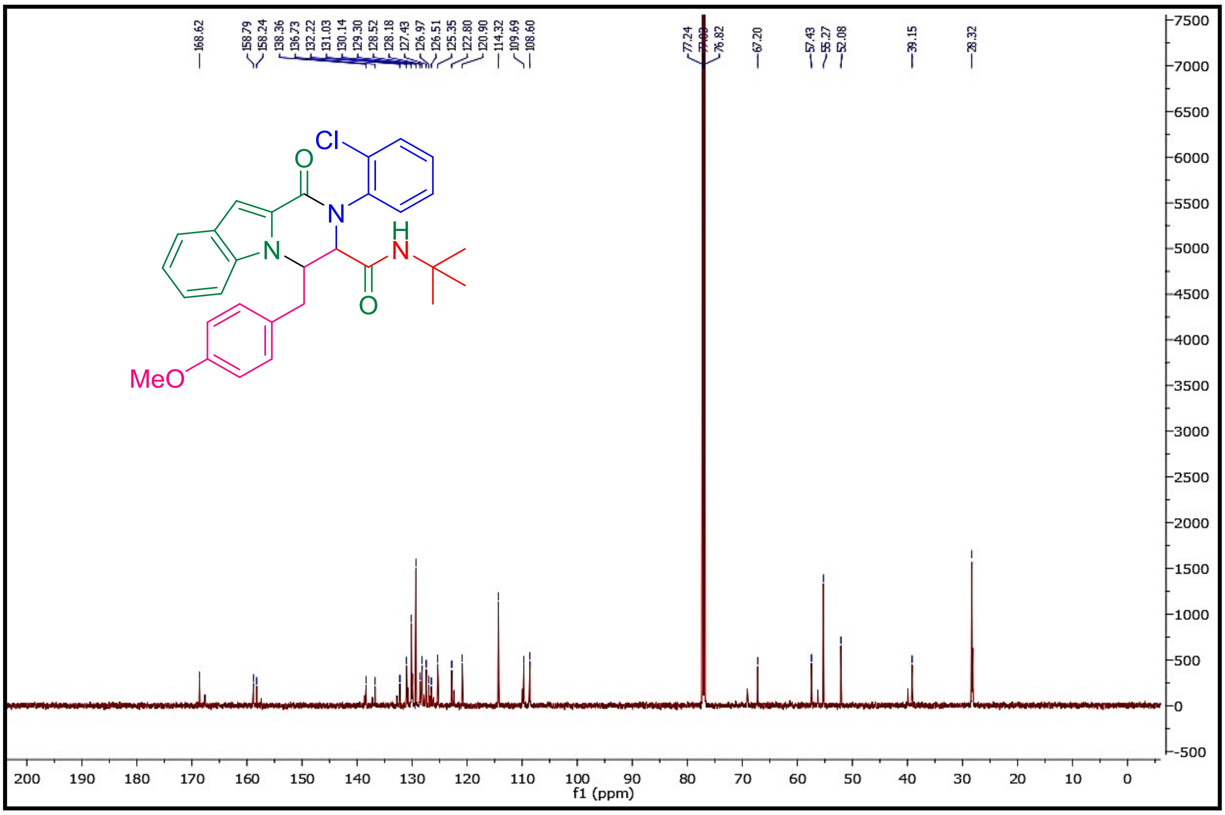

^{1}H-NMR (600 MHz, $CDCl_3$) 6j

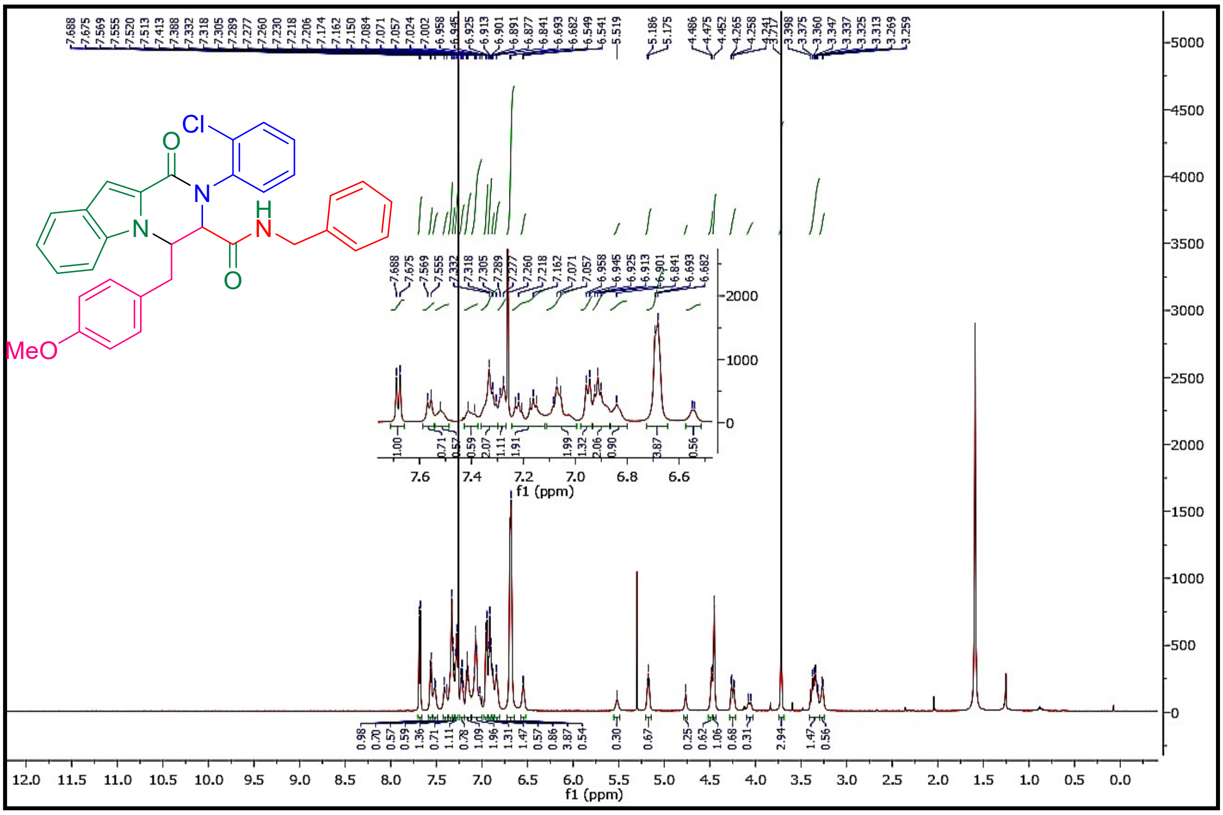

^{13}C-NMR (150 MHz, $CDCl_3$) 6j

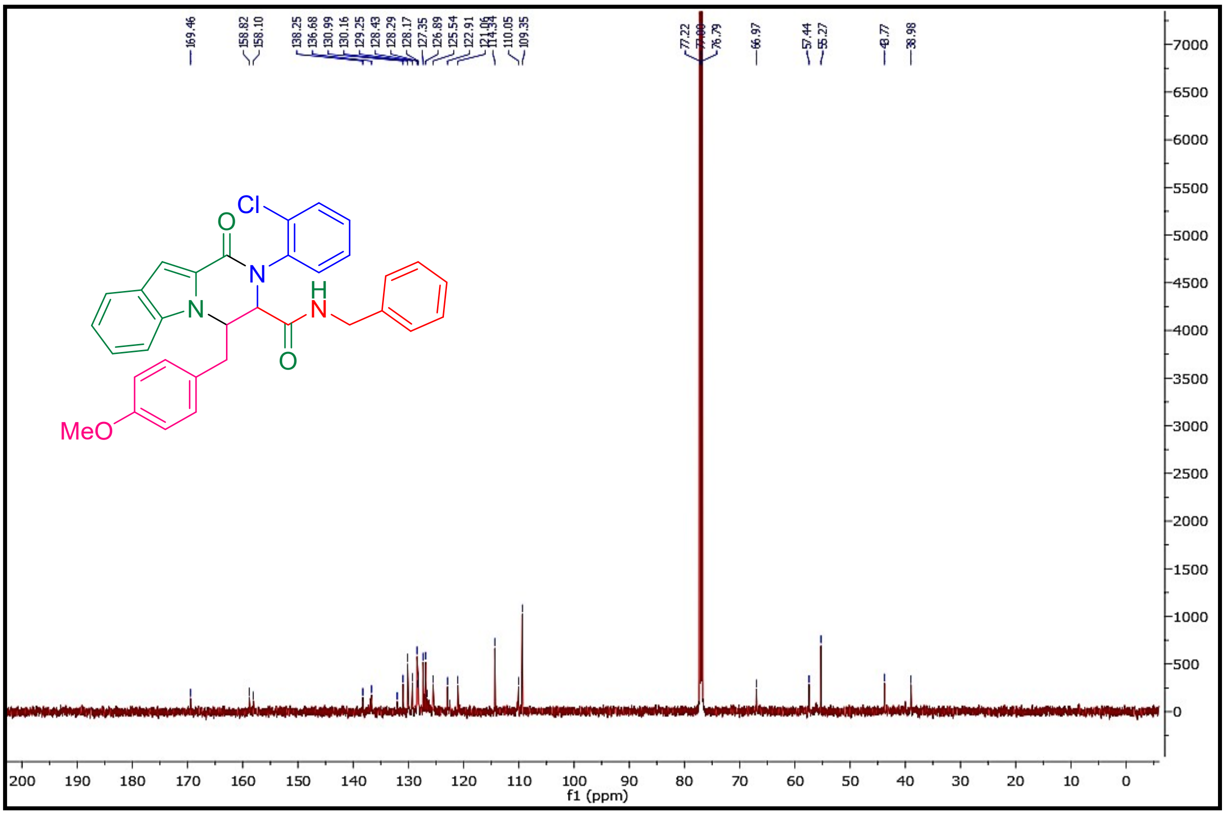

^{1}H-NMR (600 MHz, $CDCl_3$) 6k

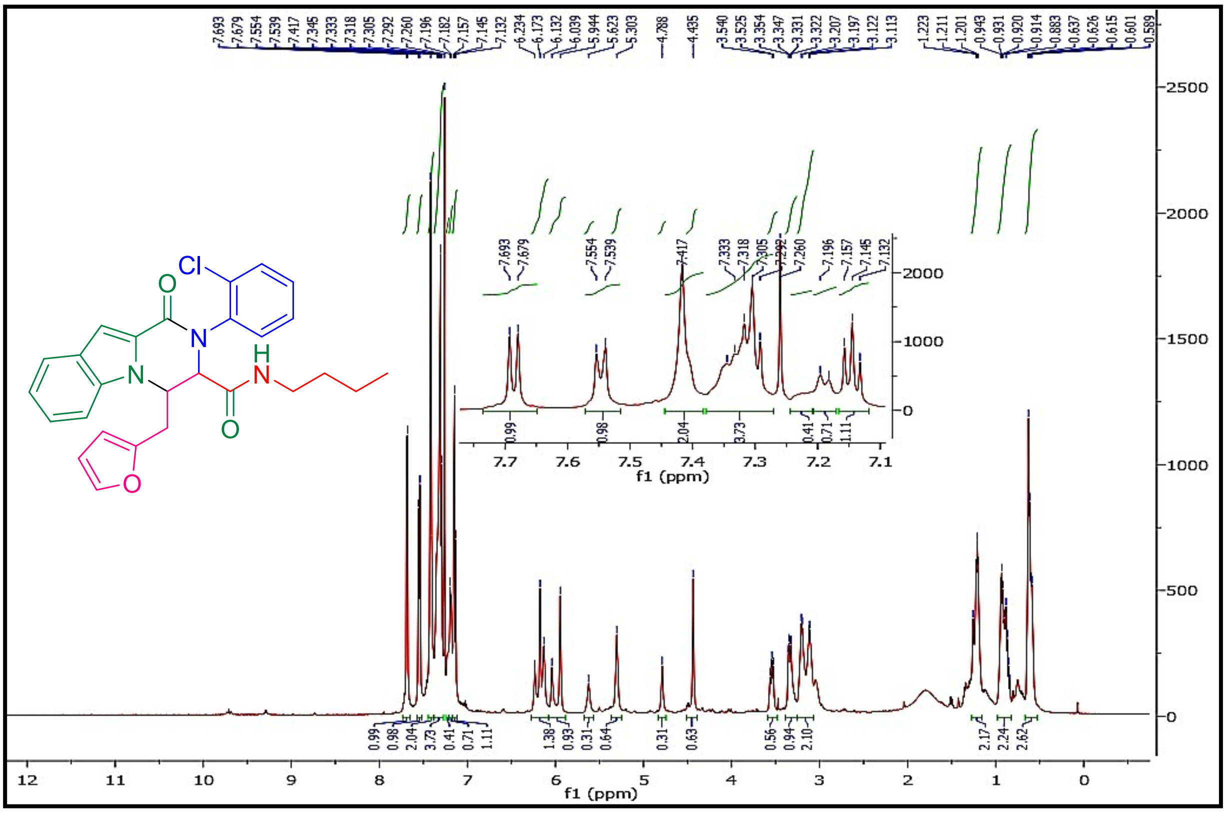

^{13}C-NMR (150 MHz, $CDCl_3$) 6k

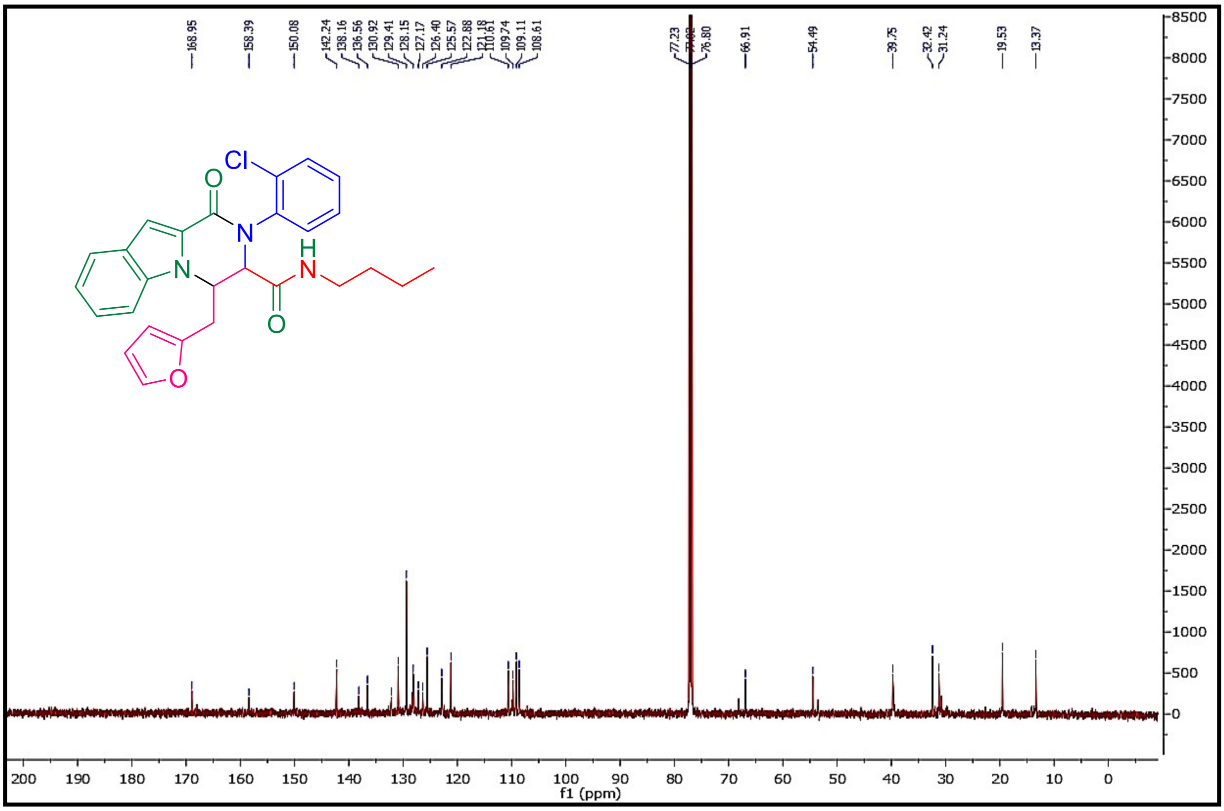

^{1}H-NMR (600 MHz, $CDCl_3$) 6l

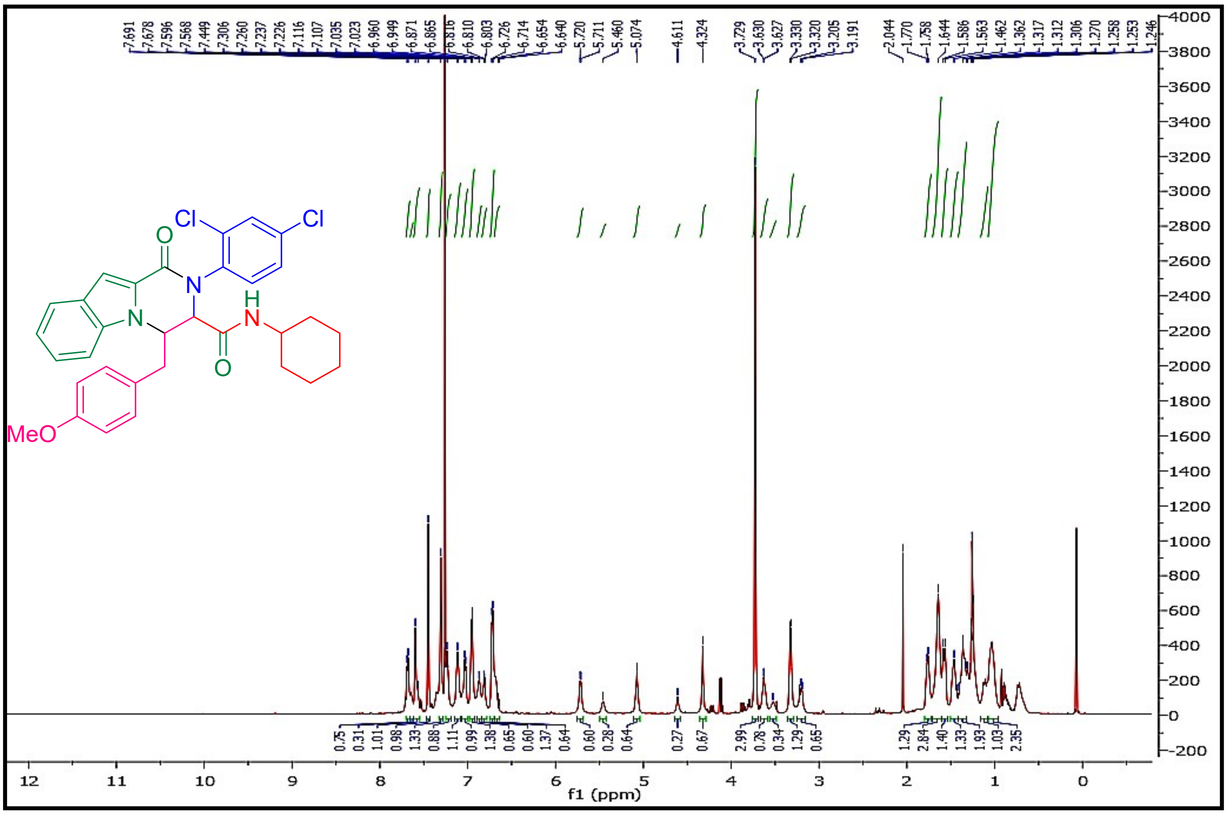

^{13}C-NMR (150 MHz, $CDCl_3$) 6l

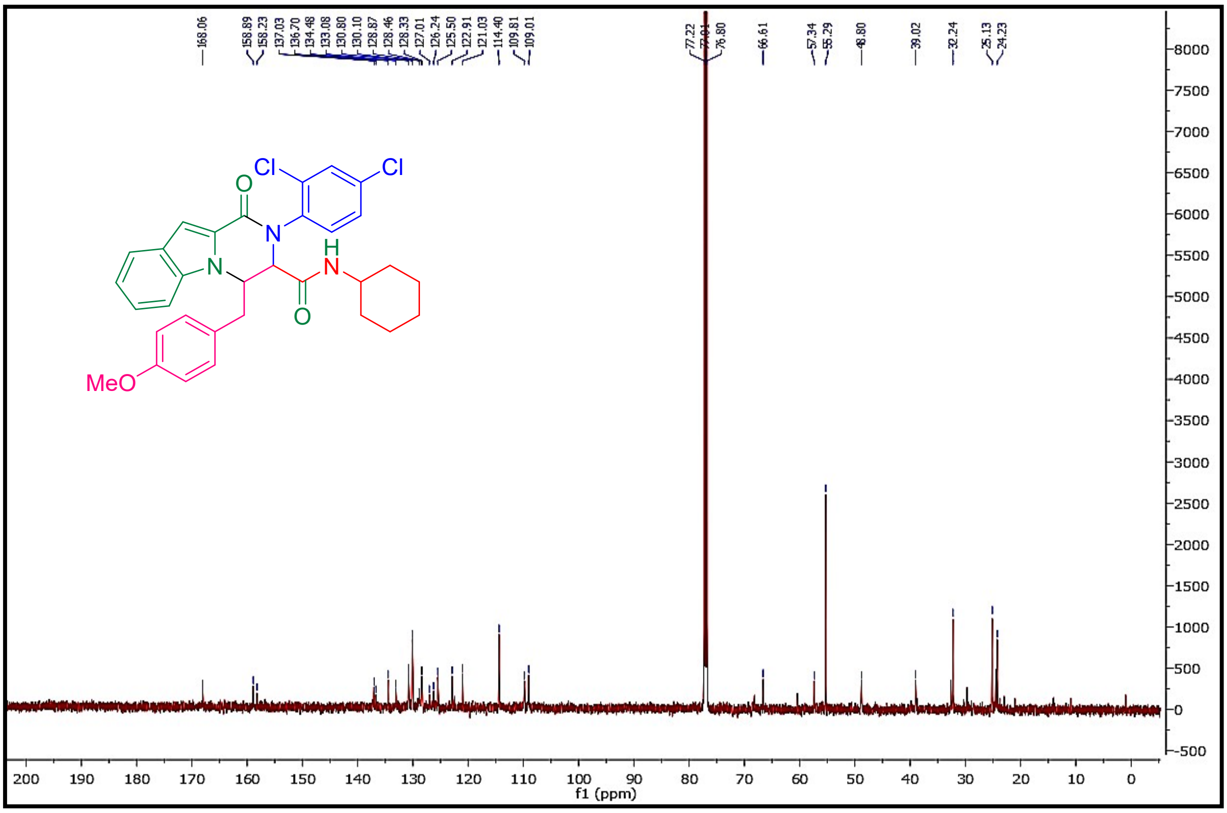

^{1}H-NMR (600 MHz, DMSO-*d6*) 6m

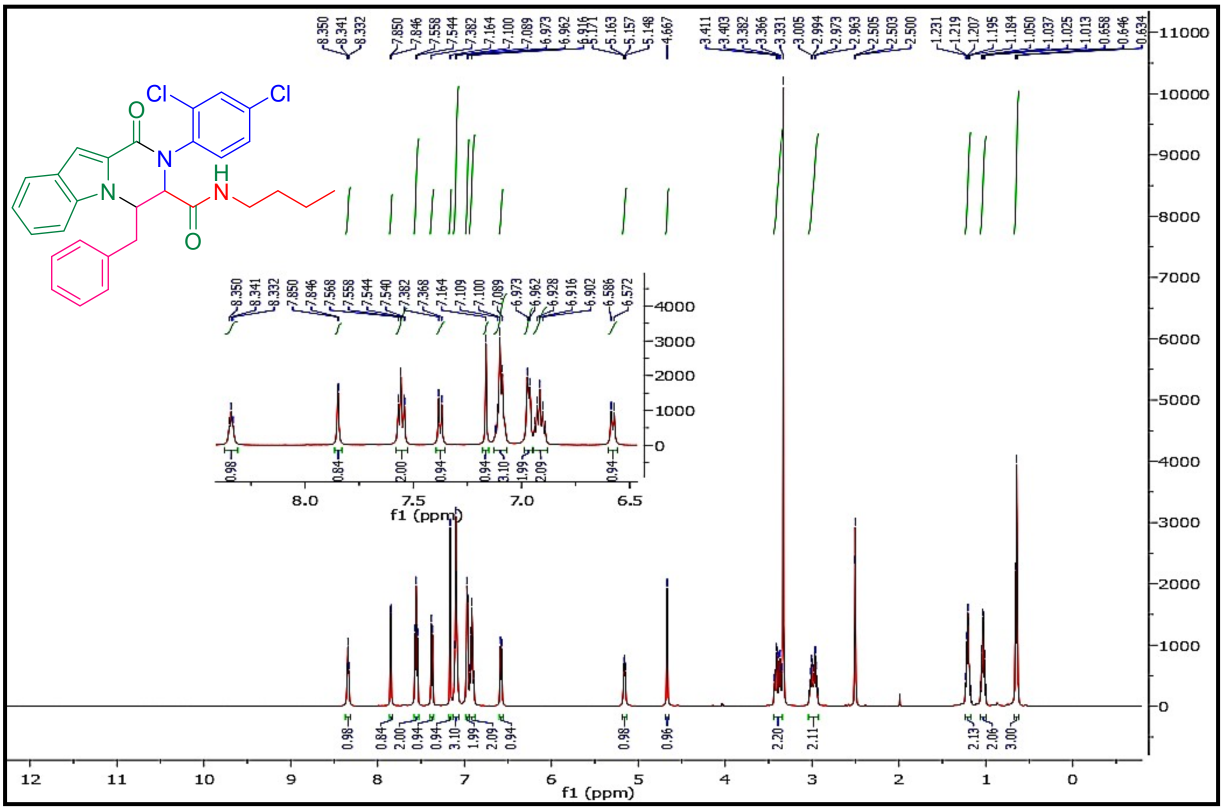

^{13}C-NMR (150 MHz, DMSO-*d6*) 6m

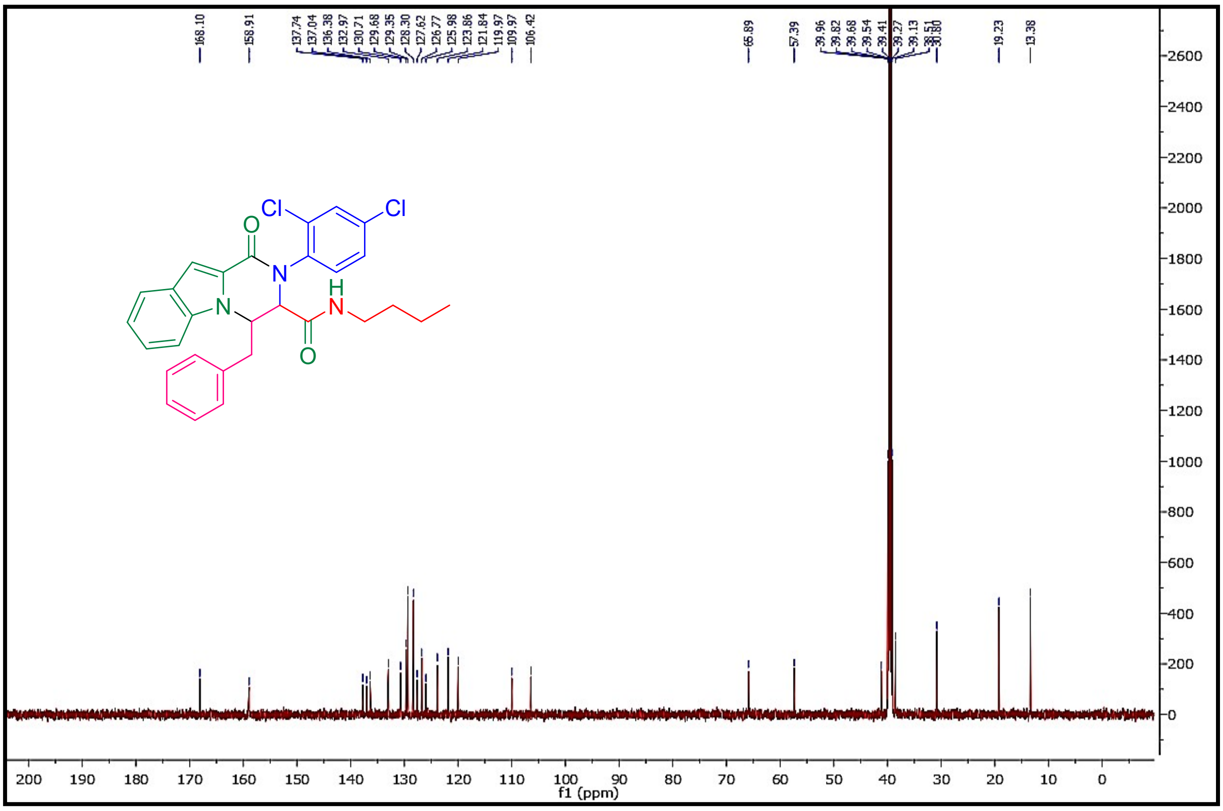

^{1}H-NMR (600 MHz, $CDCl_3$) 6n

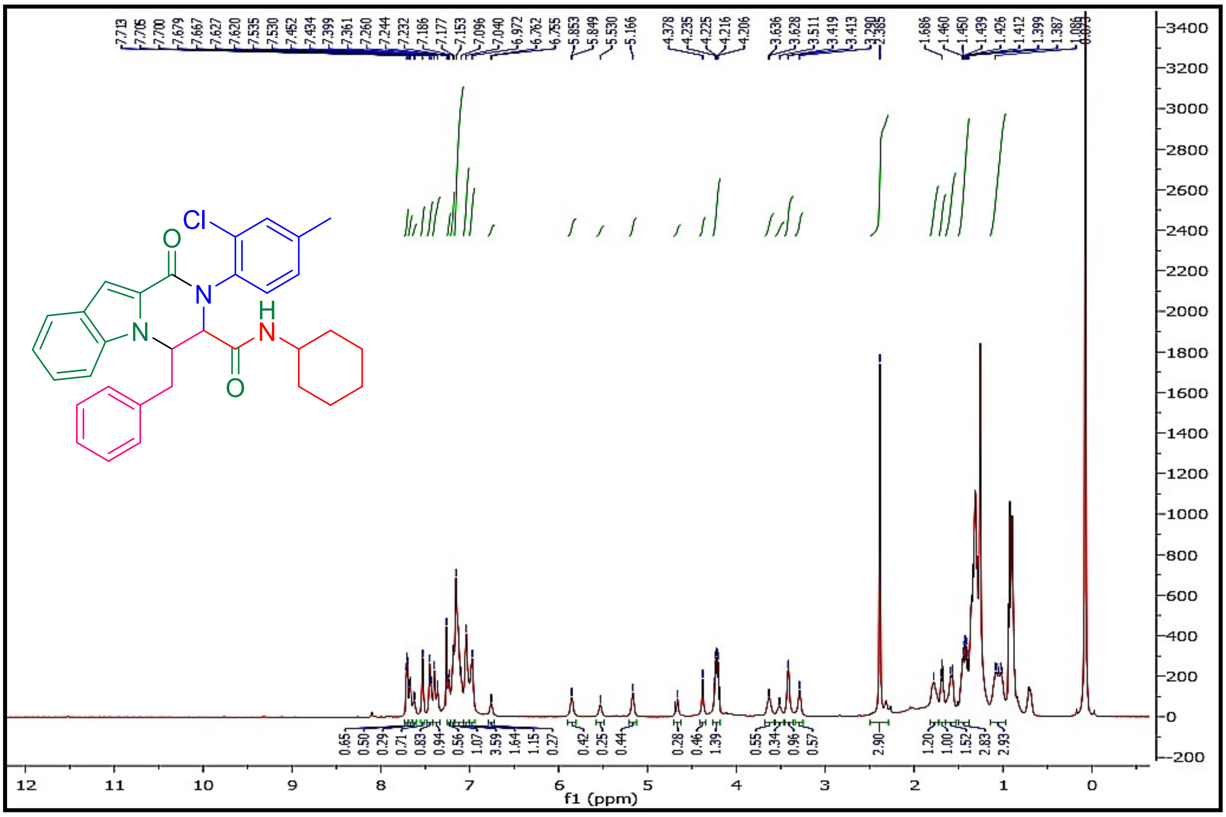

^{13}C-NMR (150 MHz, $CDCl_3$) 6n

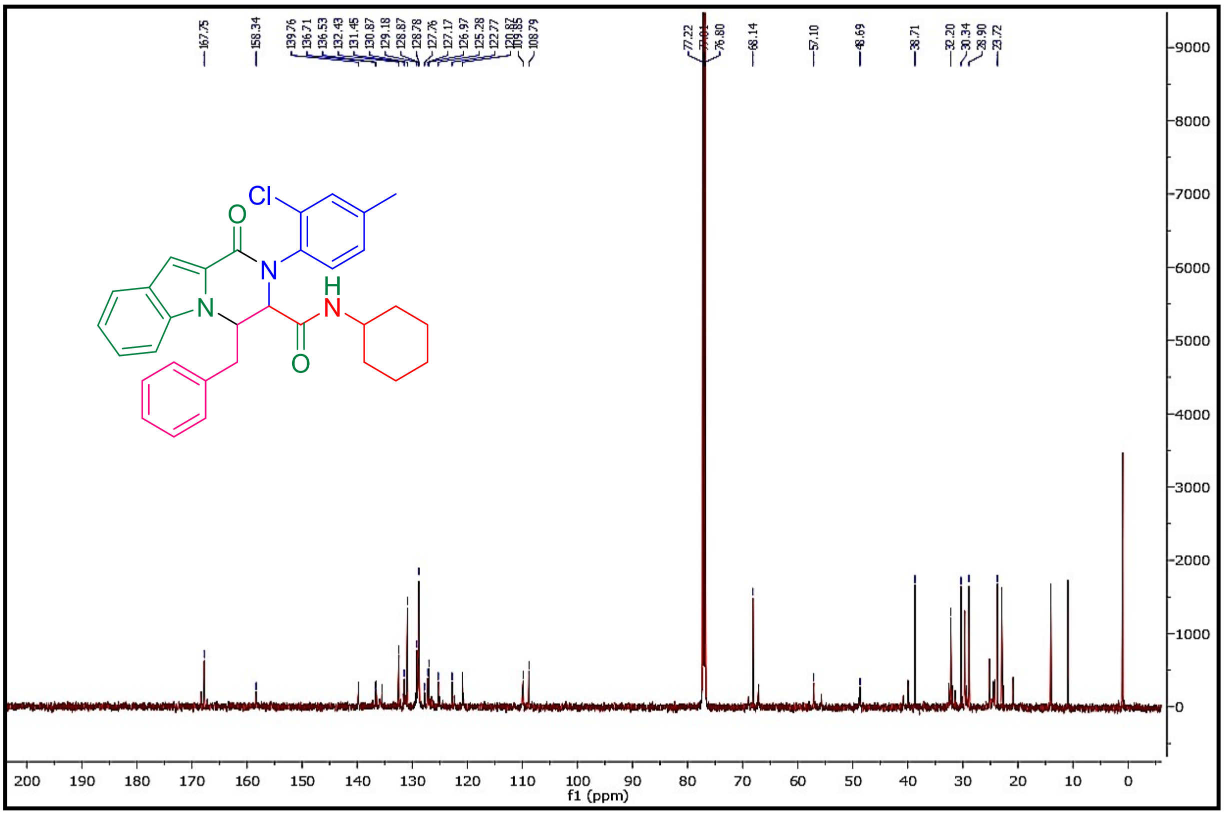

^{1}H-NMR (600 MHz, $CDCl_3$) 6o

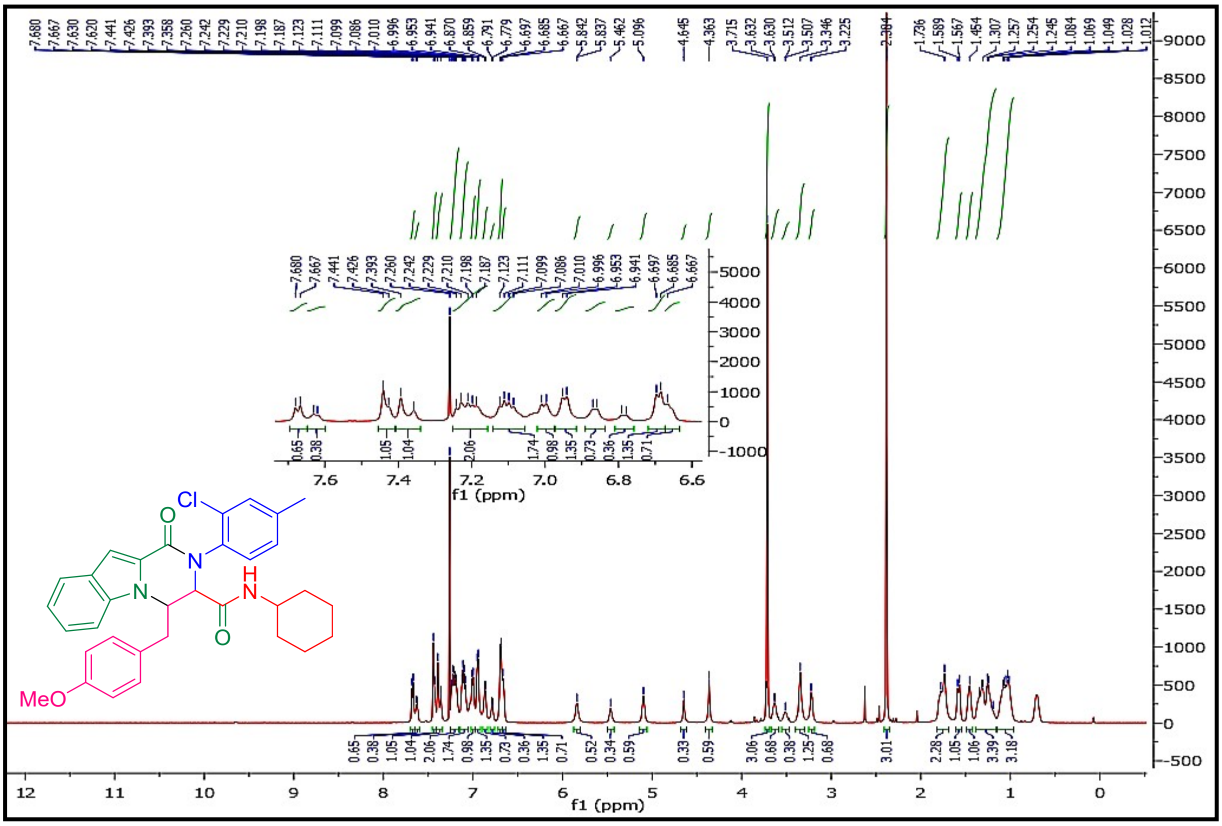

^{13}C-NMR (150 MHz, $CDCl_3$) 6o

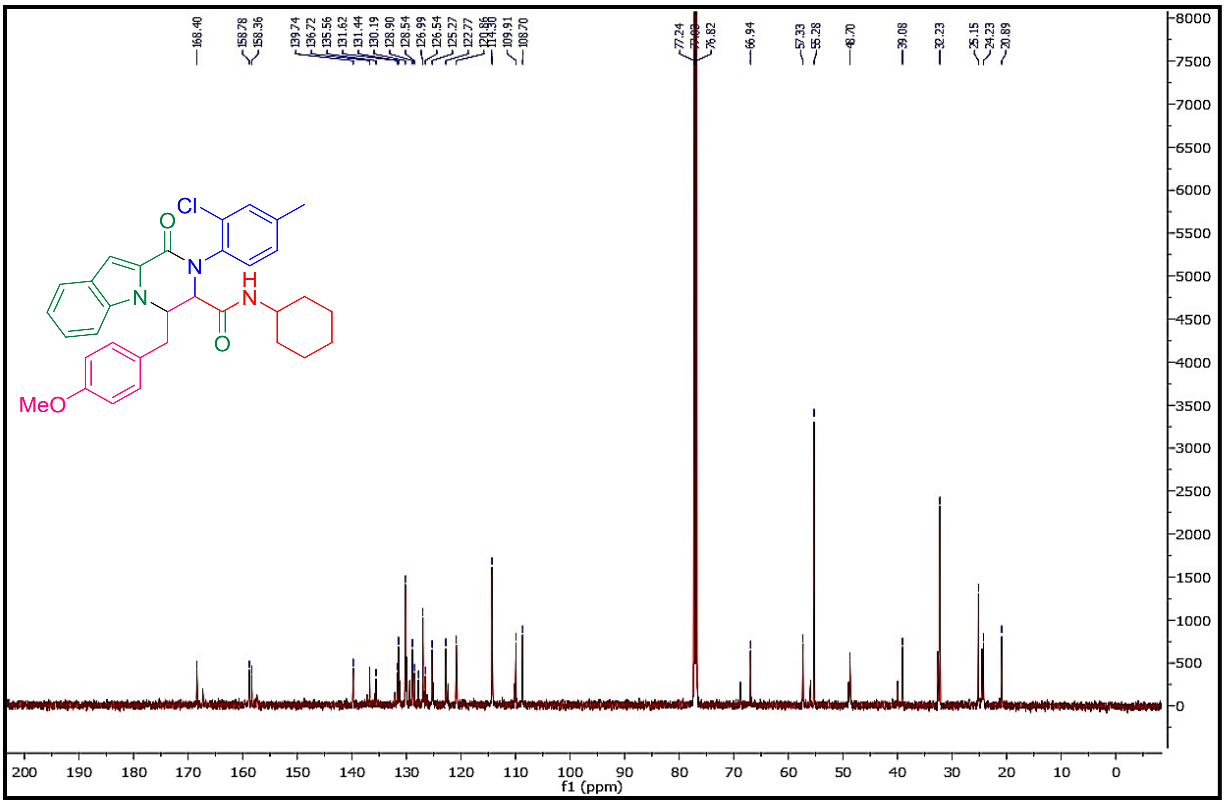

^{1}H-NMR (300 MHz, $CDCl_3$) 6p

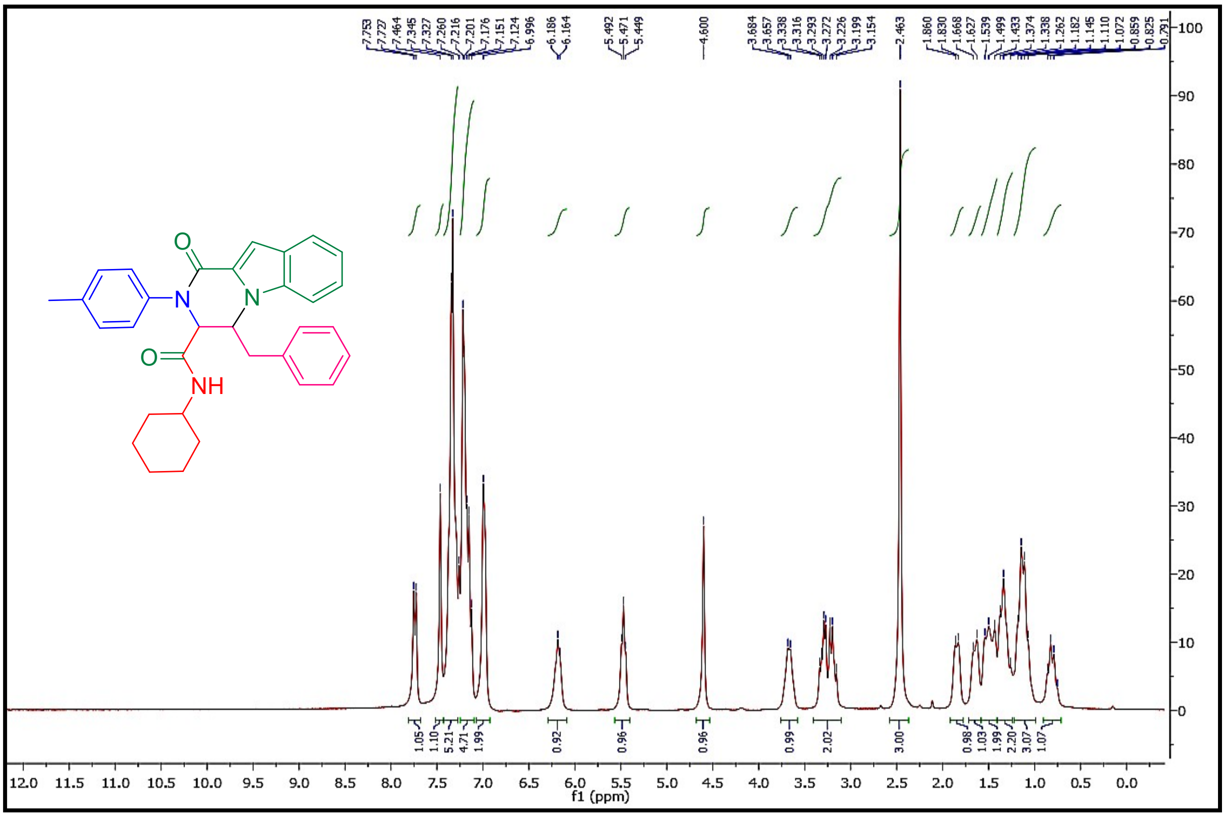

^{13}C-NMR (75 MHz) 6p

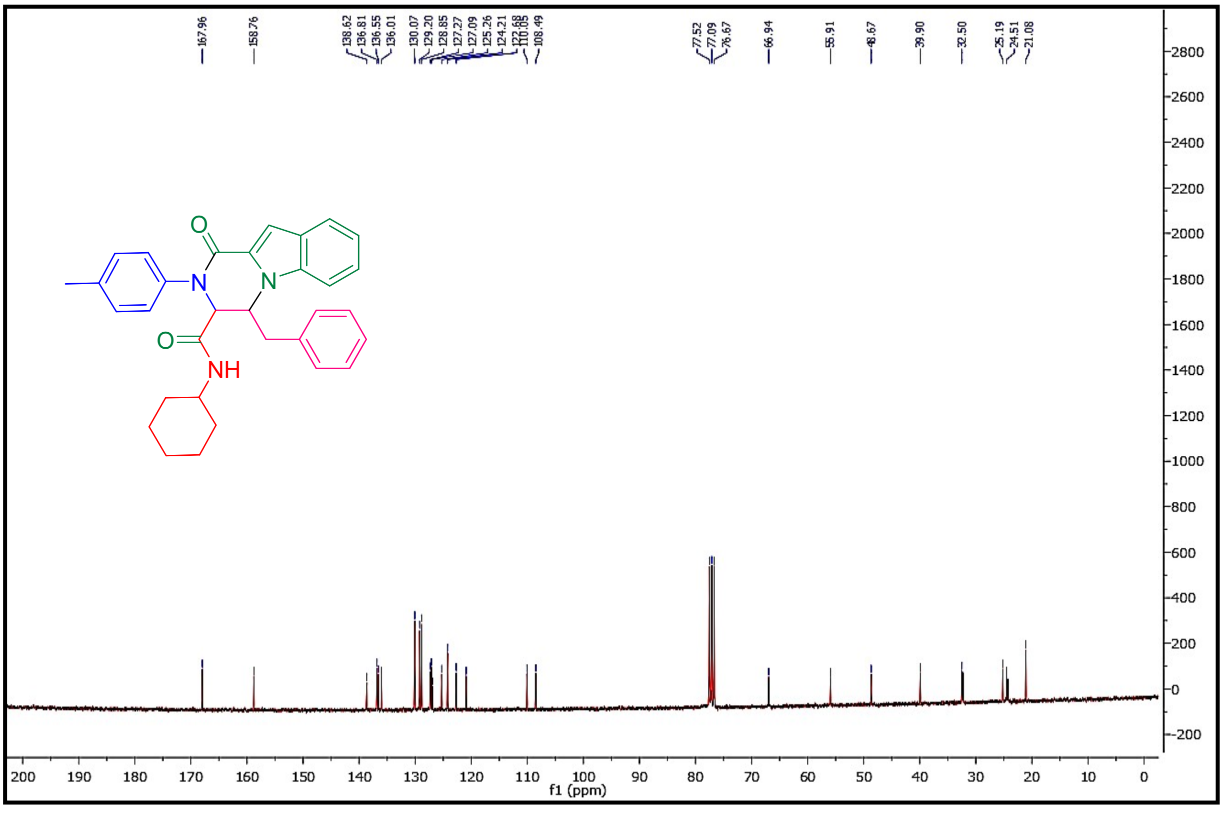

Crystal structure analysis

The X-ray diffraction data on **6b** needle-shaped crystals were collected with Cu-K_α radiation (λ = 1.54184 Å) using a Rigaku Oxford Diffraction XtaLABSynergy-R DW diffractometer equipped with a HyPix-ARC 150 Hybrid Photon Counting (HPC) detector. The data were measured at 100(2) K by using an Oxford Cryosystems open-flow nitrogen cryostat. Data collection, cell refinement, data reduction and analysis were carried out with the CrysAlisPro software package.[1] Numerical absorption correction based on gaussian integration over a multifaceted crystal model was applied to the data. The crystal structure was solved by direct methods using SHELXS-97,[2] and refined by a full-matrix least squares technique on F2 with SHELXL-2013[3] with anisotropic thermal parameters for all non-H atoms, except two disordered atoms (O3C and C32C). All H atoms were found in difference Fourier maps and refined isotropically, but in the final refinement cycles they were repositioned in their calculated positions and refined using a riding model in geometrically optimized positions with: C–H = 0.98–1.00 Å with Uiso(H) = 1.5Ueq(C). The lenghts of N–H bonds were fixed to 0.88 Å but H atoms of those groups were refined with isotropic thermal parameters. All figures were made using the DIAMOND program.[4] Analysis of the intra- and intermolecular interactions was performed using the program PLATON.[5]

Crystallographic data and important structure refinement parameters are summarized in Table 4 and the complete structural data have been deposited at the Cambridge Crystallographic Data Center (CCDC No. 2249611).

1 CrysAlis Pro (Rigaku Oxford Diffraction) *Yarnton, Engl.*, **2015**.

2 G. M. Sheldrick, *Acta Crystallogr. Sect. A Found. Crystallogr.,* 2008, **64**, 112.

3 G. M. Sheldrick, *Acta Crystallogr. Sect. C Struct. Chem.,* 2015, **71**, 3.

4 A. M. Dąbrowska, A. Adamczyk-Woźniak, I. D. Madura, *CrystEngComm,* 2022, **24**, 3586

5 A. L. J. Spek, *J. Appl. Crystallogr.,* 2003, **36**, 7.

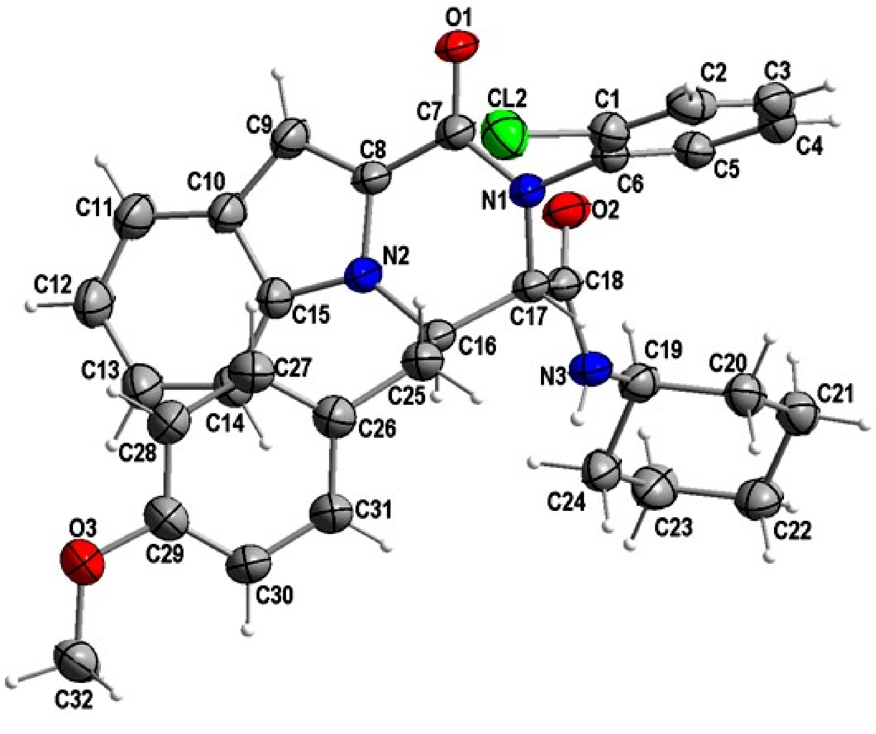

Crystal structure of the **6b** molecule showing the atom-numbering scheme and displacement ellipsoids drawn at the 50% probability level. The partially disordered molecule A was removed for clarity.

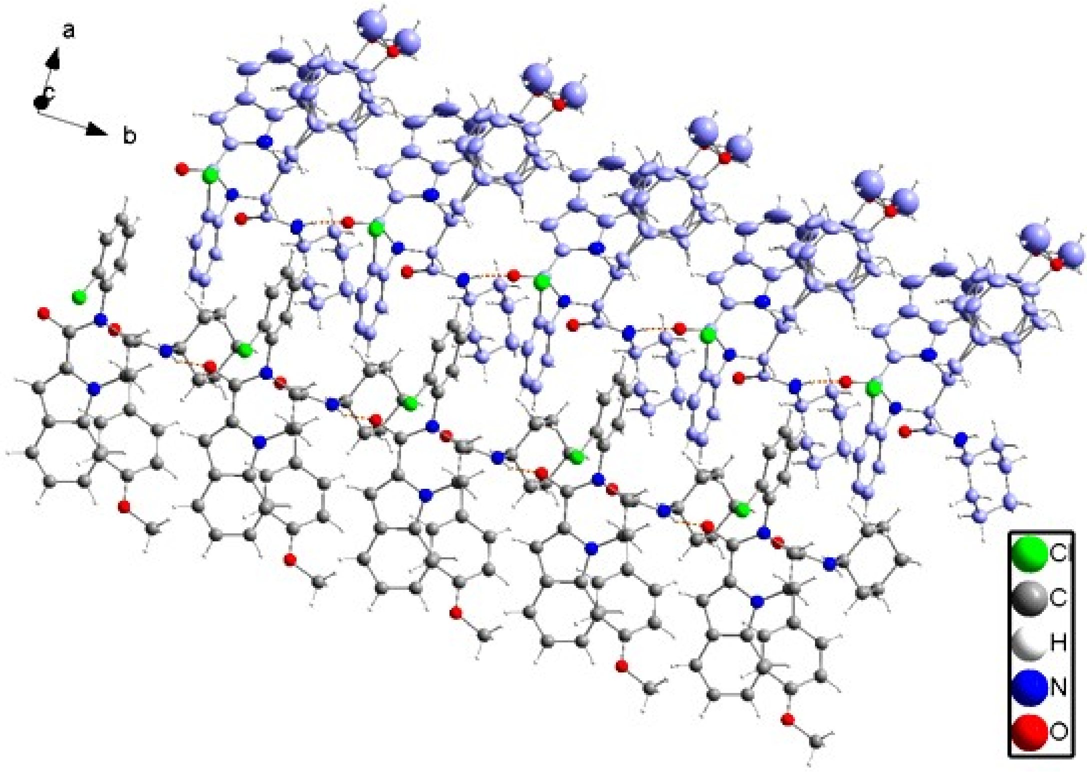

A part of crystal packing of **6b** showing the chain along b-axis formed by N-H···O hydrogen bonds (depicted as orange dashed lines). The carbon atoms of the partially disordered molecule A is presented in lilac.

Table S1. Hydrogen-bond geometry (Å, º).

D–H···A	*D*···A	H···A	D–H	D–H···A
164	2.867 (5)	2.01	0.88	N3A–H3A1···O1A^{i}
160	2.854 (5)	2.01	0.88	N3B–H3B1···O1B^{i}

Symmetry code: (i) *x*, *y*+1, *z*.

Table S2. The aromatic π–π stacking interactions distances (Å).

Cg···Cg	π–π stacking interactions
3.6965(17)	Cg1A ··· Cg1B
3.7408(17)	Cg1A··· Cg1B^{i}
3.7408(17)	Cg1A^{ii} ··· Cg1B
3.5383(16)	Cg2 ··· Cg2^{iii}

The centroids (Cg): C1A-C6A (Cg1A), C1B-C6B (Cg1B), C10B-C15B (Cg2).
Symmetry codes: (i) x, y+1, z, (ii) x, y-1, z, (iii) x, y+1, z,

Table S3. The C-H··· π interactions geometry (Å, º).

D–H···Cg	*D*···Cg	H···Cg	D–H	D–H···A
116	3.4549(16)	2.93	0.95	C14A–H14A··· Cg3A
105	2.9991(15)	2.92	0.95	C14B–H14B··· Cg3B
106	3.2470(15)	2.86	0.95	C14A–H14A··· Cg3C
139	3.7385(17)	2.94	0.98	C32B–H32E··· Cg4A^{iv}

The centroids (Cg): Cg3A (C26A-C31A), Cg3B (C26B-C31B), Cg3C (C26C-C31C), Cg4A (N2A-C15A).
Symmetry code: (iv) x-1/2, -y+1, z-1/2.

Table 4 Crystallographic data and structure refinement parameters for compound **6b**.

Crystal data	
Chemical formula	$C_{32}H_{32}ClN_3O_3$
M_w (g)	542.05
Crystal system, space group	Monoclinic, *P*2/n
Temperature (K)	100
a, b, c (Å)	26.155 (9), 6.9777 (18), 30.372 (14)
β (°)	100.04 (4)
V (Å^3)	5458 (4)
Z	8
Radiation type	CuK_α
µ (mm−1)	1.55
Crystal size (mm)	0.30 × 0.04 × 0.02
Data collection	
Diffractometer	XtaLAB Synergy R, DW system, HyPix-Arc 150
Absorption correction	Gaussian
Tmin, Tmax	0.548, 1.000
No. of measured, independent and observed [I > 2σ(I)] reflections	32621, 10338, 6331
R_{int}	0.093
$(\sin \theta/\lambda)_{max}$ (Å^{-1})	0.622
Refinement	
R[$F^2 > 2\sigma(F^2)$], ,	0.089
$wR(F^2)$	0.278
S	1.06
No. of reflections	10338
No. of parameters	742
No. of restraints	2
H-atom treatment	H-atom parameters constrained
$\Delta\rho_{max}$, $\Delta\rho_{min}$ (e Å^{-3})	0.80, −0.53
